# Clinical prediction models in psychiatry: a systematic review of two decades of progress and challenges

**DOI:** 10.1038/s41380-022-01528-4

**Published:** 2022-04-01

**Authors:** Alan J. Meehan, Stephanie J. Lewis, Seena Fazel, Paolo Fusar-Poli, Ewout W. Steyerberg, Daniel Stahl, Andrea Danese

**Affiliations:** 1 Department of Psychology, Institute of Psychiatry, Psychology & Neuroscience, King’s College London, London, UK; 2 Social, Genetic and Developmental Psychiatry Centre, Institute of Psychiatry, Psychology & Neuroscience, King’s College London, London, UK; 3 Yale Child Study Center, Yale School of Medicine, New Haven, CT 06520 USA; 4 Department of Child and Adolescent Psychiatry, Institute of Psychiatry, Psychology & Neuroscience, King’s College London, London, UK; 5 Department of Psychiatry, University of Oxford, Oxford, UK; 6 Early Psychosis: Interventions and Clinical-detection (EPIC) Lab, Department of Psychosis Studies, Institute of Psychiatry, Psychology & Neuroscience, King’s College London, London, UK; 7 OASIS Service, South London and Maudsley NHS Foundation Trust, London, UK; 8 Department of Brain and Behavioral Sciences, University of Pavia, Pavia, Italy; 9 National Institute for Health Research, Maudsley Biomedical Research Centre, South London and Maudsley NHS Foundation Trust, London, UK; 10 Department of Biomedical Data Sciences, Leiden University Medical Centre, Leiden, The Netherlands; 11 Department of Public Health, Erasmus MC, Rotterdam, The Netherlands; 12 Department of Biostatistics and Health Informatics, Institute of Psychiatry, Psychology & Neuroscience, King’s College London, London, UK; 13 National and Specialist CAMHS Clinic for Trauma, Anxiety, and Depression, South London and Maudsley NHS Foundation Trust, London, UK

**Keywords:** Psychology, Psychiatric disorders

## Abstract

Recent years have seen the rapid proliferation of clinical prediction models aiming to support risk stratification and individualized care within psychiatry. Despite growing interest, attempts to synthesize current evidence in the nascent field of precision psychiatry have remained scarce. This systematic review therefore sought to summarize progress towards clinical implementation of prediction modeling for psychiatric outcomes. We searched MEDLINE, PubMed, Embase, and PsychINFO databases from inception to September 30, 2020, for English-language articles that developed and/or validated multivariable models to predict (at an individual level) onset, course, or treatment response for non-organic psychiatric disorders (PROSPERO: CRD42020216530). Individual prediction models were evaluated based on three key criteria: (i) mitigation of bias and overfitting; (ii) generalizability, and (iii) clinical utility. The Prediction model Risk Of Bias ASsessment Tool (PROBAST) was used to formally appraise each study’s risk of bias. 228 studies detailing 308 prediction models were ultimately eligible for inclusion. 94.5% of developed prediction models were deemed to be at high risk of bias, largely due to inadequate or inappropriate analytic decisions. Insufficient internal validation efforts (within the development sample) were also observed, while only one-fifth of models underwent external validation in an independent sample. Finally, our search identified just one published model whose potential utility in clinical practice was formally assessed. Our findings illustrated significant growth in precision psychiatry with promising progress towards real-world application. Nevertheless, these efforts have been inhibited by a preponderance of bias and overfitting, while the generalizability and clinical utility of many published models has yet to be formally established. Through improved methodological rigor during initial development, robust evaluations of reproducibility via independent validation, and evidence-based implementation frameworks, future research has the potential to generate risk prediction tools capable of enhancing clinical decision-making in psychiatric care.

## Introduction

The recent rise of precision psychiatry has provided new avenues towards individualized risk estimation for a range of clinical outcomes. Unlike explanatory approaches that compare average risks between *groups* in a population, prediction models seek to maximize the ability to accurately separate individuals with and without the outcome of interest (discrimination) and promote agreement between predicted and observed outcome frequencies (calibration) [1]. The resulting multivariable risk profiles can identify an individual’s unique probability for the current presentation of a psychiatric condition (diagnostic models), its future onset or course (prognostic models), or likely response to associated treatment (predictive models) [2, 3]. Prediction models can therefore inform clinical decisions by supporting structured tools (e.g., risk calculators, clinical prediction rules) that classify patients into discrete subgroups (stratification) or individualized care pathways (personalization).

A wide array of multivariable prediction models have been successfully integrated into routine practice across medicine, and regularly support national clinical guidelines around treatment allocation [4–6]. The multifactorial, heterogeneous, and highly comorbid nature of psychiatric phenotypes, coupled with a greater reliance on subjective and indirect symptom measurement, has contributed to slower progression of prediction science within psychiatry compared to other clinical specialties. Despite these challenges, the burgeoning field of ‘precision psychiatry’ offers similarly promising opportunities to enhance diagnosis, prognosis, and prediction of treatment response [3, 7].

Using current optimal benchmarks [8], this systematic review sought to comprehensively map current progress towards clinical application of prediction models within psychiatry by evaluating the extent to which the models published to date successfully: (i) avoid bias and overfitting; (ii) show generalizability across different populations; and (iii) provide evidence of real-world clinical utility. In doing so, this review updated and expanded on previous systematic investigations that were either disorder-specific [9–11] or focused on a subset of published studies that included evidence of internal or external validation [12], to consider both the full breadth of diagnoses within the psychiatric literature and key methodological characteristics beginning from the earliest stages of configuration and development. At the same time, our focus on the ultimate endpoint of real-world implementation sought to identify successful examples of psychiatric prediction tools in clinical practice and, failing that, highlight future research directions that may serve to maximize the translational potential of the most promising models within the literature.

## Methods

### Search strategy

This review followed the Preferred Reporting Items for Systematic reviews and Meta-Analyses (PRISMA) statement (see Supplementary Table S1) and was pre-registered on PROSPERO (CRD42020216530). Embase, MEDLINE (via Ovid and PubMed), and PsycINFO databases were searched (i) from inception to September 30, 2020, for (ii) peer-reviewed journal articles that were (iii) published in English, which developed or validated models to predict the onset (diagnostic), illness course (prognostic), or treatment response (predictive) for individuals with non-organic psychiatric conditions (defined using diagnostic criteria or validated psychometric cut-offs). A complete list of search terms is presented in Appendix 1 of Supplementary Material. All search results including titles and abstracts were imported into EndNote X9 for review. Following duplicate removal, titles and abstracts were consecutively screened, with full texts of potentially eligible articles evaluated against selection criteria. Reference lists of retained articles were also hand-searched to identify additional studies.

### Selection criteria

Inclusion and exclusion criteria are described and justified in detail in Appendix 2 of Supplementary Material. In brief, we included studies that: were multivariable (≥2 predictors); tested individual-level predictive performance (e.g., discrimination, calibration); examined individual patient data (as opposed to meta-analyses); and contained a majority (>50%) of sociodemographic and/or clinical predictors, due to noted statistical and pragmatic concerns around the generalizability of psychiatric prediction models based solely on one modality of biological variables, such as neuroimaging or genetics [13–16].

### Evaluation of prediction models

Informed by the prediction modeling literature, we applied three main criteria to evaluate progress towards clinical implementation among identified models (full details in Appendix 3 of Supplementary Material). First, we tested whether models attempted to minimize bias (inclusion of systematic error) and overfitting (where risk coefficients incorporate sample-specific error variance alongside the ‘true’ effect), both of which can produce inaccurate or inflated estimates of predictive accuracy [3]. Addressing these threats requires sufficiently large sample sizes (in particular, an adequate number of outcome events relative to the number of parameters among candidate predictors) and appropriate predictor selection strategies, with a priori selection informed by current empirical and/or clinical evidence recommended over hypothesis-free stepwise or univariate approaches [17, 18]. Second, we tested whether models attempted to maximize generalizability (i.e., consistent and reliable predictions in new individuals), either within the development sample itself (internal validation) or, ideally, using wholly independent data (external validation) [19, 20]. Finally, we tested whether models attempted to demonstrate clinical utility in real-world settings, either by statistically modeling their likely benefit to current practice or, ideally, randomized impact studies that quantified changes in patient outcomes following implementation [1, 21].

### Data extraction

Informed by the CHecklist for critical Appraisal and data extraction for systematic Reviews of prediction Modeling Studies (CHARMS) [22] and Transparent Reporting of a multivariable prediction model for Individual Prognosis Or Diagnosis (TRIPOD) statement [2], one reviewer (AJM) completed extraction while a second (SJL) independently verified a random 20% subset of full-text articles. Uncertainties were referred to an expert third rater (DS). Full information on extracted data is provided in Appendix 4 of Supplementary Material.

### Quality assessment

For each individual model development and external validation analysis, the Prediction model Risk Of Bias ASsessment Tool (PROBAST; see Appendix 5 of Supplementary Material) was used to assess risk of bias (ROB), or the potential for inflated model performance estimates due to shortcomings in design/analysis [8]. Agreement between independent ratings from two reviewers (AJM; SJL) on a random 20% subset of ratings (20.6%; 78/378) was quantified using Cohen’s weighted kappa (*κ*) coefficient.

## Results

**Fig. 1:**
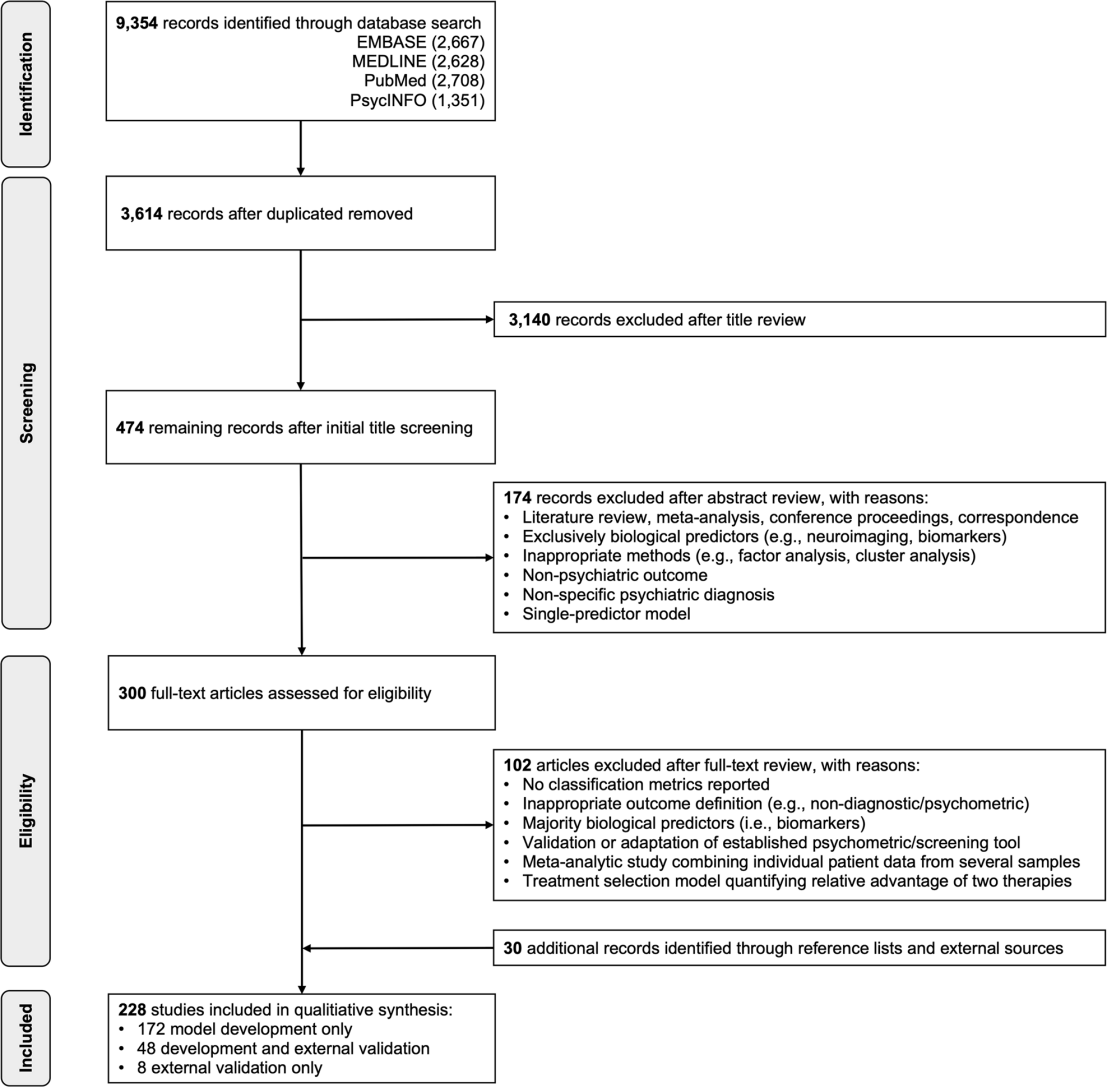
PRISMA flow diagram of study selection.

**Fig. 2:**
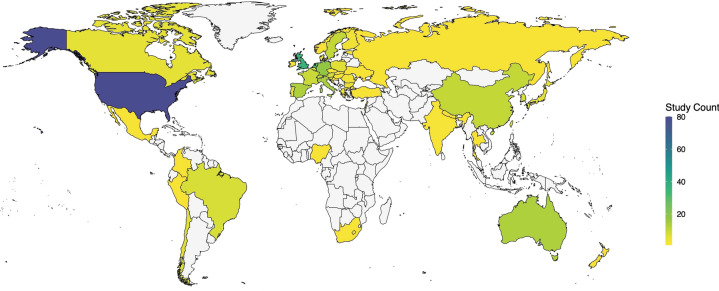
Geographic coverage of reviewed study samples. A total of 21 of the 228 studies (9.2%) incorporated data from several countries (range: 2–24) via multi-national samples or research consortia and thus are counted multiple times, yielding a total count of 369. World map coordinates retrieved from *maps* and *ggplot2* packages in R.

**Fig. 3:**
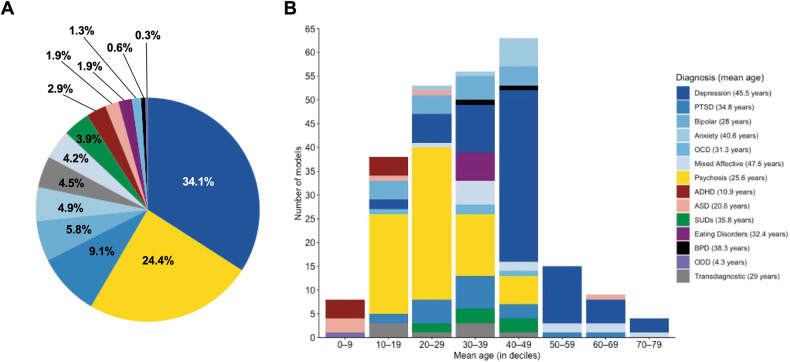
Psychiatric outcomes among prediction models. **A** Distribution of psychiatric diagnoses across individual prediction models (*n* = 308). **B** Distribution of psychiatric diagnoses across deciles of mean sample age, where data were available (*n* = 246). ADHD attention-deficit/hyperactivity disorder, ASD autism spectrum disorder, BPD borderline personality disorder, OCD obsessive-compulsive disorder, ODD oppositional defiant disorder, PTSD post-traumatic stress disorder, SUDs substance use disorders. ‘Mixed affective’ models tested some combination of anxiety, depressive, and/or manic symptoms in a single outcome, while ‘transdiagnostic’ models consolidated several externalizing and/or internalizing diagnoses. Due to rounding, percentages may not add up to 100%.

Having screened 3614 unique records, 228 studies met inclusion criteria (Fig. 1), from which 308 individual prediction models were extracted (Supplementary Table S2). 69.6% (215/308) of these models were prognostic (examining onset [113/215] or course-of-illness outcomes [102/215]), 21.4% of models (66/308) were diagnostic, and 8.8% of models (27/308) were predictive. 50 countries were represented among included samples (Fig. 2), with 92% (46/50) of these considered high- or upper-middle-income economies based on World Bank 2020/21 income classifications [23]. Categorizing each outcome based on its overarching diagnostic domain (Fig. 3A, B), depression-related outcomes were most common, comprising just over a third of all models (34.1%; 105/308), followed by psychosis (24.4%; 75/308) and post-traumatic stress disorder (9.1%; 28/308).

### Bias and overfitting

**Fig. 4:**
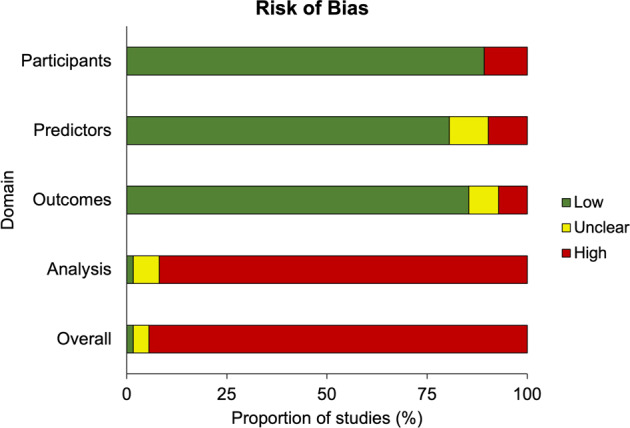
Risk of bias among prediction models. Domain-level summary of risk of bias for all developed prediction models within the reviewed literature (*n* = 308). For individual PROBAST domain ratings across all development and external validation analyses, see Supplementary Table S3.

Risk of bias was high for 94.5% of model development analyses (291/308; Fig. 4) and 68.6% of external validation analyses (48/70; Supplementary Table S3), with substantial inter-rater agreement on these quality ratings (93.6%; weighted κ = 0.79). These substantial levels of bias risk were due to one or more of the following reasons:

During initial model development, risk of bias was highest within the PROBAST’s ‘analysis’ domain (91.9%; 283/308 models). The most common analytic concern was an insufficient number of outcome events relative to the parameters among candidate predictors, or ‘events per variable’ (EPV; see Appendix 3 of Supplementary Material). Only 8.3% of all studies (19/228) reported considering EPV during model development or validation, and none explicitly reported the estimated EPV for their sample. Among individual prediction models, 77.3% (238/308) had sufficient information on the number of (i) events and (ii) considered predictors to enable derivation of approximate EPV values (median = 3.98; range = 0.01–692.4). Of these models, only 26.9% (64/238) met the widely adopted benchmark of EPV ≥ 10 based on these estimates, with only 16.8% (40/238) surpassing the threshold of EPV ≥ 20 advocated in more recent statistical literature [18].

Several predictor selection strategies utilized by these studies were also prone to bias. From 220 model development studies, only 8.6% (19/220) selected candidate predictors a priori based on existing research evidence, clinical knowledge, or previous models, which minimizes the risk of bias [17]. Instead, most development studies utilized at least one data-driven method to reduce the number of predictors. In brief, 35% of model development studies (77/220) relied on automated feature selection procedures within their chosen statistical method (e.g., regularization, variable importance ranking, recursive feature elimination), 26.8% (59/220) used automated forward, backward and/or stepwise selection to prune variables, and 24.5% (54/220) selected predictors based on significant bivariate associations with the outcome, all of which can bias risk coefficients and, in turn, performance estimates (see Appendix 3 of Supplementary Material).

Half of all individual prediction models (51%; 157/308) were estimated using regression-based methods, with 37.6% of these (59/157) applying coefficient shrinkage (e.g., Least Absolute Shrinkage Selector Operator, Elastic Net) to alleviate overfitting via reduced variance. 19.8% of models (61/308) utilized a specific machine learning (ML) approach (e.g., random forest, support vector machines, neural networks). Of note, 20.8% of all models (64/308) were identified by comparing several statistical/ML algorithms before selecting the approach that optimized predictive performance for their dataset, while 5.2% of models (16/308) used ensemble methods that consolidated performance from several classifiers in one optimally weighted average.

Finally, key components of predictive performance were poorly or inconsistently reported. Discrimination was almost always evaluated: 88% of models (271/308) reported the *c*-index or area-under-the-curve (AUC), while at a minimum, the remaining models (37/308) described accuracy, sensitivity and/or specificity at a specific classification threshold (although only 24.3% of these [9/37] explicitly reported the threshold used to dichotomize probabilities). However, only 22.1% of models (68/308) tested calibration. Of these, 52.9% (36/68) presented a calibration plot, as recommended, while 22.1% (15/68) only reported the Hosmer-Lemeshow test, despite this being an insufficient measure of calibration when considered alone.

### Generalizability

**Fig. 5:**
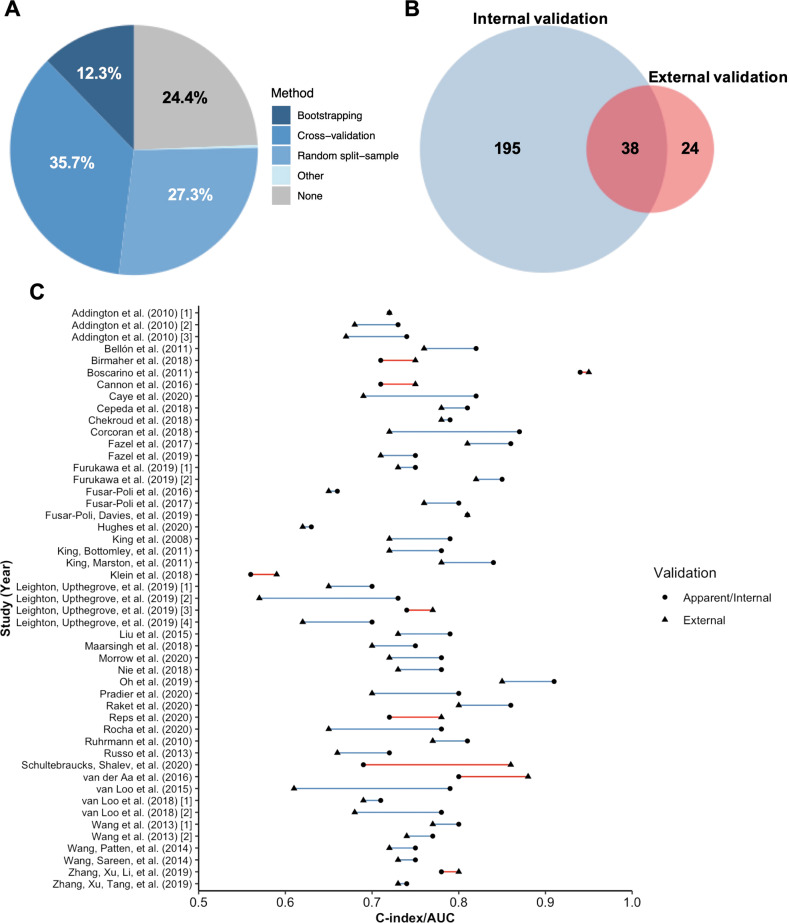
Internal and external validation efforts and performance among prediction models. **A** Summary of internal validation methods among all prediction models (*n* = 308). **B** Overlap between internal and external validation efforts. **C** Distribution of *c*-indices/AUCs in the development sample (with or without internal validation) and external validation sample for models where both were reported (*n* = 49). Blue lines denote superior performance in the development sample, while red lines indicate superior external performance. Where a model has been validated in more than one external sample, an average *c*-index/AUC across these samples has been derived.

Three-quarters of all prediction models (75.6%; 233/308) utilized at least one internal validation technique within their development sample (Fig. 5A). However, only 11.4% of all models (35/308), or 15% of internally validated models (35/233), were judged to have performed internal validation to a sufficient statistical standard (see Appendix 4 of Supplementary Material). The main source of poor internal validation was use of random split-sampling (36.1% of all purportedly internally validated models; 84/233), which is not recommended over resampling approaches such as cross-validation and bootstrapping [20]. Furthermore, only 22.7% of cross-validated models (25/110) and 23.7% of bootstrapped models (9/38) satisfied criteria for adequate internal validation by nesting all feature selection and tuning procedures within their validation framework, or by pre-selecting predictors to avoid the need for variable selection entirely.

20.1% (62/308) of all models were externally validated in a wholly independent sample, reported either in the same study or a subsequent publication. Of these models, 61.3% (38/62) attempted prior internal validation in the development sample (Fig. 5B), although only 31.6% of these efforts (12/38) were judged to meet statistical standards (see above). 79% (49/62) of externally validated models reported a *c*-index/AUC for both development and validation samples, with 77.6% (38/49) of these recording poorer out-of-sample discrimination and 22.4% (11/49) reporting equivalent or improved external performance (Fig. 5C). However, only four of these 11 models utilized validation datasets featuring >100 participants with outcome, as recommended to ensure sufficient power [24].

### Clinical utility

Only two prediction models (both predicting psychosis risk) assessed clinical usefulness using decision-curve analyses to quantify the potential ‘net benefit’ to routine practice [25, 26]. For one of these models, two implementation studies have subsequently been published for a derived risk calculator, providing preliminary evidence of technical feasibility and clinical adherence [27, 28].

With no other assessments of clinical utility, we used key study characteristics to consider whether development samples accurately reflected their intended clinical populations. First, from 79.9% of models with available data (246/308), we examined mean sample ages for each psychiatric condition (Fig. 3B). The resulting diagnostic variation generally reflected developmental trends for each disorder [29, 30]. For example, mean ages for ADHD (*M* = 10.9, range = 8.3–17) and psychosis prediction studies (*M* = 25.6, range = 15.1–44) reflected the established onset and peak prevalence of these disorders during childhood and young adulthood, respectively. In contrast, the age range of samples predicting depression-related outcomes was notably wider, with a greater focus on middle and older adulthood (*M* = 45.5, range = 15.6–78). Second, we specifically examined prognostic models with available age data that sought to predict the onset of a psychiatric condition. Of these, only 40% (36/90) utilized youth samples (<24 years); however, this is the age range where up to 75% of all psychiatric disorders have their initial onset [30].

## Discussion

Our systematic review, capturing over two decades of research, presents a clear trend of improvement in precision psychiatry over time. Growth in the field has been exponential, with around 70% of all reviewed studies published in the past five years, while the literature base has expanded to include a wide array of psychiatric diagnoses and course-of-illness measures. Development and validation procedures have also grown more robust, with several extensively validated models exhibiting similar predictive accuracy to established risk algorithms for cardiovascular disease and cancer [4, 5]. Most promising of all, recent evidence suggests the potential of some models to support real-world decision-making – specifically, estimation of psychosis risk [28]. Despite these achievements, however, we found virtually no peer-reviewed evaluations of whether implementing these models in routine psychiatric care conferred sufficient improvements to patient outcomes. To contextualize progress in the field to date, we identified key challenges around efforts to alleviate bias and overfitting, ensure generalizability to new individuals, and demonstrate meaningful clinical utility.

First, we found potentially high risk of bias and overfitting across the literature, primarily driven by errors or oversights during model configuration. The majority of studies had insufficient outcome events relative to the number of predictors, used bias-prone variable selection strategies, and failed to assess key aspects of predictive performance, particularly calibration. These issues were often exacerbated by statistical methods that encouraged overfitting, including: regression models that did not apply shrinkage to address overfitting (or did so despite insufficient EPV) [31]; ML models that lacked the sample sizes required for clinical databases (where predictors typically only demonstrate small-to-moderate effect sizes) to capitalize on their computational power [32]; and studies that risked ‘over-optimization’ to their data by prioritizing the best-performing model from several alternative statistical approaches, instead of the average performance across methods [33]. The extent of these biases may also reflect the relatively low prevalence rates, broad clinical presentations, and multivariable and multimodal risk profiles that characterize psychiatry compared to other medical fields.

By increasing the risk of overfitting, misspecification, and inaccurate estimates of predictive ability, these methodological limitations make it difficult to forecast the likely external performance or clinical benefit of most prediction models. Evidence-based pre-selection of predictors in future studies may help to ensure sufficient EPV while reducing reliance on potentially biased automated selection methods [17]. Recently developed methods for estimating the minimum sample size for a prediction model, which no reviewed study utilized, may also help to reduce reliance on blanket ‘rules of thumb’ around EPV [34]. Similarly, although machine learning techniques can model complex relationships in high-dimensional data with minimal pre-specification, recent evidence suggests that ML methods are ‘data hungry’ [32], and its presumed superiority over conventional regression models may be overstated in clinical settings where data have a relatively low signal-to-noise ratio [35]. This underscores the importance of prioritizing analyses that are, above all, appropriate for the available data. Pre-registered analytic protocols (including a priori predictors) should be promoted to discourage post-hoc reconfiguration based on sample-specific associations. Current quality assessment tools (i.e., PROBAST), though stringent, can also provide structured guidance for developing robust prediction models that avoid common biases [8].

Second, regarding generalizability, as only one-fifth of models underwent external validation, reported performance estimates are likely to overstate likely predictive ability in new individuals across diverse geographic, cultural, and/or temporal contexts. Moreover, within development samples, most internal validation procedures were insufficiently rigorous to reliably alleviate overfitting. Low- and middle-income economies were particularly under-represented among validation samples, limiting insight into the global applicability of psychiatric prediction models. Given the diversity of sociodemographic and health-system characteristics worldwide, universal generalizability may be an unrealistic goal for precision psychiatry, as evidenced by the abundance of region- and population-specific risk tools in other medical specialties [4]. However, despite the availability of methods to contextualize losses or gains in prediction during validation, by comparing case-mix distributions between samples [19], only two external validation analyses presented these metrics [36, 37].

Going forward, growing numbers of multi-center studies and inter-cohort collaborations may facilitate independent validation prior to (or in tandem with) initial model development. To combat potential publication biases in favor of positive results, dissemination of all robust validation efforts, including those where external performance is unsatisfactory, should be encouraged. Similarly, models that underperform in external samples are often discarded in favor of an entirely new model that offers better prediction for that specific dataset, resulting in numerous competing yet unvalidated prediction models for the same outcome [3]. Instead of continual redevelopment, studies should first seek to statistically ‘update’ existing models by re-calibrating, adjusting weights, or considering additional predictor/interaction terms, thus building on the original model’s predictive ability while better reflecting the specific composition of the validation sample [38].

Third, we identified just one model whose potential clinical utility was formally evaluated using implementation studies [27, 28]. For all other models, success was effectively determined based on statistical accuracy rather than tangible improvements in care. Most published prediction models in the medical literature never reach clinical practice, and the same appears true for psychiatry to date. This obvious translational gap is particularly conspicuous given that many models appear poorly suited to the clinical decision they aim to support; for example, most models seeking to predict new psychiatric diagnoses did not consider critical onset periods from childhood to young adulthood [30]. More broadly, as we have demonstrated, the generalizability of precision psychiatry beyond homogenous populations in high-income economies remains largely untested, while inappropriate application of overly complex algorithms to small and unrepresentative datasets can inhibit transparency, reproducibility, and ultimately, implementation in routine psychiatric care, with little performance benefit in return [17].

Greater attention should be paid to later stages of the implementation pathway within precision psychiatry. For models with robust external validity, randomized impact studies are needed to formally compare patient outcomes between clinicians guided by their decisions vs care as usual [21]. Coherent psychiatry-specific implementation frameworks are needed to provide pragmatic guidance around these procedures [12]. Increased cognizance of the pragmatic goals of a prediction tool, and optimization to the clinical decision of interest from the outset of model-building, is also crucial to maximize the likelihood of successful translation. This involves selecting samples that reflect the target population, predictors that are routinely available within existing workflows to minimize clinician burden, outcomes linked to meaningful care decisions (e.g., admission, treatment allocation), and statistical analyses that support interpretable, replicable risk algorithms, whose outputs offer cost-effective advantages over current practice [3]. Transparency is of particular importance given the ‘black box’ nature of many ML approaches; here, the link between the model’s predictions and the eventual recommended care decision is often opaque, limiting understanding and, in turn, acceptance among clinicians and patients [39]. For these more complex models, a balance of interpretability and accuracy is increasingly advocated, with methods for deriving inherently ‘interpretable’ or explainable’ ML or artificial intelligence models gaining prominence [40, 41]. Engagement with relevant stakeholders (i.e., patients, practitioners, policy-makers) can also help to identify key clinical considerations and challenges, evaluate interpretability, and thus aid development of more patient-centered prediction models [42].

### Strengths and limitations

The review’s strengths include a comprehensive search of several databases, extensive extraction and derivation of key prediction model characteristics, and formal appraisal of bias using specialized quality assessment tools. However, we acknowledge several limitations.

First, our search focused on English-language studies and did not consider gray literature, with initial title and abstract screening completed by a single reviewer and independent verification of extracted data only completed for a random subset of studies. These actions may have excluded potentially relevant models and, in particular, influenced our reported geographical representation. Nevertheless, considering the broad search terms and large number of studies in the final review, we believe the risk of inappropriate exclusions – and, in turn, significant alterations to our conclusions – is low. Second, wide variation in outcome definitions precluded meta-analysis of performance metrics for specific diagnoses. Third, models that drew predominantly on genetic and neuroimaging data were excluded, based on consistent methodological and practical limitations in the existing literature that were likely to inhibit their reproducibility and integration into real-world practice (see Appendix 2 of Supplementary Material) [14, 15]. We also accept that biological datasets, which are typically more complex and high-dimensional than clinical databases, are thus better-suited to ML methods, such that ML-based prediction models within psychiatry may be somewhat under-represented here. However, as biological data collection during psychiatric assessment becomes more common, particularly in specialized settings, reliable integration of these variables into multimodal prediction tools will become more feasible.

Finally, and of particular note, although we evaluated all prediction models using accepted current benchmarks via the PROBAST, we accept that its ‘worst score counts’ rubric may paint an unduly pessimistic picture of the literature. Additionally, rating recommendations for some PROBAST criteria – particularly around sample size/EPV and predictor selection – were devised with traditional regression-based models in mind [43]. Uniform application of current rating guidelines may therefore unfairly penalize newer regularized or ML-based techniques, whose unique statistical features may mitigate the potential risk of bias and overfitting from these methodological decisions. At the same time, empirical evidence for the ability of ML models to overcome small sample sizes/EPVs, or better handle large numbers of predictors compared to regression approaches, remains scarce [32, 35, 44], suggesting that current PROBAST criteria, although relatively restrictive, may still provide a useful indication of their methodological quality. However, having recognized a need for additional PROBAST items to sufficiently evaluate bias in prediction models based on ML and artificial intelligence techniques, efforts are currently underway to develop an extension of the original tool for these specific modeling methods [45].

## Conclusions

Despite the rapid proliferation of precision psychiatry, the current evidence base has yet to fully realize its potential to inform diagnostic, prognostic, and predictive goals for patients, clinicians, and policymakers alike. Although unique theoretical and contextual challenges have historically slowed progress in prediction science for this branch of clinical medicine, improved methodological rigor can offer solutions to build on these promising foundations. Specifically, future research should seek to prioritize clinical relevance over statistical sophistication during initial model development, promote extensive validation in a variety of external samples, and employ implementation pathways to determine the pragmatic utility of the most robust models. This framework for successful prediction modeling will help to support widespread application of clinically useful tools that can support decision-making, enhance patient care, and promote more rational allocation of finite healthcare resources.

## Supplementary information

Supplementary Material
