# Supplementary material for: Clinical prediction models in psychiatry: a systematic review of two decades of progress and challenges
Source: Mol Psychiatry. 2022 Apr 1;27(6):2700–8. doi: 10.1038/s41380-022-01528-4 (PMC9156409; doi:10.1038/s41380-022-01528-4)
Supplement: Supplementary file 1 — Supplementary Material [file 41380_2022_1528_MOESM1_ESM.docx]

**Supplementary Material**

**Clinical prediction models in psychiatry:**

**A systematic review of two decades of progress and challenges**

| **Supplementary Methods** |  |
| --- | --- |
| **Appendix 1.** Search strategy and terms................................................................................................. | Page 2 |
| **Appendix 2.** Inclusion and exclusion criteria........................................................................................ | Page 3 |
| **Appendix 3.** Criteria for prediction model evaluation.......................................................................... | Page 4 |
| **Appendix 4.** Data extraction................................................................................................................. | Page 5 |
| **Appendix 5.** Quality assessment and risk of bias items........................................................................ | Page 8 |
| **Supplementary Tables** |  |
| **Table S1.** PRISMA checklist................................................................................................................. | Page 9 |
| **Table S2.** Characteristics of included prediction modelling studies..................................................... | Page 11 |
| **Table S3.** Quality assessment (risk of bias) ratings for reviewed studies............................................. | Page 47 |
| **Supplementary References............................................................................................................................** | **Page 53** |

**Appendix 1. Search strategy and terms**

Our broad literature search sought to capture any study that developed and/or validated a multivariable prediction model (i.e., at least two predictors) for a relevant clinical outcome for all non-organic psychiatric conditions (i.e., excluding neurodegenerative diseases such as dementia). This included studies where the outcome of interest was the current presence or future *onset* of a specific condition (e.g., categorical cut-offs based on established diagnostic criteria or validated psychometric screening tools), as well as any relevant future event or outcome related to the *course* of illness for a given psychiatric disorder (e.g., symptom remission or recurrence, poor psychosocial functioning, treatment response or resistance). Varying definitions and operationalisations of similar outcome domains were also permitted across reviewed studies.

All searches were performed as keyword searches. Four individual searches of the EMBASE (Ovid), MEDLINE (Ovid), PubMed, and PsycINFO (Ovid) databases were completed using the following search string:

(“risk prediction” OR “prediction model” OR “diagnostic model” OR “prognostic model” OR “predictive model” OR “risk calculator” OR “individualized risk” OR “machine learning”)

AND

(“substance use” OR “psychosis” OR “psychotic” OR “schizo*” OR “depressi*” OR “bipolar” OR “mania” OR “anxiety” OR “panic” OR “phobi*” OR “obsessive-compulsive” OR “post-traumatic” OR “eating disorder” OR “personality disorder” OR “autis*” OR “ADHD” OR “hyperkinetic” OR “conduct disorder” OR “oppositional”)

All searches were carried out (i) from inception to the fixed end-date of September 30, 2020, and further limited to studies that were (ii) published in English and (iii) classified as (Journal) Articles within the database.

**Appendix 2. Inclusion and exclusion criteria**

We selected English language, peer-reviewed articles up to September 30, 2020, including accepted articles available online pending print appearance. To reflect the methodological breadth of the published literature, both adult and paediatric populations were considered, with no restrictions regarding study setting (e.g., inpatient, outpatient, general population), design (e.g., cohort, case-control, randomised control studies) or timing (e.g., cross-sectional, retrospective, prospective). Although certain designs are preferable to minimise unwanted model bias, this was evaluated during quality assessment rather than applied as an exclusion criterion (see below). Of note however, for the subset of prognostic studies testing outcomes for course of illness, we only considered samples defined by specific psychiatric diagnoses, using diagnostic criteria or clinical symptoms, as opposed to more generalized operationalisations of ‘clinical’ samples (e.g., ‘history of mental illness’ or ‘current user of mental health services’). With most prediction models defined by allocation of individuals into discrete outcome-based groups, only studies reporting some estimate of classification-based predictive performance (e.g., discrimination, calibration) were included.

Abstracts, conference proceedings, correspondence, reviews, and meta-analyses were excluded, along with studies that: presented models with a single risk predictor; used inappropriate statistical methods (e.g., cluster analysis, principal components analysis) or an unspecified machine learning method; constructed a sample using meta-analysed individual patient data from across independent cohorts, or; sought to optimise the predictive value of an established psychometric or screening tool (e.g., shortening or sub-setting items).

We also excluded studies where all or most (i.e., >50%) candidate predictors were biomarkers or other biological indicators (e.g., neuroimaging, blood samples, genotyping). Where small numbers of biological variables were included among a larger pool of more readily available clinical or sociodemographic predictors, this was taken into account during quality assessment (see **Appendix 4**). In support of this decision, the specialised techniques required to collect and process these data, and the associated complexity and cost, may make the resulting models impractical for widespread clinical deployment [1]. Alongside pragmatic concerns, prediction studies based on neuroimaging or genetic data have, to date, been characterised by unrepresentative case-control designs and small sample sizes, particularly in relation to the large number of predictive features and use of complex machine learning approaches, all of which increases the risk of overestimated or unstable performance estimates [2,3]. For example, empirical evidence from neuroimaging-based machine learning studies for depression demonstrates deteriorating predictive performance as samples grow larger [4]. Different approaches to processing and analysing biological data across independent research teams can also introduce unwanted variations in statistical performance that may inhibit model reproducibility [5]. Recent reviews of published neuroimaging and genetic prediction studies in psychiatry include full discussions of these limitations [6,7]. However, we acknowledge that as methods for obtaining these data become more accessible, the pragmatic utility of these biological predictors is likely to improve.

Finally, regarding predictive models, recent lines of research have sought to support personalised or stratified medicine efforts by quantifying the relative advantage of one intervention over another for individual patients [8,9]. This is generally accomplished by comparing moderating and interaction effects for select pre-treatment characteristics across different treatment conditions. However, in their efforts to identify predictors of differential responding among several treatment options, these ‘treatment selection’ studies typically do not report the initial predictive ability observed within each individual treatment arm before comparing conditions using treatment modifiers. Consequently, unless information around the prediction of treatment response was available for a specific intervention, these studies were excluded from the current review.

**Appendix 3. Criteria for prediction model evaluation**

1. **Bias and overfitting**

Successful prediction models must minimise both *bias* (inclusion of systematic error) and o*verfitting* (where risk coefficients incorporate sample-specific error variance alongside the ‘true’ effect), both of which can produce inaccurate or inflated estimates of predictive accuracy [10]. Addressing these challenges requires sufficiently large sample sizes and, in particular, an adequate number of outcome events relative to the number of parameters estimated among candidate predictors. For binary outcomes, a minimum ‘events per variable’ (EPV) ratio of 10 has been widely adopted as a criterion to mitigate overfitting within development samples, with recent work recommending an EPV≥20 [11].

Naïve or inappropriate predictor selection strategies – particularly those based on stepwise methods or univariate associations with the outcome – can also bias risk coefficients and, in turn, performance estimates [12]. In particular, the need to capture comprehensive, multivariate models for psychiatric disorders should be balanced against a need to support practical implementation via parsimonious models with minimal redundant information. Therefore, current methodological guidelines generally advocate *a priori* predictor selection guided by existing meta-analytic knowledge or clinical relevance as the optimal approach, with simulation studies suggesting that resulting model performance may be comparable to more sophisticated machine-learning (ML) methods [13]. For regression-based models, penalisation and shrinkage techniques that reduce variance in parameter estimates, and can support automated variable selection, have also been recommended to address bias and overfitting, although these methods may still yield unreliable performance where sample sizes or EPV are insufficient [14].

1. **Generalizability**

As a result of bias and overfitting (see above), a model’s apparent discrimination or calibration in an initial (development) sample invariably over-estimates its predictive ability in the wider population, such that its performance is unlikely to be replicated in an independent (validation) sample. To determine *generalizability*, or the model’s ability to produce consistent and reliable predictions in new individuals, various internal validation techniques are available, which simulate the process of independent validation within the development sample itself by resampling (e.g., bootstrapping, cross-validation) or, sub-optimally, randomly splitting the data [12,15].

Internal validation, however sophisticated, is still no substitute for external validation in wholly-independent data (e.g., new site/country, later time period), which should involve adequately-powered external samples (>100 participants with outcome) [16] and appropriate statistical adjustments to correct for between-sample differences in case-mix and predictor distributions [17]. ‘Internal-external’ validation is also recommended if data from several sites are available to demonstrate the prediction model’s external validity across each site, while also using all available data to develop the final model [18].

1. **Clinical utility**

Finally, even when a prediction model has demonstrated generalizability, with consistent predictive performance across several independent settings, its real-world *clinical utility* can only be evidenced by an ability to enhance actionable decision-making. This is typically evaluated using statistical approximations of its ‘net benefit’ over current practice or, ideally, randomized impact studies quantifying tangible changes in patient outcomes following model implementation [12,19].

**Appendix 4. Data extraction**

Extraction of key study and model characteristics to summarise and critically appraise this literature was informed by the CHecklist for critical Appraisal and data extraction for systematic Reviews of prediction Modelling Studies (CHARMS) [20]. For studies that reported several models – for example, where individual risk estimates were generated for several unique outcomes, or where all analyses were stratified based on key subsamples (e.g., sex, treatment group) – we extracted information for each individual model. These details are tabulated in **Supplementary Table S2** under three main domains:

**Study information**

- *Author(s) and publication year*
- *Country*: Geographical location(s) of study participants (as opposed to the primary institution of authors or research team). For studies consolidating data from multiple countries, each individual country is listed.
- *Study setting*: Brief description of included participants and study design (e.g., adult vs paediatric; general-population vs clinical) along with any relevant selection criteria used to define the analytic sample (i.e., diagnosis, treatment condition). We also extracted or derived the mean age of each development sample, which is presented in brackets. Where this was unavailable, the overall age range is reported.
- *Outcome*: Description of outcome definition and timing. For studies estimating several models, each individual model’s outcome is specified. All outcomes are also categorised based on the wider psychiatric domain being considered, presented in brackets. These categories are as follows (in alphabetical order): ‘ADHD (attention-deficit/hyperactivity disorder)’; ‘Anxiety’; ‘ASD (autism spectrum disorder)’; ‘Bipolar’; ‘BPD (borderline personality disorder)’; ‘Depression’; ‘Eating Disorder’; ‘Mixed Affective’ (i.e., where a combination of anxiety, depressive, and/or other mood symptoms were consolidated in a single variable); ‘OCD (obsessive-compulsive disorder)’; ‘ODD (oppositional defiant disorder)’; ‘Psychosis’; ‘PTSD (post-traumatic stress disorder)’; ‘SUDs (substance use disorders)’; and ‘Transdiagnostic’ (i.e., where an outcome comprised several distinct diagnoses across externalizing and/or internalizing spectra).

**Model characteristics**

- *Model type*: Classification of each model based on an established taxonomy of diagnostic, prognostic, or predictive studies [21]. In brief, diagnostic models estimate risk for the current presence of a condition or illness-related event, typically based on cross-sectional or retrospective designs. Prognostic models, meanwhile, seek to predict future occurrence of a specific condition or event using prospective data. Finally, predictive models examine treatment-dependent outcomes (i.e., where treatment affects the outcome in one subgroup, or more in one subgroup than another); this distinguishes them from prognostic models, where the outcome reflects course of illness irrespective of treatment [22,23]. More specifically, if a model is developed in a single-arm trial without a comparison group, it is impossible to determine whether the identified predictors confer a different effect for treated vs non-treated individuals, or merely represent prognostic factors that would provide similar information about the outcome for those who receive no intervention [24]. Therefore, only treatment studies that included an appropriate comparison group (e.g., placebo or control condition) could be considered predictive, while any models that sought to predict treatment response among individuals all receiving the same intervention were classed as prognostic. Informed by these criteria, each prediction model was ultimately categorised as one of the following: ‘Diagnostic’; ‘Prognostic’ (with additional ‘onset’ or ‘course’ specifiers); or ‘Predictive’.
- *Sample size (number of events)*: To better capture the outcome incidence rate, we extracted the size of the development cohort used to estimate the final prediction model (i.e., following missing data handling, and ignoring hold-out internal validation samples). The number of events, or participants with the outcome, is presented in brackets. Where the event incidence was reported as a proportion of the total sample, this was used to approximate the actual number of events.
- *Events Per Variable (EPV)*: The ratio of the number of individuals with the outcome relative to the number of ‘predictor parameters’ among candidate predictors, including interactions and polynomials. Therefore, categorical predictors with *k* levels were counted as *k*–1 predictors here, in line with statistical conventions. As no reviewed study explicitly reported these estimates, we calculated approximate EPV values for all models where sufficient information was available, dividing the reported number of events by the reported or approximate number of candidate predictors – that is, predictors considered for inclusion in the model prior to any selection procedures. We note here that systematic criteria for calculating the minimum sample size for a prospective prediction model have recently been published to reduce reliance on a blanket ‘rule of thumb’ around sufficient EPV [25]. However, as these pre-dated the vast majority of reviewed studies, engagement with this method was not used as a quality criterion here.
- *Predictor selection*: List of all stated methods used to select an initial set of candidate predictors and/or the final predictor set, where sufficiently described.
- *Prediction modelling method*: Type of statistical analysis used to estimate model, ranging from traditional regression-based approaches (e.g., logistic or Cox regression) to unsupervised machine learning techniques (e.g., classification or decision trees, random forest, support vector machines, neural networks). For studies estimating and comparing several alternative methods to predict the same outcome, we list each approach and present the ‘best-performing’ method in italics – this was determined based on the authors’ own conclusions or, where no judgement was made, the model that yielded the best performance estimates (i.e., *c*-index/AUC). We note here that this procedure is distinct from ‘ensemble’ methods, where models generated by several distinct approaches are combined in a single omnibus model to optimise performance.

**Model performance and validation**

- *Internal validation*: Any stated method(s) of accounting or correcting for overfitting or optimism within the model development sample. To facilitate high-level comparison, internal validation methods were grouped into four categories, in descending order of preference within the current statistical literature: ‘Bootstrapping’; ‘Cross-validation’, ‘Non-random split sample’; ‘Random split-sample’.

In some cases, these methods were utilised without adhering to all of the necessary steps needed to ensure appropriate internal validation, in what has been termed ‘*pseudo* internal validation’ [26]. In particular, split-sample validation has consistently been shown to be an inefficient method of internal validation [15]. Although bootstrapping and cross-validation are preferred, these analyses should also encompass all stages of model development [27]. Studies that simply cross-validate or bootstrap a model to which variable selection has already been applied can inflate predictive accuracy estimates by introducing feature selection bias or other forms of unwanted circularity via ‘double dipping’, particularly where selection procedures in the full dataset are already based on associations with the outcome variable [28-30]. Therefore, studies combining feature selection or hyperparameter tuning with internal validation should ideally take place within a nested cross-validation or ‘double bootstrap’ framework, where an inner loop selects the optimal features before an outer loop tests whether that configuration generalizes to independent cases [31,32]. Similarly, use of bootstrapping or cross-validation solely for feature selection does not constitute internal validation, as likely performance in a new dataset is not evaluated. Guided by these statistical conventions, each model that utilised one of the above internal validation methods was rated based on whether they did [Y] or did not [N] meet criteria for sufficient internal validation (models were also rated ‘N’ where internal validation standards were unclear based on the detail provided).

- *Discrimination*: In the first instance, each model’s ability to distinguish individual cases with and without the outcome of interest was quantified using the concordance (*c*) index [33], which, for binary outcomes, is equivalent to the area under the curve (AUC) of the receiver operating characteristic (ROC) curve [34]. The *c*-index or AUC describes the probability that a randomly selected individual with the outcome will be assigned a higher risk probability than a random individual without the outcome. Values range from 0.5 (i.e., no discrimination [chance-level]) to 1 (perfect discrimination), with values of ≥0.7 and ≥0.8 generally denoting ‘acceptable’ and ‘good’ discrimination, respectively [35]. Where the *c*-index or AUC was not reported, particularly where the model tested a predefined or *a priori* risk threshold for the outcome, we extracted any key metrics of classification ability (e.g., sensitivity, specificity, accuracy, predictive values). Of note, where both ‘apparent’ and internally validated discrimination estimates were available, we extracted the internally validated estimate, given that it was more likely to reflect the model’s true predictive ability. Additionally, for studies comparing several modelling techniques (see *Prediction modelling method* above), the highest reported *c*-index/AUC was extracted.
- *Calibration*: Description of method(s) used to evaluate overall agreement between a model’s predicted risks and observed relative outcome frequencies. The optimal approach is to visualise calibration by plotting the two against each other, with perfect calibration denoted by a 45° line, or an intercept (or ‘calibration-in-the-large’) of 1 and slope of 0. Previously, calibration was frequently assessed using the Hosmer-Lemeshow goodness-of-fit test; however, following emerging concerns around whether this test was sufficiently powered to reject poor calibration, and the fact that it does not indicate the direction or magnitude of miscalibration, its use is no longer recommended [36]. However, as many studies pre-date this change in practice, we included the Hosmer-Lemeshow test as a viable measure of calibration. Finally, the unreliability (*U*) statistic, which uses a chi-square test to determine whether a model’s intercept and slope significantly diverge from the ‘ideal’ line, has recently emerged as an alternative calibration test [32]. In summary, calibration efforts were described using the following response options, in descending order based on preference: ‘Calibration plot’; ‘Calibration statistics (i.e., intercept, slope)’; ‘*U*-statistic’; ‘Hosmer-Lemeshow’; and ‘Other’ (with brief description).
- *External validation*: The optimal test of predictive performance is to apply the model to wholly independent data, such as individuals from a different location (geographical validation) or time-point (temporal validation). Where external validity was tested, we summarise validation sample characteristics (i.e., setting, sample size) and report key performance metrics. Of note, eight of the 228 reviewed studies attempted external validation of a previously published prediction model. To maintain continuity between a model’s development and validation across separate publications, these studies are summarised and cited within the entry for the original development study, and also listed as separate entries at the end of **Supplementary Table S2**. Conversely, studies that sought to update a pre-existing model by evaluating the incremental value offered by an additional predictor or interaction term, and did not attempt to directly replicate the original model, were treated as unique model development studies.

**Appendix 5. Quality assessment**

For each individual development and validation analysis reported within an eligible study, risk of bias (ROB), which indicates the likelihood of systematic distortions in performance due to shortcomings in study design, was evaluated using the Prediction model Risk Of Bias ASsessment Tool (PROBAST) [36,37]. In brief, we first specified the systematic review question (see *Introduction* in main manuscript), before classifying each study as (i) development only; (ii) development and validation, or; (iii) validation only. ROB was then assessed using 20 signalling questions across four domains (participants, predictors, outcome, analysis), with possible responses of ‘yes’, ‘probably yes’, ‘probably no’, ‘no’, or ‘no information’ Once all constituent items were reviewed, overall ROB for each overarching domain was rated as ‘high’, ‘low’ or ‘unclear’ based on a ‘worst score counts’ principle – that is, if one item in a domain indicates high ROB (i.e., the response is ‘no’ or ‘probably no’), then the overall domain is ascribed that classification. Similarly, a model only receives an overall ‘low ROB’ rating when all four assessment domains are judged to be low-risk (i.e., all items rated ‘yes’ or ‘probably yes’), while a high ROB classification is attributed to the entire model if any one domain is deemed high-risk. Overall domain ratings for model development and validation analyses are presented separately in **Supplementary Table S3**.

We quantified agreement among individual PROBAST ratings (*n* = 378) using a random subset of reviewed analyses (20.6%; *n* = 78) completed by two independent reviewers (AJM and SJL). Inter-rater reliability was interpreted using Cohen’s weighted kappa (*κ*) statistic based on the following guidelines: 0–0.20 (none to slight); 0.21–0.40 (fair); 0.41–0.60 (moderate); 0.61–0.80 (substantial); and 0.80–1 (almost perfect) [38].

The individual PROBAST items within each domain are as follows:

**Participants**

- 1. Were appropriate data sources used, e.g., cohort, RCT, or nested case-control study data?
  2. Were all inclusions and exclusions of participants appropriate?

**Predictors**

1. Were predictors defined and assessed in a similar way for all participants?
2. Were predictor assessments made without knowledge of outcome data?
3. Are all predictors available at the time the model is intended to be used?

**Outcomes**

1. Was the outcome determined appropriately?
2. Was a pre-specified or standard outcome definition used?
3. Were predictors excluded from the outcome definition?
4. Was the outcome defined and determined in a similar way for all participants?
5. Was the outcome determined without knowledge of predictor information?
6. Was the time interval between predictor assessment and outcome determination appropriate?

**Analysis**

1. Were there a reasonable number of participants with the outcome?
2. Were continuous and categorical predictors handled appropriately?
3. Were all enrolled participants included in the analysis?
4. Were participants with missing data handled appropriately?
5. Was selection of predictors based on univariable analysis avoided?

(*not applicable to validation analyses*)

1. Were complexities in the data (e.g., censoring, competing risks, sampling of control participants) accounted for appropriately?
2. Were relevant model performance measures evaluated appropriately?
3. Were model overfitting, underfitting, and optimism in model performance accounted for?

(*not applicable to validation analyses*)

1. Do predictors and their assigned weights in the final model correspond to the results from the reported multivariable analysis?

(*not applicable to validation analyses*)

**Supplementary Table S1.** PRISMA checklist

| **Section and Topic** | **Item #** | **Checklist item** | **Location where item reported** |
| --- | --- | --- | --- |
| **TITLE** | | |  |
| Title | 1 | Identify the report as a systematic review. | Title (p1) |
| **ABSTRACT** | | |  |
| Abstract | 2 | See the PRISMA 2020 for Abstracts checklist. | Abstract (p2) |
| **INTRODUCTION** | | |  |
| Rationale | 3 | Describe the rationale for the review in the context of existing knowledge. | Intro (p3) |
| Objectives | 4 | Provide an explicit statement of the objective(s) or question(s) the review addresses. | Intro (p3-4) |
| **METHODS** | | |  |
| Eligibility criteria | 5 | Specify the inclusion and exclusion criteria for the review and how studies were grouped for the syntheses. | Methods (p4-5); Appendix 2 |
| Information sources | 6 | Specify all databases, registers, websites, organisations, reference lists and other sources searched or consulted to identify studies. Specify the date when each source was last searched or consulted. | Methods (p4); Appendix 2 |
| Search strategy | 7 | Present the full search strategies for all databases, registers and websites, including any filters and limits used. | Appendix 2 |
| Selection process | 8 | Specify the methods used to decide whether a study met the inclusion criteria of the review, including how many reviewers screened each record and each report retrieved, whether they worked independently, and if applicable, details of automation tools used in the process. | Methods (p4-5); Appendix 3 |
| Data collection process | 9 | Specify the methods used to collect data from reports, including how many reviewers collected data from each report, whether they worked independently, any processes for obtaining or confirming data from study investigators, and if applicable, details of automation tools used in the process. | Methods (p5); Appendix 3 |
| Data items | 10a | List and define all outcomes for which data were sought. Specify whether all results that were compatible with each outcome domain in each study were sought (e.g. for all measures, time points, analyses), and if not, the methods used to decide which results to collect. | Appendix 4 |
|  | 10b | List and define all other variables for which data were sought (e.g. participant and intervention characteristics, funding sources). Describe any assumptions made about any missing or unclear information. | Appendix 4 |
| Study risk of bias assessment | 11 | Specify the methods used to assess risk of bias in the included studies, including details of the tool(s) used, how many reviewers assessed each study and whether they worked independently, and if applicable, details of automation tools used in the process. | Methods (p6); Appendix 5 |
| Effect measures | 12 | Specify for each outcome the effect measure(s) (e.g. risk ratio, mean difference) used in the synthesis or presentation of results. | Methods (p6) |
| Synthesis methods | 13a | Describe the processes used to decide which studies were eligible for each synthesis (e.g. tabulating the study intervention characteristics and comparing against the planned groups for each synthesis (item #5)). | Appendix 2 |
|  | 13b | Describe any methods required to prepare the data for presentation or synthesis, such as handling of missing summary statistics, or data conversions. | Methods (p6); Appendix 4 |
|  | 13c | Describe any methods used to tabulate or visually display results of individual studies and syntheses. | Results (p6-10) |
|  | 13d | Describe any methods used to synthesize results and provide a rationale for the choice(s). If meta-analysis was performed, describe the model(s), method(s) to identify the presence and extent of statistical heterogeneity, and software package(s) used. | Appendix 4 |
|  | 13e | Describe any methods used to explore possible causes of heterogeneity among study results (e.g. subgroup analysis, meta-regression). | n/a |
|  | 13f | Describe any sensitivity analyses conducted to assess robustness of the synthesized results. | Results (p6) |
| Reporting bias assessment | 14 | Describe any methods used to assess risk of bias due to missing results in a synthesis (arising from reporting biases). | Appendix 5 |
| Certainty assessment | 15 | Describe any methods used to assess certainty (or confidence) in the body of evidence for an outcome. | n/a |
| **RESULTS** | | |  |
| Study selection | 16a | Describe the results of the search and selection process, from the number of records identified in the search to the number of studies included in the review, ideally using a flow diagram. | Fig. 1 |
|  | 16b | Cite studies that might appear to meet the inclusion criteria, but which were excluded, and explain why they were excluded. | Appendix 2 |
| Study characteristics | 17 | Cite each included study and present its characteristics. | Table S2 |
| Risk of bias in studies | 18 | Present assessments of risk of bias for each included study. | Table S3 |
| Results of individual studies | 19 | For all outcomes, present, for each study: (a) summary statistics for each group (where appropriate) and (b) an effect estimate and its precision (e.g. confidence/credible interval), ideally using structured tables or plots. | Fig. 3; Table S2 |
| Results of syntheses | 20a | For each synthesis, briefly summarise the characteristics and risk of bias among contributing studies. | Results (p6); Fig. 4 |
|  | 20b | Present results of all statistical syntheses conducted. If meta-analysis was done, present for each the summary estimate and its precision (e.g. confidence/credible interval) and measures of statistical heterogeneity. If comparing groups, describe the direction of the effect. | Results (p6-10) |
|  | 20c | Present results of all investigations of possible causes of heterogeneity among study results. | Results (p6-10) |
|  | 20d | Present results of all sensitivity analyses conducted to assess the robustness of the synthesized results. | n/a |
| Reporting biases | 21 | Present assessments of risk of bias due to missing results (arising from reporting biases) for each synthesis assessed. | n/a |
| Certainty of evidence | 22 | Present assessments of certainty (or confidence) in the body of evidence for each outcome assessed. | n/a |
| **DISCUSSION** | | |  |
| Discussion | 23a | Provide a general interpretation of the results in the context of other evidence. | Discuss. (p10-15) |
|  | 23b | Discuss any limitations of the evidence included in the review. | Discuss. (p14) |
|  | 23c | Discuss any limitations of the review processes used. | Discuss. (p14) |
|  | 23d | Discuss implications of the results for practice, policy, and future research. | Discuss. (p10-15) |
| **OTHER INFORMATION** | | |  |
| Registration and protocol | 24a | Provide registration information for the review, including register name and registration number, or state that the review was not registered. | Abstract (p2); Methods (p6) |
|  | 24b | Indicate where the review protocol can be accessed, or state that a protocol was not prepared. | Methods (p6) |
|  | 24c | Describe and explain any amendments to information provided at registration or in the protocol. | n/a |
| Support | 25 | Describe sources of financial or non-financial support for the review, and the role of the funders or sponsors in the review. | Acknowledgments |
| Competing interests | 26 | Declare any competing interests of review authors. | Author Info |
| Availability of data, code and other materials | 27 | Report which of the following are publicly available and where they can be found: template data collection forms; data extracted from included studies; data used for all analyses; analytic code; any other materials used in the review. | Appendix 1-5 |

Checklist obtained from <http://prisma-statement.org/PRISMAStatement/Checklist>. n/a = not applicable. Page numbers based on accepted manuscript file. For detailed information on derivation and scoring, see elaboration by Page et al. [39]

**Supplementary Table S2.** Characteristics of included prediction modelling studies

| **Author (Year)** | **Country** | **Study setting (mean age or age range, years)** | **Outcome (Psychiatric domain)** | **Model type** | **Sample size**  **(*N* events)** | **EPV^1^** | **Predictor selection** | **Prediction modelling method^2^** | **Internal validation**  **[Valid: Y/N]** | **Discrimination^3^** | **Calibration** | **External validation** |
| --- | --- | --- | --- | --- | --- | --- | --- | --- | --- | --- | --- | --- |
| Abdullah-Koolmees et al (2018) [40] | Netherlands | Adult patients with psychotic or bipolar I disorder treated with antipsychotics upon hospital discharge (38.4) | Psychiatric rehospitalisation within 6 months (Psychosis)^4^ | Prognostic (course) | 87 (29) | 0.67 | Univariable analyses; backward selection | Cox proportional hazards regression | – | *c* = 0.82 | ­– | – |
| Acion et al (2017) [41] | USA | Hispanic outpatients undergoing treatment for substance use disorders (n.a.) | Successful treatment discharge (SUDs) | Prognostic (course) | 99,013 (44,748) | 113.86 | Inbuilt variable selection (various) | *Ensemble (Super Learner)*; Random forest; LASSO, elastic net and ridge logistic regression; Artificial neural network | Random split sample [N] | AUC = 0.82 | – | – |
| Addington et al (2010) [42] | Canada | Adult patients within first-episode psychosis programme (27) | Hospital admission within 1 year of enrolment (Psychosis) | Prognostic (course) | 297 (69) | 4.06 | Theory-driven (previous research; clinical expertise) | Logistic regression | – | *c* = 0.72 | – | Temporal validation using earlier admissions wave (*n* = 277; *c* = 0.72) |
|  |  | “ | Hospital admission within 2 years of enrolment (Psychosis) | Prognostic (course) | 297 (96) | 5.65 | “ | “ | – | *c* = 0.73 | – | Temporal validation using earlier admissions wave (*n* = 277; *c* = 0.68) |
|  |  | “ | Hospital admission within 3 years of enrolment (Psychosis) | Prognostic (course) | 297 (106) | 6.24 | “ | “ | – | *c* = 0.74 | – | Temporal validation using earlier admissions wave (*n* = 277; *c* = 0.67) |
| Addington et al (2017) [43] | USA | CHR subjects within longitudinal study (19.8) | Conversion to psychosis at follow-up (Psychosis) | Prognostic (onset) | 145 (29) | 1.45 | Theory-driven (previous research); inbuilt variable selection (LASSO) | LASSO cox regression | Cross-validation [N] | *c* = 0.73 | – | – |
| Agne et al (2020) [44] | Brazil | Outpatients with OCD (35) | Reported lifetime suicide attempt (OCD) | Diagnostic | 959 (104) | 0.49 | Theory-driven (previous research); inbuilt variable selection (elastic net) | Elastic net logistic regression | Random split sample [N] | AUC = 0.95 | – | – |
| Ahn et al (2016) [45] | USA | Cross-sectional sample of cocaine dependent individuals and healthy controls (41.2) | Current cocaine dependence classification (SUDs) | Diagnostic | 54 (31) | 2.07 | Inbuilt variable selection (LASSO) | LASSO logistic regression | Random split sample [N] | AUC = 0.91 | – | – |
| Ahn and Vassileva (2016) [46] | Bulgaria | Adult drug users within larger study of impulsivity (25.8) | Current heroin dependence classification (SUDs) | Diagnostic | 222 (70) | 1.30 | Inbuilt variable selection (elastic net) | Elastic net logistic regression | Random split sample, repeated [N] | AUC = 0.86 | – | – |
|  |  | “ | Current amphetamine dependence classification (SUDs) | Diagnostic | 222 (79) | 1.46 | “ | “ | “ | AUC = 0.71 | – | – |
| **Author (Year)** | **Country** | **Study setting (mean age or age range, years)** | **Outcome (Psychiatric domain)** | **Model type** | **Sample size**  **(*N* events)** | **EPV^1^** | **Predictor selection** | **Prediction modelling method^2^** | **Internal validation**  **[Valid: Y/N]** | **Discrimination^3^** | **Calibration** | **External validation** |
| Amminger et al (2015) [47] | Austria | CHR patients within double-blind RCT who received long-chain omega-3 fatty acids  (16.9) | ≥15 point increase in global functioning at end of 12-week trial (Psychosis) | Prognostic (course) | 40 (22) | 1.69 | Univariable analyses | Gaussian process classification | Cross-validation [Y] | Acc. = 0.87  Sen. = 0.87  Spe. = 0.87 | – | – |
|  |  | CHR patients within double-blind RCT who received placebo  (16.1) | ≥15 point increase in global functioning at end of 12-week trial (Psychosis) | Prognostic (course) | 40 (12) | 0.92 | “ | “ | “ | Acc. = 0.79  Sen. = 0.83  Spe. = 0.75 | – | – |
| Andrews et al (2017) [48] | UK | Older adults within retrospective study (n.a.) | Depression status at 10-week follow-up (Depression) | Prognostic (onset) | 37 (n.a.) | – | Inbuilt variable selection (LASSO) | LASSO logistic regression | Cross-validation [N] | *c* = 0.88 | – | – |
| Askland et al (2015) [49] | USA | Adults with primary OCD diagnosis within longitudinal study  (40.4) | At least one period of remittance (≥8 weeks) over follow-up up to 12 years (OCD) | Prognostic (course) | 296 (158) | 0.2 | Inbuilt variable selection  (variable importance) | Random forest | – | Acc. = 0.76 | – | – |
| Bares et al (2017) [50] | Czech Republic | Hospitalized patients with major depressive disorder treated with antidepressants (45.5) | Treatment response at 6-week follow-up (Depression) | Prognostic (course) | 38 (21) | 3.5 | Univariable analyses | Logistic regression | – | *c* = 0.92 | – | Geographic validation using independent dataset (*n* = 87; adjusted *R*^2^ = 0.74) |
| Barnes et al (2019 )[51] | USA | Veterans with PTSD and elevated suicide risk in psychiatric unit (45) | Suicide attempt during 6-month follow-up (PTSD) | Prognostic (course) | 92 (17) | 1.06 | Univariable analyses; backward selection | Logistic regression | – | *c* = 0.87 | – | – |
| Barros et al (2017) [52] | Chile | Individuals seeking inpatient or outpatient treatment for mood disorders at three health centres (39.7) | Current presence of suicidal behaviour (Mixed Affective) | Diagnostic | 707 (349) | 1.56 | Univariable analyses; correlation-based method | Classification and regression trees; K-nearest neighbour; Random forest; AdaBoost; *Support vector machine* | ­Cross-validation [N] | Acc. = 0.78  Sen. = 0.77  Spe. = 0.79 | – | – |
| Bauer et al (2019) [53] | USA | Children and adolescents with bipolar disorder vs healthy controls (12.3) | Bipolar disorder classification (Bipolar) | Diagnostic | 168 (119) | 3.61 | Inbuilt variable selection | Gradient boosting | Cross-validation [N] | AUC = 0.90 | – | – |
|  |  | Children and adolescents with bipolar disorder vs healthy controls and unaffected offspring (11.3) | Bipolar disorder classification (Bipolar) | Diagnostic | 227 (119) | 3.61 | “ | “ | “ | AUC = 0.80 | – | – |
| Bellón et al (2011) [54] | Spain | Prospective cohort of adult primary care patients without depression at baseline (49.2) | Onset of major depression at 12-month follow-up (Depression) | Prognostic (onset) | 2,787 (321) | 8.23 | Multivariate significance | Logistic regression with shrinkage factor | Random split sample [N] | *c* = 0.82 | Calibration plot | Geographic validation using samples from Chile (*n* = 1,844; *c* = 0.70), Estonia (*n*= 823; *c* = 0.73), Netherlands (*n* = 731; *c* = 0.83), Portugal (*n* = 844; *c* = 0.71), Slovenia (*n* = 866; *c* = 0.82), and the UK (*n* = 811; *c* = 0.76) |
| **Author (Year)** | **Country** | **Study setting (mean age or age range, years)** | **Outcome (Psychiatric domain)** | **Model type** | **Sample size**  **(*N* events)** | **EPV^1^** | **Predictor selection** | **Prediction modelling method^2^** | **Internal validation**  **[Valid: Y/N]** | **Discrimination^3^** | **Calibration** | **External validation** |
| Birmaher et al (2018) [55] | USA | Longitudinal study of youth with bipolar disorder not otherwise specified (11.9) | Conversion to bipolar disorder I or II during 5-year follow-up (Bipolar) | Prognostic (onset) | 140 (57) | 5.18 | Theory-driven (previous research) | Cox proportional hazards regression | Bootstrapping [Y] | AUC = 0.71 | Calibration plot; Hosmer-Lemeshow | Geographic validation using dataset of youth with bipolar disorder not otherwise specified (*n*=58; AUC=0.75) |
| Birmaher et al (2020) [56] | USA | Longitudinal study of youth with bipolar disorder (24.5) | Recurrence of any mood, depressive or hypo/manic episode during 5-year follow-up (Bipolar) | Prognostic (course) | 363 (294) | 8.4 | Theory-driven (previous research) | Classification trees | Random split sample [N] | AUC = 0.82 | Calibration plot; Hosmer-Lemeshow | – |
| Birnbaum et al (2019) [57] | USA | Inpatients and outpatients with recent-onset primary psychotic disorder (24) | Relapse rehospitalisation within one month (Psychosis) | Prognostic (course) | 51 (n.a.) | – | Variable selection based on coefficient of variance | Support vector machine | Random split sample [N] | Sen. = 0.38  Spe. = 0.71  PPV = 0.66  NPV = 0.44 | – | – |
| Birnbaum et al (2020) [58] | USA | Inpatients and outpatients with schizophrenia spectrum disorder and healthy volunteers (24.4) | Classification of individuals with schizophrenia spectrum disorder (Psychosis) | Diagnostic | 116 (42) | 0.34 | Inbuilt variable selection (feature importance) | *Random forest;* Support vector machine; Gradient boosting | Cross-validation [N] | AUC = 0.74 | – | – |
|  |  | “ | Classification of periods of ‘relative health’ vs ‘relative illness’ (Psychosis) | Diagnostic | 116 (38) | 0.31 | “ | Random forest*;* *Support vector machine*; Gradient boosting | “ | AUC = 0.71 | – | – |
| Bishop-Fitzpatrick et al (2018) [59] | USA | Matched sample of decedents with ASD and community controls, drawn from electronic health records (68) | Correct classification of ASD decedents (ASD) | Diagnostic | 6,277 (91) | 0.01 | Inbuilt variable selection | Random forest | Cross-validation [N] | AUC = 0.88 | – | – |
| Bledsoe et al (2016) [60] | USA | Matched university sample of ADHD and control participants (n.a.) | Current ADHD classification (ADHD) | Diagnostic | 35 (23) | 1.52 | Forward selection | Support vector machine | Cross-validation [N] | Acc. = 0.97  Sen. = 1  Spe. = 0.96 | – | – |
| Boer et al (2019) [61] | Netherlands | Adult patients with primary depressive disorder in naturalistic cohort study (41.2) | Prolonged treatment course (≥2 years) (Depression) | Prognostic (course) | 716 (301) | 35.8 | Univariable analyses; backward selection | Logistic regression | Bootstrapping [N] | AUC = 0.65 | – | – |
|  |  | Adult patients with primary anxiety disorder in naturalistic cohort study (35.6) | Prolonged treatment course (≥2 years) (Anxiety) | Prognostic (course) | 509 (168) | 9.88 | “ | “ | “ | AUC = 0.68 | – | – |
| Bokma et al (2020) [62] | Netherlands | Patients with anxiety disorder at baseline within longitudinal cohort (41.9) | Recovery at 2-year follow-up (Anxiety) | Prognostic (course) | 887 (484) | 0.74 | *A priori* based on available data | Random forest | Cross-validation [N] | AUC = 0.67 | – | – |
| **Author (Year)** | **Country** | **Study setting (mean age or age range, years)** | **Outcome (Psychiatric domain)** | **Model type** | **Sample size**  **(*N* events)** | **EPV^1^** | **Predictor selection** | **Prediction modelling method^2^** | **Internal validation**  **[Valid: Y/N]** | **Discrimination^3^** | **Calibration** | **External validation** |
| Boscarino et al (2011) [63] | USA | New York residents interviewed 1 year after World Trade Center Disaster (43.1) | PTSD diagnosis at 1-year follow-up (PTSD) | Prognostic (onset) | 2,368 (174) | – | Stepwise selection | Logistic regression | Bootstrapping [N] | AUC = 0.94 | – | Geographic validation using independent chronic pain (*n* = 705; AUC = 0.97) and trauma centre patients (*n* = 225; AUC = 0.93) |
| Breen et al (2019) [64] | South Africa | Adult women who had experienced a single sexual assault with and without PTSD diagnosis (25.1) | Current PTSD classification (PTSD) | Diagnostic | 40 (20) | 0.27 | Inbuilt variable selection (recursive feature elimination) | Support vector machine | Cross-validation [Y] | AUC = 0.70 | – | ­– |
| Browning et al (2019) [65] | UK | Depressed primary care patients undergoing antidepressant treatment (41.1) | Clinical response (≥50% symptom reduction) at 4-6 week follow-up (Depression) | Prognostic (course) | 57 (22) | 0.34 | Inbuilt variable selection (ranking method) | Support vector machine | Cross-validation [N] | Acc. = 0.77  Sen. = 0.73  Spe. = 0.80  PPV = 0.70  NPV = 0.82 | – | Geographic validation using independent sample (*n* = 239; Acc. = 0.60; Sen. = 0.58; Spe. = 0.64; PPV = 0.74; NPV = 0.46) |
| Bussu et al (2018) [66] | UK | Infants classed as high-risk siblings or low-risk controls within longitudinal study (6.4) | ASD diagnosis among high-risk sibling group at 36 months (ASD) | Prognostic (onset) | 161 (32) | 1.6 | Available data | Support vector machine | Cross-validation [N] | AUC = 0.71 | – | – |
| Cannon et al (2008) [67] | USA | CHR patients within longitudinal study (18.1) | Conversion to psychosis during 2-year follow-up (Psychosis) | Prognostic (onset) | 291 (82) | 1.06 | Univariable analyses; backward selection | Cox proportional hazards regression | – | Sen. = 0.08  Spe. = 0.98  PPV = 0.79 | – | Geographic validation using independent sample of CHR patients (Thompson et al, 2011: *n* = 104) |
| Cannon et al (2016) [68] | USA | CHR patients within longitudinal study (18.5) | Conversion to psychosis during 2-year follow-up (Psychosis) | Prognostic (onset) | 596 (84) | 10.5 | Theory-driven (previous research; EPV) | Cox proportional hazards regression | Bootstrapping [Y] | *c* = 0.71 | Calibration plot | Geographic validation in two CHR samples (Carrión et al, 2016: *n* = 176; AUC = 0.79; Osborne et al, 2019: *n* = 62; AUC = 0.71) |
| Carrión et al (2013) [69] | USA | Treatment-seeking CHR patients (16) | Poor social functioning at 3-5 year follow-up (Psychosis) | Prognostic (course) | 92 (44) | 0.66 | Univariable analyses | Logistic regression | – | AUC = 0.82 | – | – |
|  |  | “ | Poor role functioning at 3-5 year follow-up (Psychosis) | Prognostic (course) | 92 (45) | 0.67 | “ | “ | – | AUC = 0.77 | – | – |
| Carter et al (2002) [70] | Denmark | ‘High-risk’ children of mothers with schizophrenia in longitudinal study (15.1) | Lifetime schizophrenia diagnosis at 25-year follow-up (Psychosis) | Prognostic (onset) | 293 (33) | 1.32 | Univariable analyses | Discriminant function analysis | – | Sen. = 0.67  Spe. = 0.76  PPV = 0.26  NPV = 0.95 | – | – |
| **Author (Year)** | **Country** | **Study setting (mean age or age range, years)** | **Outcome (Psychiatric domain)** | **Model type** | **Sample size**  **(*N* events)** | **EPV^1^** | **Predictor selection** | **Prediction modelling method^2^** | **Internal validation**  **[Valid: Y/N]** | **Discrimination^3^** | **Calibration** | **External validation** |
| Cattelani et al (2019) [71] | UK | Community-dwelling older adults within longitudinal cohort (60-75) | Clinical depressive symptoms at 2-year follow-up (Depression) | Prognostic (onset) | 7,920 (436) | 87.2 | Theory-driven (previous research) | Probabilistic model based on meta-analytic odds ratios | – | AUC = 0.76 | – | – |
|  | Italy | “ | Clinical depressive symptoms at 3-year follow-up (Depression) | Prognostic (onset) | 660 (113) | 22.6 | “ | “ | – | AUC = 0.74 | – | – |
|  | Ireland | “ | Clinical depressive symptoms at 2-year follow-up (Depression) | Prognostic (onset) | 3,124 (122) | 24.4 | “ | “ | – | AUC = 0.77 | – | – |
| Caye et al (2020) [72] | UK | Children within prospective cohort without childhood ADHD symptoms (17) | Adult-onset ADHD diagnosis (ADHD) | Prognostic (onset) | 5,113 (486) | 34.7 | Theory-driven (previous research) | *Logistic regression*; Random forest; Gradient boosting; Artificial neural network | Bootstrapping [Y] | AUC = 0.82 | Calibration plot | Geographic validation in external samples from the UK (*n* = 2,040; AUC = 0.75), Brazil (*n* = 4,039; AUC = 0.57) and USA (*n* = 717; AUC = 0.76) |
| Cearns et al (2019) [73] | Germany | Patients hospitalized for acute depressive episode (49.5) | Rehospitalisation during 2-year follow-up (Depression) | Prognostic (course) | 380 (102) | 0.49 | Inbuilt variable selection (elastic net) | Support vector machine | Cross-validation [Y] | AUC = 0.68 | – | – |
| Cepeda et al (2018) [74] | USA | Adults from three health claims databases prescribed with at least one antidepressant in past year (48) | Classification as treatment-resistant (Depression) | Prognostic (course) | 22,057 (2,076) | 207.6 | Ranking-based method | Decision tree analysis | Random split sample [N] | AUC = 0.81 | – | Geographic validation in three independent claims databases (*n* = 9,069; AUC = 0.79; *n* = 1,899; AUC = 0.78; *n* = 3,877; AUC = 0.78) |
| Chang et al (2006) [75] | Taiwan | Hospitalized patients with schizophrenia undergoing antipsychotic trial (34) | Symptom reduction at 4-week follow-up (Psychosis) | Prognostic (course) | 117 (60) | 5.46 | Multivariate significance | Logistic regression (generalized estimating equation) | – | AUC = 0.85 | – | – |
|  |  | “ | Symptom reduction at 6-week follow-up (Psychosis) | Prognostic (course) | 102 (58) | 5.27 | “ | “ | – | AUC = 0.86 | – | – |
| Chekroud et al (2016) [76] | USA | Depressed adult patients allocated to multiple antidepressant treatments within trial (18-75) | Symptom remission at 12-week follow-up (Depression) | Predictive | 1,949 (949) | 5.79 | Inbuilt variable selection (elastic net) | Gradient boosting machine | Cross-validation [N] | AUC = 0.70 | Calibration plot | Geographic validation in three treatment groups from independent clinical trial (*n* = 134-151; Acc. = 0.51-0.60; Sen. = 0.39-0.56, Spe. = 0.63-0.71) |
| Chekroud et al (2018) [77] | USA | Adults diagnosed with depression in past year (n.a.) | Failure to initiate mental health treatment despite identified need (Depression) | Diagnostic | 20,785 (6,271) | 73.8 | Theory-driven (previous research) | Extreme gradient boosting | Cross-validation [Y] | AUC = 0.79 | – | Temporal validation using subjects drawn from later cohort wave (*n* = 55,271; AUC = 0.78) |
| **Author (Year)** | **Country** | **Study setting (mean age or age range, years)** | **Outcome (Psychiatric domain)** | **Model type** | **Sample size**  **(*N* events)** | **EPV^1^** | **Predictor selection** | **Prediction modelling method^2^** | **Internal validation**  **[Valid: Y/N]** | **Discrimination^3^** | **Calibration** | **External validation** |
| Cho et al (2019) [78] | South Korea | Patients in prospective cohort with a major mood disorder (25.9) | Biased vs neutral mood state during 3-day follow-up (Mixed Affective) | Prognostic (course) | 55 (n.a.) | – | Inbuilt variable selection (variable importance) | Random forest | – | AUC = 0.70 | – | – |
|  |  | Patients in prospective cohort with major depressive disorder (20.6) | Biased vs neutral mood state during 3-day follow-up (Depression) | Prognostic (course) | 18 (n.a.) | – | “ | “ | – | AUC = 0.69 | – | – |
|  |  | Patients in prospective cohort with bipolar I disorder (20.3) | Biased vs neutral mood state during 3-day follow-up (Bipolar) | Prognostic (course) | 18 (n.a.) | – | “ | “ | – | AUC = 0.67 | – | – |
|  |  | Patients in prospective cohort with bipolar II disorder (21.2) | Biased vs neutral mood state during 3-day follow-up (Bipolar) | Prognostic (course) | 19 (n.a.) | – | “ | “ | – | AUC = 0.67 | – | – |
| Choi et al (2018) [79] | USA | Older adults within longitudinal study (n.a.) | Self-reported depression at 5-6-year follow-up (Depression) | Prognostic (onset) | 3,377 (n.a.) | – | Correlation-based feature selection | Logistic regression; Ridge logistic regression; *Random forest* | Cross-validation [N] | AUC = 0.81 | – | – |
| Chondros et al (2018) [80] | Australia | Adult primary care patients with current depressive symptoms (47.7) | Depressive symptoms at 3-month follow-up (Depression) | Prognostic (course) | 511 (n.a.) | – | Multivariate significance | Linear regression with heuristic shrinkage factor | – | *c* = 0.74 | Calibration plot | – |
| Christensen et al (2009) [81] | USA | Patients within RCT who had suffered intracerebral haemorrhage (63) | Depressed mood at 90-day follow-up (Depression) | Prognostic (onset) | 596 (120) | 5.46 | Univariable and multivariable analyses | Logistic regression | – | *c* = 0.73 | – | – |
| Chua et al (2019) [82] | Singapore | First-episode psychosis patients within longitudinal study (27.5) | Unemployment at 2-year follow-up (Psychosis) | Prognostic (course) | 1177 (348) | 12 | – | Logistic regression | – | AUC = 0.76 | – | – |
| Chung et al (2019) [83] | USA | CHR patients within longitudinal study (n.a.) | Conversion to psychosis during 2-year follow-up (Psychosis) | Prognostic (onset) | 476 (67) | 11.17 | Theory-driven (update of existing model)[68] | Cox proportional hazards regression | Bootstrapping [Y] | *c* = 0.70 | – | – |
| Ciarleglio et al (2019) [84] | USA | Help-seeking CHR individuals within early detection programme (20.1) | Conversion to psychosis during 2-year follow-up (Psychosis) | Prognostic (onset) | 199 (64) | 1.6 | Inbuilt variable selection (LASSO) | LASSO logistic regression | Bootstrapping [N] | *c* = 0.73 | – | – |
| Clark et al (2016) [85] | Austria | CHR subjects within placebo group of 12-week omega-3 trial (12.9-22.3) | Conversion to psychosis during 1-year follow-up (Psychosis) | Prognostic (onset) | 40 (11) | 0.55 | Univariable analyses | Logistic regression | Bootstrapping [N] | AUC = 0.92 | – | – |
| **Author (Year)** | **Country** | **Study setting (mean age or age range, years)** | **Outcome (Psychiatric domain)** | **Model type** | **Sample size**  **(*N* events)** | **EPV^1^** | **Predictor selection** | **Prediction modelling method^2^** | **Internal validation**  **[Valid: Y/N]** | **Discrimination^3^** | **Calibration** | **External validation** |
| Connor et al (2007) [86] | Australia | Alcohol dependent outpatients who completed abstinence-based programme of cognitive-behavioural therapy (n.a.) | Abstinence at 12-week follow-up (SUDs) | Prognostic (course) | 73 (n.a.) | – | Stepwise selection | Discriminant analysis; *Decision tree*; Bayesian network | Random split sample [N] | Acc. = 0.77 | – | – |
|  |  | Alcohol-dependent outpatients who completed abstinence-based cognitive-behavioural therapy and prescribed additional relapse-prevention agent (n.a.) | Abstinence at 12-week follow-up (SUDs) | Prognostic (course) | 66 (n.a.) | – | “ | Discriminant analysis; *Decision tree*; Bayesian network | “ | Acc. = 0.77 | – | – |
| Corcoran et al (2018) [87] | USA | CHR individuals (25.5) | Conversion to psychosis during 2-year follow-up (Psychosis) | Prognostic (onset) | 59 (19) | 1.36 | Factor analysis | Logistic regression | – | AUC = 0.87 | – | Geographic validation in CHR individuals at independent site (*n* = 34; AUC = 0.72) |
| Cornblatt et al (2015) [88] | USA | Treatment-seeking CHR adolescents (16) | Conversion to psychosis during 5-year follow-up (Psychosis) | Prognostic (onset) | 92 (15) | 0.22 | Univariable analyses; backward selection | Cox proportional hazards regression | Bootstrapping [N] | AUC = 0.92 | Hosmer-Lemeshow | – |
| Crippa et al (2017) [89] | Italy | Matched sample of ADHD-diagnosed children vs controls (11.5) | Current ADHD diagnosis (ADHD) | Diagnostic | 44 (22) | – | Inbuilt variable selection (wrapper approach) | Support vector machine | Cross-validation [Y] | AUC = 0.80 | – | – |
| Davidson et al (1999) [90] | Israel | Matched sample of male adolescents assessed for military service who later developed schizophrenia vs non-patients (n.a.) | Hospitalisation for schizophrenia during 4-10 year follow-up (Psychosis) | Prognostic (onset) | 9,724 (509) | 29.94 | – | Logistic regression | Random split sample [N] | Acc. = 0.88  Sen. = 0.75  Spe. = 0.97  PPV = 0.72 | – | – |
| de Man-van Ginkel et al (2013) [91] | Netherlands | Stroke patients from prospective multi-hospital study (68.2) | Major depression diagnosis 6-8 weeks after stroke (Depression) | Prognostic (onset) | 382 (54) | 1.8 | Backward selection | Logistic regression | Bootstrapping [N] | AUC = 0.78 | *U*-statistic | – |
| Delgadillo et al (2017) [92] | UK | Patients accessing low and/or high-intensity psychological therapy (42) | Post-treatment improvement in depressive symptoms (Depression) | Predictive | 592 (281) | 23.42 | Inbuilt variable selection (LASSO) | LASSO logistic regression | Random split sample [N] | AUC = 0.64 | – | – |
|  |  | “ | Post-treatment improvement in generalized anxiety symptoms (Anxiety) | Predictive | 644 (306) | 25.5 | “ | “ | “ | AUC = 0.70 | – | – |
| **Author (Year)** | **Country** | **Study setting (mean age or age range, years)** | **Outcome (Psychiatric domain)** | **Model type** | **Sample size**  **(*N* events)** | **EPV^1^** | **Predictor selection** | **Prediction modelling method^2^** | **Internal validation**  **[Valid: Y/N]** | **Discrimination^3^** | **Calibration** | **External validation** |
| Delgadillo et al (2020) [93] | UK | Clinical registry of patients accessing cognitive-behavioural therapy (39.2) | Clinically-significant symptom improvement (Depression) | Predictive | 929 (434) | 28.93 | Inbuilt variable selection (elastic net) | *Elastic net logistic regression*; Random forest | Random split sample [N] | AUC = 0.59 | – | ­– |
|  |  | Clinical registry of patients accessing person-centred counselling for depression (41.2) | Clinically-significant symptom improvement (Depression) | Predictive | 156 (143) | 9.53 | “ | “ | “ | AUC = 0.65 | – | – |
| Demetriou et al (2020) [94] | Australia | Clinical referrals with social anxiety disorder, early psychosis, or autism spectrum disorder vs neurotypical controls (22.7) | Classification of any of the three clinical disorders (Transdiagnostic) | Diagnostic | 236 (193) | 3.78 | Inbuilt variable selection | Random forest; Boruta; LASSO logistic regression; Elastic net logistic regression; *Bayesian additive regression trees* | Cross-validation [N] | AUC = 0.92 | – | – |
|  |  | Clinical referrals with social anxiety disorder vs early psychosis, or autism spectrum disorder (22.7) | Classification of social anxiety disorder group (Anxiety) | Diagnostic | 193 (83) | 1.63 | “ | “ | “ | AUC = 0.78 | – | – |
|  |  | Clinical referrals with autism spectrum disorder vs social anxiety disorder or early psychosis (22.7) | Classification of autism spectrum disorder group (ASD) | Diagnostic | 193 (62) | 1.22 | “ | “ | “ | AUC = 0.74 | – | – |
|  |  | Clinical referrals with early psychosis vs social anxiety disorder or autism spectrum disorder (22.7) | Classification of early psychosis group (Psychosis) | Diagnostic | 193 (48) | 0.94 | “ | “ | “ | AUC = 0.78 | – | – |
| Dennis et al (2004) [95] | Canada | Pregnant women within longitudinal study (28.5) | Depressive symptoms at 1-week postpartum (Depression) | Prognostic (onset) | 498 (144) | – | Univariable and multivariable analyses | Logistic regression | – | Sen. = 0.44  Spe. = 0.90 | – | – |
| Denys et al (2003) [96] | Netherlands | Outpatients with OCD receiving paroxetine or venlafaxine XR within RCT (35.3) | Treatment response following 12-week trial (OCD) | Predictive | 144 (61) | 4.07 | Theory-driven (previous research); univariable analyses; backward selection | Logistic regression | – | AUC = 0.71 | Hosmer-Lemeshow | – |
| Dinga et al (2018) [97] | Netherlands | Depressed patients within multi-centre study (42) | Presence of MDD diagnosis at 2-year follow-up (Depression) | Prognostic (course) | 804 (397) | 7.22 | Inbuilt variable selection (elastic net) | Elastic net logistic regression | Cross-validation [N] | AUC = 0.66 | – | – |
| Dipnall et al (2017) [98] | USA | Adults from cross-sectional community survey (42.6) | Moderate-to-severe depression (Depression) | Diagnostic | 5,546 (523) | 14.94 | Factor analysis | Generalized structural equation model | Random split sample [N] | AUC = 0.85 | Hosmer-Lemeshow | – |
| **Author (Year)** | **Country** | **Study setting (mean age or age range, years)** | **Outcome (Psychiatric domain)** | **Model type** | **Sample size**  **(*N* events)** | **EPV^1^** | **Predictor selection** | **Prediction modelling method^2^** | **Internal validation**  **[Valid: Y/N]** | **Discrimination^3^** | **Calibration** | **External validation** |
| Edgcomb et al (2019) [99] | USA | Hospitalized adult patients with comorbid bipolar disorder and medical illness (n.a.) | 30-day psychiatric readmission (Bipolar) | Prognostic (course) | 552 (47) | 0.19 | Ranking-based method | Classification and regression trees | Cross-validation [N] | AUC = 0.87 | – | – |
| Fazel et al (2017) [100] | Sweden | Inpatients and outpatients diagnosed with schizophrenia-spectrum or bipolar disorder (44) | Any violent offence within 1 year of patient episode (Psychosis/Bipolar) | Prognostic (course) | 58,771 (830) | 28.62 | Theory-driven (previous research); backward selection | Logistic regression | Bootstrapping [N] | *c* = 0.86 | Calibration plot | Geographic validation in subsample of select regions (*n* = 16,387; *c* = 0.89) and independent patient sample (Negatsch et al, 2019: *n* = 474; AUC = 0.72) |
| Fazel et al (2019) [101] | Sweden | Inpatients and outpatients diagnosed with schizophrenia-spectrum or bipolar disorder (44) | 1-year risk of suicide (Psychosis/Bipolar) | Prognostic (course) | 58,771 (494) | 11.23 | Theory-driven (previous research); backward selection | Logistic regression | Bootstrapping [N] | *c* = 0.75 | Calibration plot | Geographic validation in subsample of regions (*n* = 16,387; *c* = 0.71) |
| Flygare et al (2020) [102] | Sweden | Individuals with body dysmorphic disorder within randomised control trial (32.5) | Post-treatment (12-week) remission (Eating Disorders) | Prognostic (course) | 88 (27) | 0.55 | Correlation-based method; univariable analyses; inbuilt variable selection (feature importance) | *Random forest;* Logistic regression | Cross-validation [N] | AUC = 0.78 | – | – |
|  |  | “ | Remission at 3-month follow-up (Eating Disorders) | Prognostic (course) | 88 (37) | 0.76 | “ | “ | “ | AUC = 0.78 | – | – |
|  |  | “ | Remission at 12-month follow-up (Eating Disorders) | Prognostic (course) | 88 (41) | 0.84 | “ | “ | “ | AUC = 0.73 | – | – |
|  |  | “ | Remission at 24-month follow-up (Eating Disorders) | Prognostic (course) | 88 (53) | 1.08 | “ | “ | “ | AUC = 0.64 | – | – |
| Fond et al (2019) [103] | France | Patients with schizophrenia or schizoaffective disorder (32.7) | Psychotic relapse during 2-year follow-up (Psychosis) | Prognostic (course) | 315 (125) | 1.6 | Inbuilt variable selection | Classification and regression trees | Random split sample [N] | Acc. = 0.64  Sen. = 0.71  Spe. = 0.45 | – | – |
| Francesconi et al (2017) [104] | Italy | Non-psychotic outpatients (24.3) | Conversion to psychosis during 36-month follow-up (Psychosis) | Prognostic (onset) | 138 (21) | 0.34 | Univariable analyses; backward selection | Cox proportional hazards regression | Bootstrapping [N] | AUC = 0.88 | Hosmer-Lemeshow | – |
| **Author (Year)** | **Country** | **Study setting (mean age or age range, years)** | **Outcome (Psychiatric domain)** | **Model type** | **Sample size**  **(*N* events)** | **EPV^1^** | **Predictor selection** | **Prediction modelling method^2^** | **Internal validation**  **[Valid: Y/N]** | **Discrimination^3^** | **Calibration** | **External validation** |
| Furukawa et al (2019) [105] | Japan | Adults with unipolar major depression within multi-centre randomised trial (43) | Remittance of depressive symptoms at 9-week follow-up, using data from Week 1 (Depression) | Predictive | 1927 (717) | 51.21 | Backward selection | Logistic regression | – | AUC = 0.75 | Hosmer-Lemeshow | Temporal validation based on median enrolment date (*n* = 1,002; AUC = 0.73) |
|  |  | “ | Remittance of depressive symptoms at 9-week follow-up, using data from Week 3 (Depression) | Predictive | 1927 (717) | 39.83 | “ | “ | – | AUC = 0.85 | “ | Temporal validation based on median enrolment date (*n* = 1,002; AUC = 0.82) |
| Fusar-Poli et al (2016) [106] | UK | Clinical registry of non-psychotic individuals who underwent CHR assessment (23.7) | 6-year risk of developing psychosis (Psychosis) | Prognostic (onset) | 321 (41) | 1.64 | Theory-driven (previous research); inbuilt variable selection (LASSO) | LASSO cox regression | Cross-validation [N] | *c* = 0.66 | Calibration plot | Geographic validation in patients from independent London health trusts (*n* = 389; *c* = 0.65) |
| Fusar-Poli et al (2017) [107] | UK | Clinical registry of non-psychotic patients (34.4) | 6-year risk of developing psychosis (Psychosis) | Prognostic (onset) | 33,820 (1,001) | 58.88 | Theory-driven (previous research) | Cox proportional hazards regression | – | *c* = 0.80 | Calibration plot | Geographic validation in patients from independent London health trusts (*n* = 54,716; *c* = 0.79; Fusar-Poli, Werbeloff, et al, 2019: *n* = 13,702; *c* = 0.73)  [see also note^5^] |
| Fusar-Poli, Davies, et al (2019) [108] | UK | Clinical registry of non-psychotic patients (34.4) | 6-year risk of developing psychosis (Psychosis) | Prognostic (onset) | 33,820 (1,001) | 52.68 | Theory-driven (update of existing model)[107] | Cox proportional hazards regression | – | *c* = 0.81 | Calibration plot | Geographic validation in patients from independent London health trusts (*n* = 54,716; *c* = 0.81) |
| Galatzer-Levy et al (2014) [109] | Israel | Adults admitted to emergency department following potentially traumatic event (36.3) | Membership of non-remitting PTSD trajectory over 15-month follow-up (PTSD) | Prognostic (course) | 957 (163) | 2.4 | Inbuilt variable selection (Markov Boundary) | S*upport vector machine*; Random Forest; AdaBoost; Ridge regression; Bayesian binary regression | Cross-validation [N] | AUC = 0.77 | – | – |
| Galatzer-Levy et al (2017) [110] | Israel | Adults admitted to emergency department following potentially traumatic event (31.4) | Membership of non-remitting PTSD trajectory over 15-month follow-up (PTSD) | Prognostic (course) | 152 (26) | – | Inbuilt variable selection (recursive feature elimination) | Support vector machine | Cross-validation [N] | AUC = 0.93 | – | – |
| **Author (Year)** | **Country** | **Study setting (mean age or age range, years)** | **Outcome (Psychiatric domain)** | **Model type** | **Sample size**  **(*N* events)** | **EPV^1^** | **Predictor selection** | **Prediction modelling method^2^** | **Internal validation**  **[Valid: Y/N]** | **Discrimination^3^** | **Calibration** | **External validation** |
| Gan et al (2011) [111] | China | Patients initially treated for major depressive episode and later diagnosed with either bipolar disorder or unipolar depression (30.6) | Differentiation of bipolar disorder I or II from unipolar depression at 1-year follow-up (Bipolar) | Prognostic (onset) | 268 (169) | 8.89 | Univariable analyses; forward selection | Logistic regression | Bootstrapping [N] | AUC = 0.82 | – | – |
|  |  | Patients initially treated for major depressive episode and later diagnosed with either bipolar disorder I or unipolar depression (30.2) | Differentiation of bipolar disorder I from unipolar depression at 1-year follow-up (Bipolar) | Prognostic (onset) | 135 (36) | 1.89 | “ | “ | “ | AUC = 0.93 | – | – |
|  |  | Patients initially treated for major depressive episode and later diagnosed with either bipolar disorder II or unipolar depression (30.7) | Differentiation of bipolar disorder II from unipolar depression at 1-year follow-up (Bipolar) | Prognostic (onset) | 232 (133) | 7 | “ | “ | “ | AUC = 0.82 | – | – |
| Ge, Jiang, et al (2020) [112] | China | Patients with major depressive disorder diagnosis within hospital electronic medical record (45.2) | Classified as displaying suicidal ideation (Depression) | Diagnostic | 1,916 (319) | 10.29 | – | Neural network | Random split sample [N] | AUC = 0.74 | – | – |
| Ge, Li, et al (2020) [113] | China | Young people who experienced 2013 Lushan earthquake (12.1) | Probable PTSD at 3-month follow-up (PTSD) | Prognostic (onset) | 2,099 (803) | 33.46 | Iterative testing of specified predictor domains | Extreme gradient boosting | Cross-validation [N] | AUC = 0.80 | – | – |
| Gilman et al (2012) [114] | USA | Adults within longitudinal household survey with lifetime major depressive disorder diagnosis (n.a.) | Transition to bipolar I disorder during 3-year follow-up (Depression) | Prognostic (course) | 6,214 (245) | 14.41 | *A priori* based on available data | Logistic regression | – | AUC = 0.72 | ­– | – |
| Gonzalez-Gutierrez et al (2016) [115] | Spain | Outpatients with chronic obstructive pulmonary disease (66.2) | Presence of anxiety or depression (Mixed Affective) | Diagnostic | 204 (74) | 5.29 | Univariable analyses; forward selection | Logistic regression | – | AUC = 0.77 | – | – |
| Gu et al (2020) [116] | Austria; Australia; France; Germany; Greece; Israel;  Italy; Norway; Spain; UK; USA | Drug-naïve Parkinson’s patients within multi-centre longitudinal study (64.8) | Clinically-significant depressive symptoms at 2-year follow-up (Depression) | Prognostic (onset) | 312 (66) | 2.64 | Theory-driven (previous research); univariable analyses; stepwise selection | *Extreme gradient boosting*; Logistic regression | Random split sample [N] | AUC = 0.94 | Calibration plot; Hosmer-Lemeshow | – |
| Hafeman et al (2017) [117] | USA | Offspring of parents with bipolar disorder I or II who had not yet developed BPSD (11.9) | 5-year risk of developing BPSD (Bipolar) | Prognostic (onset) | 412 (54) | 7.14 | Theory-driven (previous research) | Cox proportional hazards regression | Bootstrapping [Y] | AUC = 0.76 | Calibration plot; Hosmer-Lemeshow | – |
| **Author (Year)** | **Country** | **Study setting (mean age or age range, years)** | **Outcome (Psychiatric domain)** | **Model type** | **Sample size**  **(*N* events)** | **EPV^1^** | **Predictor selection** | **Prediction modelling method^2^** | **Internal validation**  **[Valid: Y/N]** | **Discrimination^3^** | **Calibration** | **External validation** |
| Haidl et al (2018) [118] | Germany, Finland, Netherlands,  Spain, UK | CHR subjects within longitudinal study (23) | Conversion to psychosis during 18-month follow-up (Psychosis) | Prognostic (onset) | 235 (36) | 5.14 | Theory-driven (update of existing model) | Cox proportional hazards regression | Bootstrapping [N] | *c* = 0.77 | – | – |
| Han, Fang, et al (2020) [119] | China | School-aged children diagnosed with ADHD vs non-matched controls (8.3) | ADHD group classification (ADHD) | Diagnostic | 159 (95) | 1.9 | Univariable analyses | Support vector machine | Cross-validation [N] | AUC = 0.82 | – | – |
| Han, Lee, et al (2020) [120] | USA | Adolescents within longitudinal survey  (12-17) | Misuse of opioid pain relievers or heroin in past year (SUDs) | Diagnostic | 41,579 (1,051) | 26.95 | Theory-driven (previous research); inbuilt variable selection | Artificial neural networks; random forest; gradient boosting; *penalised logistic regression* | Random split sample [N] | AUC = 0.82 | – | – |
| Han, Tomasik, et al (2020) [121] | UK | Adults with current MDD diagnosis vs randomly selected low mood controls (25.9) | Presence of current MDD diagnosis (Depression) | Diagnostic | 140 (53) | 0.18 | Inbuilt variable selection (LASSO) | LASSO logistic regression | Random split sample [N] | AUC = 0.94 | – | – |
| Hariman et al (2020) [122] | Hong Kong | Patients with psychotic spectrum disorders discharged from psychiatric units (41.1) | Unplanned readmission within 28 days of discharge (Psychosis) | Prognostic (course) | 30,707 (2,178) | 50.65 | Theory-driven (previous research); backward selection | Logistic regression | Random split sample [N] | *c* = 0.68 | Hosmer-Lemeshow | – |
| Harrington et al (2019) [123] | USA | US veterans within electronic medical record (64) | Lifetime ‘likely PTSD’ (PTSD) | Diagnostic | 485 (198) | 7.92 | Inbuilt feature selection (LASSO) | LASSO logistic regression | Cross-validation [N] | AUC = 0.95 | – | – |
| Hatton et al (2019) [124] | UK | Older primary care patients within control arm of previous RCT (77.7) | Significant depressive symptoms at 12-month follow-up (Depression) | Prognostic (onset) | 284 (n.a.) | – | Backward selection | Extreme gradient boosting | Random split sample, repeated [N] | AUC = 0.72 | – | – |
| Haynos et al (2020) [125] | USA | Females with heterogenous eating disorder diagnoses within longitudinal study (32.4) | Continued eating disorder diagnosis at 1-year follow-up (Eating Disorders) | Prognostic (course) | 320 (299) | 5.64 | Theory-driven (previous research); inbuilt variable selection (elastic net) | *Elastic net logistic regression*; Logistic regression; Random forest | Cross-validation [N] | AUC = 0.62 | – | – |
|  |  | “ | Continued eating disorder diagnosis at 2-year follow-up (Eating Disorders) | Prognostic (course) | 277 (118) | 2.23 | “ | “ | “ | AUC = 0.61 | – | – |
| Hengartner et al (2017) [126] | Switzerland | CHR subjects referred to early recognition centre (20.5) | 3-year risk of conversion to psychosis (Psychosis) | Prognostic (onset) | 188 (24) | 1.85 | Univariable analyses; stepwise selection | Cox proportional hazards regression | – | AUC = 0.85 | Calibration statistics (slope); Hosmer-Lemeshow | – |
| **Author (Year)** | **Country** | **Study setting (mean age or age range, years)** | **Outcome (Psychiatric domain)** | **Model type** | **Sample size**  **(*N* events)** | **EPV^1^** | **Predictor selection** | **Prediction modelling method^2^** | **Internal validation**  **[Valid: Y/N]** | **Discrimination^3^** | **Calibration** | **External validation** |
| Hettige et al (2017) [127] | Canada | Patients diagnosed with a schizophrenia spectrum disorder (41.7) | Retrospectively-reported lifetime suicide attempt (Psychosis) | Diagnostic | 345 (131) | 4.85 | Theory-driven (previous research); inbuilt variable selection | *LASSO regression*; Elastic net regression; Random forest; Support vector machine | Cross-validation [N] | AUC = 0.71 | – | – |
| Higginson and Priest (1996) [128] | UK | Carers or significant others of patients receiving palliative care for cancer (n.a.) | Severe vs mild/moderate anxiety during month before bereavement (Anxiety) | Prognostic (onset) | 287 (84) | 16.8 | Available data | Discriminant function analysis | – | Sen. = 0.75  Spe. = 0.51 | – | – |
| Hilbert et al (2020) [129] | Germany | Outpatients undergoing cognitive-behaviour therapy (39.3) | Post-treatment remission (Depression) | Prognostic (course) | 1,851 (1,079) | 3.18 | Inbuilt variable selection (recursive feature elimination) | *Random forest*; Support vector machine; Neural network; Ensemble strategies | Random split sample [N] | BAC = 0.59 | – | – |
| Hirschfeld et al (1998) [130] | USA | Adults with chronic major depression treated with sertraline vs imipramine during double-blind RCT (41.1) | Treatment response following 12-week trial (Depression) | Predictive | 623 (324) | 11.57 | Univariable analyses | Logistic regression | – | Acc. = 0.67 | – | – |
| Hoogendoorn et al (2017) [131] | Netherlands | Patients with social anxiety disorder within RCT for internet-based guided self-help intervention (n.a.) | Reliable symptom improvement following 12-week trial (Anxiety) | Prognostic (course) | 69 (48) | – | Ranking-based method | L*ogistic regression*; decision trees; Random forest | Cross-validation [N] | AUC = 0.83 | – | – |
| Huang et al (2010) [132] | China | Survey of individuals who experienced 1998 floods in Hunan province (n.a.) | Probable PTSD diagnosis (PTSD) | Diagnostic | 25,478 (2,236) | 122.95 | Forward selection | Logistic regression | Random split sample [N] | AUC = 0.85 | – | – |
| Huang et al (2014) [133] | USA | Matched sample of depressed vs non-depressed patients within clinical registry (n.a.) | Subsequent depression diagnosis  (Depression) | Prognostic (onset) | 35,000 (5,000) | – | Inbuilt variable selection (LASSO) | LASSO logistic regression | Random split sample [N] | AUC = 0.80 | – | – |
|  |  | Adult patients treated for depression using medication or psychotherapy (n.a.) | Symptom improvement after medication treatment (Depression) | Predictive | 5,651 (1,576) | – | “ | “ | “ | AUC = 0.66 | – | – |
|  |  | Adult patients treated for depression using medication or psychotherapy (n.a.) | Symptom improvement after psychotherapy treatment (Depression) | Predictive | 5,651 (886) | – | “ | “ | “ | AUC = 0.75 | – | – |
|  |  | Adult patients treated for depression using medication or psychotherapy (n.a.) | Severe vs minimal/mild depression (Depression) | Diagnostic | 5,651 (1,301) | – | “ | “ | “ | AUC = 0.72 | – | – |
| **Author (Year)** | **Country** | **Study setting (mean age or age range, years)** | **Outcome (Psychiatric domain)** | **Model type** | **Sample size**  **(*N* events)** | **EPV^1^** | **Predictor selection** | **Prediction modelling method^2^** | **Internal validation**  **[Valid: Y/N]** | **Discrimination^3^** | **Calibration** | **External validation** |
| Hughes et al (2020) [134] | USA | Adults with major depressive disorder who received psychiatric care, drawn from coded electronic health records (48.5) | Stable treatment regimen, defined by use of same antidepressant prescription for at least 90 days (Depression) | Prognostic (course) | 53,643 (25,141) | 2.72 | *A priori* based on available data; probabilistic topic modelling | *Ridge logistic regression*; Extremely randomised trees | Random split sample [N] | AUC = 0.63 | – | Geographic validation using patients from independent site (*n* = 26,176; AUC = 0.62) |
| Iniesta et al (2016) [135] | Belgium, Croatia, Denmark, Germany, Italy, Poland, Slovenia, UK | Patients with major depressive disorder within 12-week comparative study of escitalopram vs nortriptyline (42.7) | Remission of depressive symptoms  (Depression) | Predictive | 793 (326) | 2.34 | Inbuilt variable selection (elastic net) | Elastic net logistic regression | Cross-validation [N] | AUC = 0.72 | – | – |
|  |  | “ | Adequate completion of treatment trial (Depression) | Predictive | 793 (710) | 5.11 | “ | “ | “ | AUC = 0.63 | – | – |
|  |  | “ | Treatment resistance (lack of response following two trials) (Depression) | Predictive | 793 (105) | 0.76 | “ | “ | “ | AUC = 0.67 | – | – |
| Ising et al (2016) [136] | Netherlands | Help-seeking CHR patients drawn from RCT (22.8) | Conversion to psychosis during 18-month follow-up (Psychosis) | Prognostic (onset) | 167 (32) | – | Univariable analyses; backward selection | Cox proportional hazards regression | Bootstrapping [N] | AUC = 0.81 | – | – |
| Jimenez-Serrano et al (2015) [137] | Spain | Postpartum women within prospective hospital study (32) | Major depressive episode during 8-month follow-up (Depression) | Prognostic (onset) | 1,397 (160) | 10 | Theory-driven (practical utility or relevance); inbuilt variable selection | *Naïve Bayes*; Logistic regression; Support vector machine; Artificial neural network | Random split sample [N] | AUC = 0.75 | – | – |
| Jin, Wu, and Di Capua (2015) [138] | USA | Diabetic patients within two clinical trials (53.4) | Comorbid major depression (Depression) | Prognostic (onset) | 1,793 (786) | 39.25 | Theory-driven (previous research and clinical utility); Correlation-based subset evaluation | *Ridge logistic regression*; Mulitlayer perceptron; Support vector machine; Random forest | Cross-validation [N] | AUC = 0.81 | – | – |
| Jin, Wu, Vidyanti, et al (2015) [139] | USA | Diabetic patients within clinical trial (53.3) | Major depression at 6-month follow-up (Depression) | Prognostic (onset) | 853 (n.a.) | – | Univariable analyses; backward selection | Poisson regression | Random split sample [N] | AUC = 0.88 | – | – |
|  |  | “ | Major depression at 12-month follow-up (Depression) | Prognostic (onset) | “ | – | “ | “ | “ | AUC = 0.91 | – | – |
|  |  | “ | Major depression at 18-month follow-up (Depression) | Prognostic (onset) | “ | – | “ | “ | “ | AUC = 0.89 | – | – |
| **Author (Year)** | **Country** | **Study setting (mean age or age range, years)** | **Outcome (Psychiatric domain)** | **Model type** | **Sample size**  **(*N* events)** | **EPV^1^** | **Predictor selection** | **Prediction modelling method^2^** | **Internal validation**  **[Valid: Y/N]** | **Discrimination^3^** | **Calibration** | **External validation** |
| Jing et al (2020) [140] | USA | Children of men with lifetime substance use disorder diagnosis or non-SUD psychiatric disorder within longitudinal study (30) | Substance use disorder diagnosis at age 30 (SUDs) | Prognostic (onset) | 694 (n.a.) | – | Inbuilt variable selection (variable importance) | Logistic regression; *Random forest*; Adaptive boosting; Naïve Bayes; Support vector machine; K-nearest neighbour; Deep neural network | Cross-validation [N] | AUC = 0.86 | – | ­– |
| Kajiwara et al (2016) [141] | Japan | Hospital outpatients (53.3) | Current presence of anxiety and/or depressive symptoms (Mixed Affective) | Diagnostic | 358 (169) | 33.8 | Theory-driven (previous research); forward selection | Logistic regression | Bootstrapping [N] | *c* = 0.63 | Hosmer-Lemeshow | – |
| Karstoft, Galatzer-Levy et al (2015) [142] | Israel | Adults admitted to emergency department following potentially traumatic event (18-70) | Membership of non-remitting PTSD trajectory over 5-month follow-up (PTSD) | Prognostic (course) | 957 (163) | – | Inbuilt variable selection (Markov Boundary) | Support vector machine | Cross-validation [N] | AUC = 0.75 | – | – |
| Karstoft, Statnikov et al (2015) [143] | Denmark | Longitudinal study of soldiers deployed to Afghanistan (26.2) | Membership of symptomatic vs resilient trajectory group 2.5 years post-deployment, based on pre-deployment factors only (PTSD) | Prognostic (onset) | 561 (n.a.) | – | Inbuilt variable selection (Markov Boundary); univariable analyses | Support vector machine | Cross-validation [N] | AUC = 0.84 | – | – |
|  |  | “ | Membership of symptomatic vs resilient group 2.5 years post-deployment, based on pre- and post-deployment factors (PTSD) | Prognostic (onset) | “ | – | “ | “ | “ | AUC = 0.88 | ­– | – |
| Kautzky et al (2017) [144] | Austria, Belgium, France, Greece, Israel, Italy | Adult patients diagnosed with major depressive disorder (49.4) | Treatment resistance following at least two adequate antidepressant trials (Depression) | Predictive | 480 (183) | 3.81 | Inbuilt variable selection (variable importance) | Random forest | Random split sample [N] | Acc. = 0.74  Sen. = 0.63  Spe. = 0.80  PPV = 0.66  NPV = 0.78 | – | – |
|  |  | “ | Symptom remission (Depression) | Predictive | 480 (140) | 2.92 | “ | “ | “ | Acc. = 0.85  Sen. = 0.64  Spe. = 0.93  PPV = 0.78  NPV = 0.87 | – | – |
| **Author (Year)** | **Country** | **Study setting (mean age or age range, years)** | **Outcome (Psychiatric domain)** | **Model type** | **Sample size**  **(*N* events)** | **EPV^1^** | **Predictor selection** | **Prediction modelling method^2^** | **Internal validation**  **[Valid: Y/N]** | **Discrimination^3^** | **Calibration** | **External validation** |
| Kautzky et al (2018) [145] | Austria, Belgium, France, Germany, Greece Israel, Italy, Switzerland | Adult patients diagnosed with major depressive disorder (51.7) | Treatment resistance following at least two adequate antidepressant trials (Depression) | Predictive | 552 (362) | 7.7 | Inbuilt variable selection (variable importance) | Random forest | Cross-validation [N] | Acc. = 0.75  Sen. = 0.82  Spe. = 0.63  PPV = 0.80  NPV = 0.68 | – | – |
| Kautzky et al (2019) [146] | Austria; Belgium;  France; Germany; Greece; Israel; Italy;  Switzerland | Adult patients with major depressive disorder (52.6) | Treatment resistance following at least two adequate antidepressant trials (Depression) | Predictive | 602 (309) | 17.17 | Theory-driven (previous research); inbuilt variable selection | Elastic net logistic regression | Cross-validation [N] | Acc. = 0.87  Sen. = 0.95  Spe. = 0.78 | – | Geographic validation in independent patient sample (*n* = 314; Acc. = 0.87; Sen. = 0.86; Spe. = 0.88) |
| Kendler et al (2019) [147] | Sweden | Patients with substance-induced psychotic disorder (32.1) | Conversion to schizophrenia (Psychosis) | Prognostic (course) | 7,606 (445) | 44.5 | Available data | Cox proportional hazards regression | Random split sample [N] | AUC = 0.74 | – | – |
| Kessler et al (2014) [148] | Australia, Belgium, Brazil, Bulgaria, China, Colombia, France, Germany, Israel, Italy, Japan, Lebanon, Mexico, Netherlands, New Zealand, Nigeria, Peru, Portugal, Romania, South Africa, Spain, Ukraine,  UK, USA | Individuals within community survey retrospectively reporting lifetime trauma exposure (n.a.) | Current PTSD diagnosis (PTSD) | Diagnostic | 47,466 (1,899) | – | Inbuilt variable selection | Super Learner ensemble | Cross-validation [N] | AUC = 0.98 | – | – |
| Kessler et al (2015) [149] | USA | Active duty soldiers hospitalised with a psychiatric disorder (n.a.) | Suicide in the 12 months following hospital discharge (Transdiagnostic) | Prognostic (course) | 40,820 (68) | 0.16 | Theory-driven (previous research); univariable analyses; regression trees; penalised regression | Elastic net logistic regression | Cross-validation [N] | AUC = 0.85 | – | – |
| **Author (Year)** | **Country** | **Study setting (mean age or age range, years)** | **Outcome (Psychiatric domain)** | **Model type** | **Sample size**  **(*N* events)** | **EPV^1^** | **Predictor selection** | **Prediction modelling method^2^** | **Internal validation**  **[Valid: Y/N]** | **Discrimination^3^** | **Calibration** | **External validation** |
| Kessler et al (2016) [150] | USA | Adults with major depressive disorder within longitudinal household survey (15-54) | High persistence, or depressive episode lasting 2+ weeks, at 10-12-year follow-up (Depression) | Prognostic (course) | 1,056 (102) | – | Theory-driven (previous model); inbuilt variable selection (LASSO) | Ensemble (regression trees; LASSO regression) | Cross-validation [N] | AUC = 0.71 | – | – |
|  |  | “ | High chronicity, or depressive episode lasting most days throughout previous year (Depression) | Prognostic (course) | 1,056 (89) | – | “ | “ | “ | AUC = 0.63 | – | – |
|  |  | “ | Hospitalisation since baseline (Depression) | Prognostic (course) | 1,056 (61) | – | “ | “ | “ | AUC = 0.73 | – | – |
|  |  | “ | Disability, or ≥50% limitation in ability to perform paid work (Depression) | Prognostic (course) | 1,056 (48) | – | “ | “ | “ | AUC = 0.74 | – | – |
|  |  | “ | Attempted suicide since baseline (Depression) | Prognostic (course) | 1,056 (34) | – | “ | “ | “ | AUC = 0.76 | – | – |
| Kim et al (2015) [151] | South Korea | ADHD youth within 8-week open-label methylphenidate trial (9.6) | Good vs poor response to methylphenidate treatment (ADHD) | Prognostic (course) | 78 (48) | 1.41 | Wrapper subset evaluation | *Support vector machine*; Decision trees; Random forest; Ridge regression | Cross-validation [N] | AUC = 0.84 | – | – |
| Kim et al (2019) [152] | South Korea | Community sample of older adults within ecological momentary assessment trial (78) | Probable vs no depression at 2-week follow-up (Depression) | Prognostic (onset) | 47 (18) | – | Stepwise selection | Logistic regression | Random split sample, repeated [N] | AUC = 0.96 | – | – |
| King et al (2008) [153] | Chile, Estonia, Portugal, Netherlands, Slovenia, Spain, UK | Non-depressed adult primary care patients within prospective study (48.9) | Onset of major depression during 12-month follow-up (Depression) | Prognostic (onset) | 5,216 (402) | 10.31 | Stepwise selection | Logistic regression with shrinkage factor | – | *c* = 0.79 | – | Geographic validation in (i) Chilean sample (*n* = 1,732; *c* = 0.71), (ii) using 12-24-month follow-up data (King et al, 2013: *n* = 2,440; *c* = 0.73), and (iii) US general population (Nigatu et al, 2016: *n* = 24,311; *c* = 0.71) |
| **Author (Year)** | **Country** | **Study setting (mean age or age range, years)** | **Outcome (Psychiatric domain)** | **Model type** | **Sample size**  **(*N* events)** | **EPV^1^** | **Predictor selection** | **Prediction modelling method^2^** | **Internal validation**  **[Valid: Y/N]** | **Discrimination^3^** | **Calibration** | **External validation** |
| King, Bottomley, et al (2011) [154] | Chile, Estonia, Portugal, Netherlands, Slovenia, Spain, UK | Prospective study of adult primary care patients without generalized anxiety disorder at baseline (18-75) | Onset of generalized anxiety or panic syndrome at 6-month follow-up (Anxiety) | Prognostic (onset) | 4,905 (270) | 7.1 | Backward selection | Logistic regression with shrinkage factor | – | *c* = 0.78 | – | Geographic validation (at 6-month follow-up) in (i) external samples from Chile (*n* = 2,825; *c* = 0.71), Estonia (*n* = 1,094; *c* = 0.73), and The Netherlands (*n* = 1,221; *c* = 0.81), and (ii) the US general population (Nigatu and Wang, 2019: *n* = 24,626; *c* = 0.62) |
|  |  | “ | Onset of generalized anxiety or panic syndrome at 24-month follow-up (Anxiety) | Prognostic (onset) | 4,905 (n.a.) | – | “ | “ | – | *c* = 0.73 | – | – |
|  |  | “ | Onset of generalized anxiety or panic syndrome at 6- and/or 24-month follow-up (Anxiety) | Prognostic (onset) | 4,905 (525) | 13.82 | “ | “ | – | *c* = 0.75 | – | – |
| King, Marston, et al (2011) [155] | Estonia; Netherlands; Portugal; Slovenia; Spain; UK | Adult primary care patients within prospective study (49) | Hazardous drinking at 6-month follow-up (SUDs) | Prognostic (onset) | 6,193 (175) | 4.61 | Univariable analyses; stepwise selection | Logistic regression with shrinkage factor | – | *c* = 0.84 | – | Geographic validation in Chilean sample (*n* = 2,462; *c* = 0.78) |
| Kirchebner et al (2020) [156] | Switzerland | Offenders in forensic psychiatric hospital with schizophrenia spectrum disorder (34.7) | Prolonged length of stay; >220 weeks (Psychosis) | Prognostic (course) | 143 (71) | 0.79 | Theory-driven (previous research); forward selection | *Boosted trees*; K-nearest neighbour; support vector machine; logistic regression | Cross-validation [N] | AUC = 0.67 | – | – |
|  |  | “ | Prolonged length of stay; >278 weeks (Psychosis) | Prognostic (course) | 143 (n.a.) | – | “ | Boosted trees; K-nearest neighbour; *Support vector machine*; Logistic regression | “ | AUC = 0.71 | – | – |
| Klein et al (2018) [157] | Netherlands | Adult patients within randomised control trial for cognitive therapy (46.8) | Recurrence during 2-year follow-up (Depression) | Predictive | 235 (104) | 13 | Theory-driven (previous research; EPV); backward selection | Cox proportional hazards regression | ­Bootstrapping [N] | *c* = 0.56 | Calibration plot; calibration slope | Geographic validation using independent RCT  (*n* = 205; *c* = 0.59) |
| Kotlicka-Antczak et al (2019) [158] | Poland | Prospective study of CHR individuals referred to secondary care programme (18.8) | Conversion to psychosis during follow-up period (Psychosis) | Prognostic (onset) | 105 (n.a.) | – | Theory-driven (previous research) | Cox proportional hazards regression | Bootstrapping [Y] | *c* = 0.78 | Calibration plot | – |
| **Author (Year)** | **Country** | **Study setting (mean age or age range, years)** | **Outcome (Psychiatric domain)** | **Model type** | **Sample size**  **(*N* events)** | **EPV^1^** | **Predictor selection** | **Prediction modelling method^2^** | **Internal validation**  **[Valid: Y/N]** | **Discrimination^3^** | **Calibration** | **External validation** |
| Koutsouleris et al (2012) [159] | Germany | Individuals at risk for psychosis within matched prospective study (24.7) | Conversion to psychosis during 4-year follow-up (Psychosis) | Prognostic (onset) | 35 (15) | – | Inbuilt variable selection (recursive feature elimination) | Support vector machine | Cross-validation [Y] | BAC = 0.87  Sen. = 0.80  Spe. = 0.93  PPV = 0.88  NPV = 0.89 | – | – |
| Koutsouleris et al (2016) [160] | Austria, Belgium, Bulgaria, Czech Republic, France, Germany, Israel, Italy, Netherlands, Poland, Romania, Spain, Sweden, Switzerland | Adult patients with schizophrenic, schizophreniform, or schizoaffective disorder within clinical trial with five treatment groups (26.1) | Poor vs good global functioning at 4-week follow-up (Psychosis) | Predictive | 334 (224) | 1.19 | Inbuilt variable selection (forward selection) | Support vector machine | Cross-validation [Y] | BAC = 0.75  Sen. = 0.74  Spe. = 0.76  PPV = 0.86  NPV = 0.59 | – | Validated in independent test set of patients with 4-week follow-up data (*n* = 108; BAC = 0.72) |
|  |  | “ | Poor vs good global functioning at 52-week follow-up (Psychosis) | Predictive | 334 (78) | 0.41 | “ | “ | “ | BAC = 0.74  Sen. = 0.67  Spe. = 0.81  PPV = 0.52  NPV = 0.89 | – | – |
| Koutsouleris et al (2018) [161] | Finland, Germany, Italy, Switzerland, UK | CHR individuals within prospective study  (24.1) | Impaired social functioning during 1-year follow-up (Psychosis) | Prognostic (course) | 116 (66) | – | Inbuilt variable selection (backward elimination) | NeuroMiner | Cross-validation [Y] | AUC = 0.86 | – | – |
|  |  | CHR individuals within prospective study  (24.1) | Impaired role functioning during 1-year follow-up (Psychosis) | Prognostic (course) | 116 (69) | – | “ | “ | “ | AUC = 0.73 | – | – |
|  |  | Individuals with recent-onset depression within prospective study (26.3) | Impaired social functioning during 1-year follow-up (Depression) | Prognostic (course) | 120 (65) | – | “ | “ | “ | AUC = 0.77 | – | – |
|  |  | Individuals with recent-onset depression within prospective study (26.3) | Impaired role functioning during 1-year follow-up (Depression) | Prognostic (course) | 120 (64) | – | “ | “ | “ | AUC = 0.67 | – | – |
| **Author (Year)** | **Country** | **Study setting (mean age or age range, years)** | **Outcome (Psychiatric domain)** | **Model type** | **Sample size**  **(*N* events)** | **EPV^1^** | **Predictor selection** | **Prediction modelling method^2^** | **Internal validation**  **[Valid: Y/N]** | **Discrimination^3^** | **Calibration** | **External validation** |
| Kwakernaak et al (2020) [162] | Netherlands | Patients diagnosed with psychotic spectrum disorder within longitudinal study (32) | Intensive mental healthcare consumption (sheltered housing, inpatient or legal mandatory care) within same data wave (Psychosis) | Diagnostic | 1067 (488) | – | Inbuilt variable selection (information gain) | Logistic regression; Classification tree; *Random forest* | Random split sample [N] | AUC = 0.74 | – | – |
|  |  | “ | Intensive mental healthcare consumption (sheltered housing, inpatient or legal mandatory care) at 3-year follow-up (Psychosis) | Prognostic (course) | 350 (n.a.) | – | “ | “ | “ | AUC = 0.71 | – | – |
| Leightley et al (2019) [163] | UK | Longitudinal cohort of armed forces personnel (n.a.) | Probable PTSD diagnosis (PTSD) | Diagnostic | 13,690 (541) | 13.87 | Inbuilt variable selection (various) | Support vector machine; Random forest; Artificial neural networks; Bagging | Random split sample, repeated [N] | Acc. = 0.89-0.91  Sen. = 0.60-0.70  Spe. = 0.92-0.98 | – | – |
| Leighton, Krishnadas, et al (2019) [164] | UK | Patients with first episode psychosis in secondary care (25.2) | Employment, Education or Training status at 1-year follow-up (Psychosis) | Prognostic (course) | 75 (32) | 1 | Inbuilt variable selection (elastic net) | Elastic net logistic regression | Cross-validation [N] | – | – | Validated in independent cohort of first-episode psychosis patients recruited to earlier study (*n* = 79; AUC = 0.88) |
|  |  | “ | Point remission at 1-year follow-up (Psychosis) | Prognostic (course) | 71 (40) | 1.25 | “ | “ | “ | – | – | Validated in independent cohort of first-episode psychosis patients recruited to earlier study (*n* = 79; AUC = 0.65) |
|  |  | “ | Period remission at 1-year follow-up (Psychosis) | Prognostic (course) | 67 (33) | 1.03 | “ | “ | “ | – | – | Validated in independent cohort of first-episode psychosis patients recruited to earlier study (*n* = 79; AUC = 0.63) |
| **Author (Year)** | **Country** | **Study setting (mean age or age range, years)** | **Outcome (Psychiatric domain)** | **Model type** | **Sample size**  **(*N* events)** | **EPV^1^** | **Predictor selection** | **Prediction modelling method^2^** | **Internal validation**  **[Valid: Y/N]** | **Discrimination^3^** | **Calibration** | **External validation** |
| Leighton, Upthegrove, et al (2019) [165] | UK | Patients with first-episode psychosis in early intervention services (21.3) | Symptom remission at 1-year follow-up  (Psychosis) | Prognostic (course) | 673 (320) | 1.96 | Inbuilt variable selection (elastic net) | Elastic net logistic regression | Cross-validation [Y] | AUC = 0.70 | – | Geographic validation in combined data from two Scottish longitudinal studies (*n* = 131; AUC = 0.68) and RCT trial data (*n* = 338; AUC = 0.62) |
|  |  | “ | Social recovery (based on global functioning) at 1-year follow-up (Psychosis) | Prognostic (course) | 829 (388) | 2.38 | “ | “ | “ | AUC = 0.73 | – | Validated using RCT trial data (*n* = 518; AUC = 0.57) |
|  |  | “ | Vocational recovery (in employment, education, or training) at 1-year follow-up (Psychosis) | Prognostic (course) | 807 (436) | 2.68 | “ | “ | “ | AUC = 0.74 | – | Geographic validation in combined data from two Scottish longitudinal studies (*n* = 142; AUC = 0.87) and RCT trial data (*n* = 553; AUC = 0.66) |
|  |  | “ | Quality of life at 1-year follow-up (Psychosis) | Prognostic (course) | 729 (328) | 2.01 | “ | “ | “ | AUC = 0.70 | – | Geographic validation in data from two Scottish longitudinal studies (*n* = 47; AUC = 0.68) and RCT trial (*n* = 226; AUC = 0.56) |
| Lencz et al (2006) [166] | USA | CHR patients within early recognition programme (16.5) | Conversion to psychosis during follow-up (Psychosis) | Diagnostic | 33 (12) | – | Univariable analyses; stepwise selection | Cox proportional hazards regression | ­– | Acc. = 0.80  Sen. = 0.82  Spe. = 0.79  PPV = 0.69  NPV = 0.88 | – | – |
| Lenhard et al (2018) [167] | Sweden | Adolescents receiving internet-delivered cognitive behaviour therapy within randomised control trial (14.4) | Treatment response (>35% symptom reduction) at 3-month follow-up (OCD) | Prognostic (course) | 61 (25) | 0.54 | Univariable analyses | *Linear model with best subset predictor selection*; Elastic net logistic regression; Random forest; Support vector machine | Random split sample [N] | Acc. = 0.83 | – | – |
| Lerthattasilp et al (2020) [168] | Thailand | Child outpatients suspected of having autism spectrum disorder (3.7) | ASD diagnosis (ASD) | Diagnostic | 139 (104) | 1.22 | Theory-driven (previous research); univariable analyses; backward selection | Logistic regression | Bootstrapping [N] | AUC = 0.83 | Hosmer-Lemeshow | – |
| Levin et al (2005) [169] | USA | Patients with mild traumatic brain injury (31.5) | Onset of major depression 3 months post-injury (Depression) | Prognostic (onset) | 129 (15) | 1.67 | Backward selection | Logistic regression | Bootstrapping [N] | AUC = 0.86 | – | – |
| **Author (Year)** | **Country** | **Study setting (mean age or age range, years)** | **Outcome (Psychiatric domain)** | **Model type** | **Sample size**  **(*N* events)** | **EPV^1^** | **Predictor selection** | **Prediction modelling method^2^** | **Internal validation**  **[Valid: Y/N]** | **Discrimination^3^** | **Calibration** | **External validation** |
| Lewis et al (2019) [170] | UK | Trauma-exposed young people within longitudinal study (18) | Lifetime PTSD diagnosis at age 18 years (PTSD) | Prognostic (onset) | 605 (149) | 12.42 | Theory-driven (previous research) | Logistic regression | Bootstrapping [Y] | AUC = 0.74 | Calibration plot; calibration statistics (intercept, slope) | – |
| Librenza-Garcia et al (2020) [171] | Brazil | Adults within longitudinal study of Brazilian civil servants (51.8) | Presence of depression at either baseline or follow-up (Depression) | Diagnostic | 13,922 (1,085) | 68.82 | Theory-driven (practical utility or relevance); inbuilt variable selection (elastic net) | Elastic net logistic regression | Random split sample [N] | AUC = 0.79 | – | – |
|  |  | “ | New depressive episode at follow-up study wave (Depression) | Prognostic (onset) | 13,922 (499) | 29.35 | “ | “ | “ | AUC = 0.71 | – | – |
|  |  | “ | Persistent depression at both study waves (Depression) | Prognostic (course) | 13,922 (160) | 9.41 | “ | “ | “ | AUC = 0.90 | – | – |
| Lin et al (2007) [172] | Taiwan | Hospitalised patients with schizophrenia receiving zotepine treatment (38) | Clinical response at 4-week follow-up (Psychosis) | Prognostic (course) | 100 (78) | 6.5 | Univariable analyses; forward selection | Logistic regression | – | AUC = 0.90 | – | – |
| Lin et al (2011) [173] | Taiwan | Hospitalised patients with major depressive disorder receiving fluoxetine treatment (45.6) | Stable clinical response (>50% symptom reduction) at 6-week follow-up (Depression) | Prognostic (course) | 112 (58) | 14.5 | Univariable analyses | Chi-square and *t*-tests | – | AUC = 0.93 | – | – |
| Lin et al (2012) [174] | Taiwan | Hospitalised patients with schizophrenia receiving zotepine treatment (36.9) | Symptom remission at 4-week follow-up (Psychosis) | Prognostic (course) | 100 (21) | 1.9 | Univariable analyses; backward selection | Logistic regression | – | AUC = 0.82 | – | – |
| Lin et al (2018) [175] | Taiwan | Patients with major depressive disorder treated with antidepressants (43.7) | Treatment response at 8-week follow-up (Depression) | Prognostic (course) | 421 (257) | – | Univariable analyses | Multilayer feedforward neural network | Cross-validation [N] | AUC = 0.82 | – | – |
|  |  | “ | Symptom remission at 8-week follow-up (Depression) | Prognostic (course) | 421 (139) | – | “ | “ | “ | AUC = 0.81 | – | – |
| Liu et al (2015) [176] | USA | Adults reporting lifetime panic disorder within longitudinal survey (45.6) | Recurrence of panic disorder after 2+ months without an attack (Anxiety) | Prognostic (course) | 949 (171) | – | Forward and backward selection | Logistic regression with shrinkage factor | – | *c* = 0.79 | Hosmer-Lemeshow | Geographic validation in two independent US census regions (*n* = 732; *c* = 0.73) |
| Liu et al (2017) [177] | China | Stroke patients (64.3) | Presence of depression one month after stroke (Depression) | Prognostic (onset) | 562 (226) | 8.07 | Backward selection | Logistic regression; *Decision trees* | Random split sample [N] | AUC = 0.85 | – | – |
| **Author (Year)** | **Country** | **Study setting (mean age or age range, years)** | **Outcome (Psychiatric domain)** | **Model type** | **Sample size**  **(*N* events)** | **EPV^1^** | **Predictor selection** | **Prediction modelling method^2^** | **Internal validation**  **[Valid: Y/N]** | **Discrimination^3^** | **Calibration** | **External validation** |
| Lorenzo-Luaces et al (2017) [178] | Netherlands | Patients with MDD within RCT comprising brief therapy, CBT or TAU conditions (36.4) | Absence of MDD at 18-24 follow-up  (Depression) | Predictive | 622 (450) | 34.6 | Theory-driven (previous research); inbuilt variable selection (LASSO) | LASSO logistic regression | Bootstrapping [Y] | *c* = 0.73 | – | – |
| Lorimer et al (2021) [179] | UK | Patients who completed low-intensity cognitive behavioural therapy for depression or anxiety (43) | Relapse during 12-month follow-up (Mixed Affective) | Prognostic (course) | 317 (223) | 1.58 | Leave-one-variable-out loops | Extreme gradient boosting | Cross-validation [N] | AUC = 0.84 | – | – |
| Maarsingh et al (2011) [180] | Netherlands | Older patients consulting primary care physician for persistent dizziness (78.5) | Current presence of anxiety and/or depressive disorder (Mixed Affective) | Diagnostic | 415 (90) | 3.46 | Univariable analyses; backward selection | Logistic regression | Bootstrapping [N] | AUC = 0.82 | Calibration plot; Hosmer-Lemeshow | – |
| Maarsingh et al (2018) [181] | Netherlands | Older adults with current or lifetime depressive disorder (70.4) | Presence of major depressive disorder at 2-year follow-up (Depression) | Prognostic (course) | 270 (111) | 4.63 | Theory-driven (previous research; EPV); univariable analyses | Logistic regression | Bootstrapping [N] | AUC = 0.75 | Calibration plot; Hosmer-Lemeshow | Temporal validation using data from earlier data collection period (*n* = 197; AUC = 0.70) |
| Mak et al (2011) [182] | Singapore | Patients with systemic lupus erythematosus (40.5) | Clinically-significant anxiety symptoms (Anxiety) | Diagnostic | 60 (23) | 1.21 | Univariable analyses | Logistic regression | – | AUC = 0.90 | – | – |
| Mason et al (2004) [183] | Australia | Help-seeking youth at risk for psychosis (17.3) | Conversion to psychosis during follow-up (Psychosis) | Prognostic (onset) | 74 (37) | – | Theory-driven (previous research); forward selection | Logistic regression | – | Sen. = 0.84  Spe. = 0.86 | – | – |
| Mechelli et al (2017) [184] | Australia | Matched sample of CHR individuals in specialist clinic who did and did not make transition to psychosis (19.5) | Conversion to psychosis during follow-up (Psychosis) | Prognostic (onset) | 198 (99) | 6.19 | Inbuilt variable selection | Support vector machine | Cross-validation [Y] | Acc. = 0.65  Sen. = 0.69  Spe. = 0.61 | – | – |
|  |  | Matched sample of CHR individuals in specialist clinic with poor vs good functioning (19.7) | Poor social and occupational functioning at last follow-up (Psychosis) | Prognostic (course) | 96 (48) | 3 | “ | “ | “ | Acc. = 0.63  Sen. = 0.63  Spe. = 0.63 | – | – |
| Meehan et al (2020) [185] | UK | Youth within longitudinal birth cohort exposed to severe childhood victimisation (18) | Psychiatric disorder diagnosis at age 18 (Transdiagnostic) | Prognostic (onset) | 505 (308) | 11.4 | Theory-driven (previous research); inbuilt variable selection (LASSO) | LASSO logistic regression | Cross-validation [Y] | AUC = 0.69 | Calibration plot | – |
|  |  | “ | Internalizing disorder diagnosis at age 18 (Transdiagnostic) | Prognostic (onset) | 504 (194) | 7.19 | “ | “ | “ | AUC = 0.66 | “ | – |
|  |  | “ | Externalizing disorder diagnosis at age 18 (Transdiagnostic) | Prognostic (onset) | 505 (217) | 8.1 | “ | “ | “ | AUC = 0.73 | “ | – |
| **Author (Year)** | **Country** | **Study setting (mean age or age range, years)** | **Outcome (Psychiatric domain)** | **Model type** | **Sample size**  **(*N* events)** | **EPV^1^** | **Predictor selection** | **Prediction modelling method^2^** | **Internal validation**  **[Valid: Y/N]** | **Discrimination^3^** | **Calibration** | **External validation** |
| Morel et al (2020) [186] | USA | Patients admitted to hospital with mental or substance use disorder diagnosis within insurance claims database (11-64) | Readmission within 30 days from index hospital discharge (Transdiagnostic) | Prognostic (course) | 97,688 (15,974) | 261.87 | Ranking-based method (feature importance) | *Extreme gradient boosting*; Elastic net logistic regression | Random split sample [N] | AUC = 0.74 | – | – |
| Morrow et al (2020) [187] | USA | Youth in multi-wave household survey with parent-reported ADHD diagnosis (12.4) | Receipt of psychosocial treatment for ADHD during past 12 months (ADHD) | Diagnostic | 6,630 (2,757) | 4.13 | Available data; feature importance | Classification and regression tree; Ensemble method; *Neural network*; Logistic regression | Cross-validation [N] | AUC = 0.78 | – | Temporal validation using data from later study wave (*n* = 2,013; AUC = 0.72) |
| Na, Cho, et al (2020) [188] | South Korea | Community dwelling adults within national longitudinal survey (46.6) | Significant depressive symptoms for two consecutive years (Depression) | Prognostic (onset) | 6,588 (209) | 8.70 | – | Random forest | Random split sample [N] | AUC = 0.87 | – | – |
| Na, Geem, et al (2020) [189] | South Korea | Children within longitudinal cohort study (4.3) | Persistent oppositionality over 4 consecutive years (ODD) | Prognostic (course) | 1,323 (13) | 1.44 | – | Random forest | Random split sample [N] | AUC = 0.98 | – | – |
| Nelson et al (2012) [190] | Bulgaria, Croatia, Hungary, Poland, Romania, Russia, Slovakia, USA | Placebo-treated patients with MDD drawn from eight duloxetine RCTs (45) | Remission of depressive symptoms 7-8 weeks after randomisation (Depression) | Prognostic (course) | 1,017 (282) | – | Forward selection; ranking-based method | *Logistic regression*; Classification and regression trees | Random split sample [N] | AUC = 0.63 | – | – |
| Nelson et al (2013) [191] | Australia | CHR patients within specialist clinic (18.9) | Transition to psychosis during 15-year follow-up (Psychosis) | Prognostic (onset) | 311 (114) | 2.92 | Univariable analyses; forward and backward selection | Cox proportional hazards regression | – | Sen. = 0.44-0.48  Spe. = 0.83-0.88  PPV = 0.39-0.72  NPV = 0.69-0.88 | – | – |
| Nichols et al (2018) [192] | UK | Matched sample of depressed males in primary care vs controls (age 15-18) | First recorded depression diagnosis (Depression) | Prognostic (onset) | 18,776 (4,702) | 97.96 | Backward selection | Logistic regression | Random split sample [N] | AUC = 0.71 | – | – |
|  |  | Matched sample of depressed males in primary care vs controls (age 19-24) | First recorded depression diagnosis (Depression) | Prognostic (onset) | 69,433 (17,526) | 365.13 | “ | “ | “ | AUC = 0.72 | – | – |
|  |  | Matched sample of depressed females in primary care vs controls (age 15-18) | First recorded depression diagnosis (Depression) | Prognostic (onset) | 46,172 (11,857) | 247.02 | “ | “ | “ | AUC = 0.72 | – | – |
|  |  | Matched sample of depressed females in primary care vs controls (age 19-24) | First recorded depression diagnosis (Depression) | Prognostic (onset) | 125,075 (33,236) | 692.42 | “ | “ | “ | AUC = 0.70 | – | – |
| **Author (Year)** | **Country** | **Study setting (mean age or age range, years)** | **Outcome (Psychiatric domain)** | **Model type** | **Sample size**  **(*N* events)** | **EPV^1^** | **Predictor selection** | **Prediction modelling method^2^** | **Internal validation**  **[Valid: Y/N]** | **Discrimination^3^** | **Calibration** | **External validation** |
| Nie et al (2018) [193] | USA | Outpatients with MDD within antidepressant effectiveness study (n.a.) | Treatment resistance, or failure to reach remission following two adequate trials (Depression) | Predictive | 2,454 (642) | 0.92 | Inbuilt variable selection (variable importance; elastic net) | Ridge logistic regression; Elastic net logistic regression; *Random forest*; *Gradient boosting decision trees*; Extreme gradient boosting | Random split sample [N] | AUC = 0.78 | – | Overlapping features validated in hold-out test set from independent regional centres (*n* = 490; optimal AUC = 0.78), and data from independent clinical trial (*n* = 225; optimal AUC = 0.73) |
| Nieman et al (2014) [194] | Netherlands | Help-seeking CHR individuals (19.9) | Conversion to psychosis during 3-year follow-up (Psychosis) | Prognostic (onset) | 58 (18) | 0.9 | Theory-driven (previous research); stepwise selection | Cox proportional hazards regression | Bootstrapping [N] | AUC = 0.86 | – | – |
| Niemann et al (2020) [195] | Germany | Adult tinnitus patients treated at specialist centre (49.8) | Clinical depression symptoms after 7-day outpatient treatment (Depression) | Prognostic (onset) | 1,490 (777) | 4.2 | Feature selection wrapper | *LASSO logistic regression*; Ridge logistic regression; K-nearest neighbour; Naïve Bayes; Support vector machine; Neural network; Generalized partial least squares; Classification and regression trees; Random forest; Gradient boosted trees | Cross-validation [N] | AUC = 0.87 | – | – |
| Nyberg et al (2016) [196] | Sweden | Parents of individuals who died by suicide between 15-30 years of age, identified using national records (n.a.) | Moderate-to-severe depression 2-6 years after child’s death (Depression) | Diagnostic | 665 (167) | 3.98 | Univariable and multivariable analyses | Logistic regression | Cross-validation [N] | AUC = 0.72 | Calibration plot | – |
| Oh et al (2017) [197] | South Korea | Outpatients with depressive or anxiety disorder diagnosis (35.6) | Past-month suicide attempt (Mixed Affective) | Diagnostic | 573 (39) | 0.95 | Theory-driven (previous research; practical utility and relevance) | Artificial neural network | Random split sample [N] | AUC = 0.93 | – | – |
|  |  | “ | Past-year suicide attempt (Mixed Affective) | Diagnostic | 573 (68) | 1.66 | “ | “ | “ | AUC = 0.87 | – | – |
|  |  | “ | Lifetime history of suicide attempts (Mixed Affective) | Diagnostic | 573 (163) | 3.98 | “ | “ | “ | AUC = 0.89 | – | – |
| **Author (Year)** | **Country** | **Study setting (mean age or age range, years)** | **Outcome (Psychiatric domain)** | **Model type** | **Sample size**  **(*N* events)** | **EPV^1^** | **Predictor selection** | **Prediction modelling method^2^** | **Internal validation**  **[Valid: Y/N]** | **Discrimination^3^** | **Calibration** | **External validation** |
| Oh et al (2019) [198] | USA | Adults within US general-population health survey (n.a.) | Above screening threshold for depression diagnosis (Depression) | Diagnostic | 28,280 (2,242) | 14.28 | Inbuilt variable selection | *Deep learning*; Support vector machine; Logistic regression; K-nearest neighbour; Decision trees | Cross-validation [N] | AUC = 0.91 | – | US-based model validated (i) temporally using later survey waves (optimal AUC = 0.92), and (ii) cross-nationally in South Korean sample using 40 common predictors (*n* = 4,949; optimal AUC = 0.77) |
|  | South Korea | Adults within South Korean general-population health survey (n.a.) | Above screening threshold for depression diagnosis (Depression) | Diagnostic | 4,949 (344) | 1.09 | “ | “ | “ | AUC = 0.89 | – | – |
| Okamoto and Harasawa (2011) [199] | Japan | Community-dwelling older adults (59.8) | Classification as depressed vs non-depressed based on median symptom score (Depression) | Diagnostic | 690 (192) | 17.45 | Univariable analyses | Discriminant function analysis | – | Acc. = 0.78  Sen. = 0.78  Spe. = 0.80 | – | Geographic validation in later study (Cattelani et al, 2019) using independent samples from Ireland (*n* = 3,124; AUC = 0.68) and the UK (*n* = 7,920; AUC = 0.67) |
| Papini et al (2018) [200] | USA | Adults admitted to Level I Trauma Center (46.7) | PTSD status at 3-month follow-up (PTSD) | Prognostic (onset) | 271 (110) | 2.68 | Inbuilt variable selection | Extreme gradient boosting | Cross-validation [Y] | AUC = 0.85 | – | – |
| Parikh et al (2019) [201] | Belgium; Germany; Ireland; Netherlands; USA | Patients diagnosed with ASD and healthy controls (16.8) | ASD diagnosis (ASD) | Diagnostic | 851 (421) | 70.17 | *A priori* based on available data | K-nearest neighbour; Support vector machine; Decision tree; Logistic regression *Neural network*; Random forest; Ensemble method | Cross-validation [Y] | AUC = 0.65 | – | – |
| Passos et al (2016) [202] | USA | Patients with bipolar or major depressive disorder (36.4) | Historical suicide attempt (Mixed Affective) | Diagnostic | 144 (43) | 2.87 | Theory-driven (previous research) | LASSO logistic regression; Support vector machine; *Relevance vector machine* | Cross-validation [N] | AUC = 0.77 | – | – |
| Perez Arribas et al (2018) [203] | UK | Adults with bipolar disorder and healthy controls within longitudinal study (37.5) | Classification of bipolar disorder patients vs healthy controls (Bipolar) | Diagnostic | 99 (48) | – | Available data | Random forest | Random split sample [N] | AUC = 0.91 | – | – |
|  |  | Adults with borderline personality disorder and healthy controls within longitudinal study (35.5) | Classification of borderline personality disorder patients vs healthy controls (BPD) | Diagnostic | 82 (31) | – |  | “ | “ | AUC = 0.98 | – | – |
| **Author (Year)** | **Country** | **Study setting (mean age or age range, years)** | **Outcome (Psychiatric domain)** | **Model type** | **Sample size**  **(*N* events)** | **EPV^1^** | **Predictor selection** | **Prediction modelling method^2^** | **Internal validation**  **[Valid: Y/N]** | **Discrimination^3^** | **Calibration** | **External validation** |
| Perlis (2013) [204] | USA | Outpatients with MDD within antidepressant effectiveness study  (41.3) | Treatment resistance, or failure to reach remission following two adequate trials  (Depression) | Predictive | 2,094 (737) | 15.35 | Theory-driven (practical utility or relevance); Wrapper subset evaluation | *Logistic regression*; Naïve Bayes; Random forest; Support vector machine | Random split sample [N] | AUC = 0.72 | Calibration plot; Hosmer-Lemeshow | – |
| Pradier et al (2021) [205] | USA | Adult outpatients prescribed with antidepressant (48) | Conversion to bipolar diagnosis within 3 months of antidepressant prescription (Bipolar) | Prognostic (onset) | 42,547 (636) | 0.07 | Inbuilt variable selection | LASSO logistic regression; *Random forest* | Random split sample [N] | AUC = 0.80 | Calibration plot | Geographic validation in hold-out independent site (*n* = 25,260; AUC = 0.70) |
| Rabelo-da-Ponte et al (2020) [206] | Brazil | Adults within prospective birth cohort (22) | Bipolar disorder diagnosis at age 22 follow-up (Bipolar) | Prognostic (onset) | 3,778 (255) | 4.81 | Theory-driven (previous research); inbuilt variable selection (elastic net) | Elastic net logistic regression | Random split sample [N] | AUC = 0.82 | – | – |
| Raket et al (2020) [207] | USA | Matched sample of first-episode psychosis patients and random non-psychotic controls within electronic health record (43.5) | 1-year risk of first-episode psychosis (Psychosis) | Prognostic (onset) | 145,720 (72,860) | 14.87 | Available data | Recurrent neural network | Random split sample [N] | AUC = 0.86 | – | Geographic validation in patient subset whose healthcare encounters took place at external providers (*n* = 4,770; AUC = 0.80) |
| Reps et al (2020) [208] | USA | Patients with first-time opioid dispensing, drawn from claims database (48.5) | Opioid use disorder during 1-year follow-up (SUDs) | Prognostic (onset) | 2,897,134 (4,262) | 0.05 | Inbuilt feature selection (LASSO); *a priori* based on subjective evaluation | LASSO logistic regression | Random split sample [N] | AUC = 0.72 | Calibration plot | Geographic validation in three independent claims databases (*n* = 4,540,979, AUC = 0.77; *n* = 579,563, AUC = 0.83; *n* = 630,022, AUC = 0.75) |
| Rezaii et al (2019) [209] | USA | Matched sample of CHR individuals within longitudinal study (21) | Conversion to psychosis during 2-year follow-up (Psychosis) | Prognostic (onset) | 30 (7) | – | Inbuilt variable selection | Latent content analysis | – | Acc. = 0.93  Sen. = 0.86  Spe.= 0.96 | – | Temporal validation using holdout sample from later wave (*n* = 10; Acc. = 0.90) |
| Riecher-Rössler et al (2009) [210] | Switzerland | Outpatients at risk for psychosis within specialised clinic (26.3) | Conversion to psychosis during 7-year follow-up (Psychosis) | Prognostic (onset) | 53 (21) | – | Backward selection | Logistic regression | Cross-validation [N] | AUC = 0.87 | – | – |
| Rocha et al (2021) [211] | Brazil | Children in prospective cohort study (18) | Major depressive disorder diagnosis at age 18-19 years (Depression) | Prognostic (onset) | 2,192 (69) | 1.73 | Theory-driven (previous research; data availability) | Logistic regression with penalised maximum likelihood estimation | Bootstrapping [Y] | AUC = 0.78 | Calibration plot; calibration-in-the-large; calibration slope | Geographic validation using samples from (i) New Zealand (*n* = 739; c = 0.63), (ii) the UK (*n* = 1,144 ; *c* = 0.59), and (iii) in a separate publication, Nepal (Brathwaite et al, 2020: *n* = 126; *c* = 0.73) |
| **Author (Year)** | **Country** | **Study setting (mean age or age range, years)** | **Outcome (Psychiatric domain)** | **Model type** | **Sample size**  **(*N* events)** | **EPV^1^** | **Predictor selection** | **Prediction modelling method^2^** | **Internal validation**  **[Valid: Y/N]** | **Discrimination^3^** | **Calibration** | **External validation** |
| Roglio et al (2020) [212] | Brazil | Male inpatients with cocaine use disorder (34.3) | Lifetime suicide attempt (SUDs) | Diagnostic | 247 (84) | 1.47 | Theory-driven (previous research); inbuilt variable selection (recursive feature elimination) | Random forest | Random split sample [N] | AUC = 0.68 | – | ­– |
|  |  | Female inpatients with cocaine use disorder (31.4) | Lifetime suicide attempt (SUDs) | Diagnostic | 422 (211) | 3.70 | “ | “ | “ | AUC = 0.73 | – | ­– |
| Rosellini, Dussaillant, et al (2018) [213] | Chile | Household survey of individuals living in Chile during 2010 earthquake (50.3) | Probable PTSD diagnosis at follow-up (PTSD) | Prognostic (onset) | 23,907 (3,182) | 47.49 | Theory-driven (previous research); inbuilt variable selection | Super Learner ensemble | Bootstrapping [N] | AUC = 0.79 | – | – |
| Rosellini, Stein, et al (2018) [214] | USA | Four-wave panel survey of soldiers deployed to Afghanistan (n.a.) | Major depressive episode criteria reported for at least 3 months of deployment (Depression) | Prognostic (onset) | 7,081 (395) | 1.45 | Univariable analyses; inbuilt variable selection (LASSO) | Super Learner ensemble | Cross-validation [N] | AUC = 0.88 | – | – |
|  |  | “ | Generalized anxiety disorder criteria reported for at least 3 months of deployment (Anxiety) | Prognostic (onset) | 7,081 (400) | 1.47 | “ | “ | “ | AUC = 0.85 | – | – |
| Rosellini et al (2020) [215] | USA | Adults within longitudinal household survey (46) | Incident occurrence of generalized anxiety disorder since baseline (Anxiety) | Prognostic (onset) | 33,018 (1,126) | 5.29 | Univariable analyses; inbuilt variable selection (LASSO) | Super Learner ensemble | Cross-validation [N] | AUC = 0.80 | Calibration statistics (slope) | – |
|  |  | Adults within longitudinal household survey (46.1) | Incident occurrence of social phobia since baseline (Anxiety) | Prognostic (onset) | 32,714 (648) | 3.04 | “ | “ | “ | AUC = 0.78 | “ | – |
|  |  | Adults within longitudinal household survey (46.1) | Incident occurrence of panic disorder since baseline (Anxiety) | Prognostic (onset) | 32,902 (559) | 2.62 | “ | “ | “ | AUC = 0.79 | “ | – |
|  |  | Adults within longitudinal household survey (46.5) | Incident occurrence of major depressive disorder since baseline (Depression) | Prognostic (onset) | 27,769 (1,708) | 8.02 | “ | “ | “ | AUC = 0.76 | “ | – |
|  |  | Adults within longitudinal household survey (46.5) | Incident occurrence of manic or hypomanic episode since baseline (Bipolar) | Prognostic (onset) | 32,496 (972) | 4.56 | “ | “ | “ | AUC = 0.83 | “ | – |
| Ruhrmann et al (2010) [216] | Finland, Germany, Netherlands, UK | Help-seeking CHR individuals within longitudinal study (23.6) | Conversion to psychosis during 18-month follow-up (Psychosis) | Prognostic (onset) | 183 (37) | 0.42 | Univariable analyses; backward selection | Cox proportional hazards regression | – | AUC = 0.81 | – | Geographic validation in later publication (Haidl et al, 2018: *n* = 235; AUC = 0.77) |
| **Author (Year)** | **Country** | **Study setting (mean age or age range, years)** | **Outcome (Psychiatric domain)** | **Model type** | **Sample size**  **(*N* events)** | **EPV^1^** | **Predictor selection** | **Prediction modelling method^2^** | **Internal validation**  **[Valid: Y/N]** | **Discrimination^3^** | **Calibration** | **External validation** |
| Russo et al (2013) [217] | USA | Hospitalised adult trauma survivors (38.2) | High PTSD symptom levels (PTSD) | Diagnostic | 878 (350) | 4.38 | Univariable analyses | Logistic regression | – | AUC = 0.72 | – | Validated in independent sample of injured inpatients in same centre (*n* = 142; AUC = 0.66) |
|  |  | Longitudinal patient subsample comprising intervention vs usual care conditions (n.a.) | PTSD diagnosis 1 month post-injury (PTSD) | Prognostic (onset) | 207 (n.a.) | – | “ | “ | – | AUC = 0.60 | – | – |
|  |  | Longitudinal patient subsample comprising intervention vs usual care conditions (n.a.) | PTSD diagnosis 6 months post-injury (PTSD) | Prognostic (onset) | 207 (n.a.) | – | “ | “ | – | AUC = 0.65 | – | – |
|  |  | Longitudinal patient subsample comprising intervention vs usual care conditions (n.a.) | PTSD diagnosis 12 months post-injury (PTSD) | Prognostic (onset) | 207 (n.a.) | – | “ | “ | – | AUC = 0.67 | – | – |
| Sau and Bhakta (2017a) [218] | India | Older adult outpatients (66.6) | Current presence of depression (Depression) | Diagnostic | 105 (48) | 4 | Available data | Artificial neural network | Random split sample [N] | AUC = 0.99 | – | – |
| Sau and Bhakta (2017b) [219] | India | Older adult outpatients (68.5) | Current presence of anxiety or depression (Mixed Affective) | Diagnostic | 510 (246) | 12.3 | Correlation-based subset evaluation | Bayesian network; Multilayer perceptron; Naïve Bayes; *Random forest*; Random tree; J48, sequential minimal optimisation; random sub-space; K Star | Cross-validation [N] | AUC = 0.94 | – | Geographic validation using independent outpatient sample (*n* = 110; Acc. = 0.91) |
| Saxe et al (2017) [220] | USA | Hospitalised youth (7-18) | High levels of PTSD symptoms 3 months after discharge (PTSD) | Prognostic (onset) | 163 (11) | 0.19 | Inbuilt variable selection | *Support vector machine*; Random forest; LASSO logistic regression | Cross-validation [Y] | AUC = 0.79 | – | – |
| Schepers et al (2009) [221] | Netherlands | Stroke patients admitted for rehabilitation (56.3) | Depressive symptoms 1 year post-stroke (Depression) | Prognostic (onset) | 131 (33) | 2.54 | Stepwise selection | Logistic regression | – | AUC = 0.83 | – | – |
|  |  | “ | Depressive symptoms 3 years post-stroke (Depression) | Prognostic (onset) | 131 (21) | 1.62 | “ | “ | – | AUC = 0.96 | – | – |
| **Author (Year)** | **Country** | **Study setting (mean age or age range, years)** | **Outcome (Psychiatric domain)** | **Model type** | **Sample size**  **(*N* events)** | **EPV^1^** | **Predictor selection** | **Prediction modelling method^2^** | **Internal validation**  **[Valid: Y/N]** | **Discrimination^3^** | **Calibration** | **External validation** |
| Schultebraucks, Qian, et al (2020) [222] | USA | Active-duty army personnel deployed to Afghanistan (26.4) | Membership of increasing vs resilient PTSD symptom trajectory 90-180 days post-deployment (PTSD) | Prognostic (course) | 473 (43) | 0.38 | Theory-driven (previous research); inbuilt variable selection (variable importance) | Random forest; *Support vector machine* | Random split sample [N] | AUC = 0.87 | – | – |
|  |  | “ | Provisional PTSD diagnosis 90-180 days post-deployment (PTSD) | Prognostic (onset) | 473 (36) | 0.32 | “ | “ | “ | AUC = 0.88 | – | – |
| Schultebraucks, Shalev, et al (2020) [223] | USA | Longitudinal study of adult emergency department patients reporting traumatic event (36.1) | Membership of non-remitting vs resilient PTSD symptom trajectory (PTSD) | Prognostic (course) | 253 (41) | 0.49 | Theory-driven (previous research; data availability) | Ensemble method (Super Learner) | Random split sample [N] | AUC = 0.69 | Calibration plot | Geographic validation using independent prospective cohort of emergency department patients (*n* = 93; AUC = 0.86) |
|  |  | “ | Provisional PTSD diagnosis at 12-month follow-up (PTSD) | Prognostic (onset) | 258 (40) | 0.48 | “ | “ | “ | ­– | “ | Geographic validation using independent prospective cohort of emergency department patients (*n* = 75; AUC = 0.87) |
| Serretti et al (2007) [224] | Italy | Inpatients with major depressive episode within open-label trial (51) | Treatment response following 6-week trial (Mixed Affective) | Prognostic (course) | 116 (87) | 5.8 | Available data | Logistic regression; *Artificial neural network* | Random split sample [N] | AUC = 0.77 | – | – |
| Setyawan et al (2015) [225] | Belgium, France, Germany, Hungary, Italy, Netherlands, Poland, Spain, Sweden, UK | Patients receiving one of two medication treatments for ADHD within RCT (10.7) | Treatment failure (<25% symptom improvement) at 7-week follow-up (ADHD) | Predictive | 101 (76) | – | Inbuilt variable selection (LASSO) | LASSO logistic regression | – | AUC = 0.86 | Hosmer-Lemeshow | – |
| Shalev et al (2019) [226] | Australia, Israel, Japan, Netherlands Switzerland, USA | Recent trauma survivors within acute care settings (39) | PTSD diagnosis at 4-15-month follow-up (PTSD) | Prognostic (onset) | 2,473 (291) | 29.1 | Theory-driven (practical utility or relevance); multivariate significance | Logistic regression | Bootstrapping [N] | AUC = 0.85 | Calibration statistics (slope) | – |
| Silveira Jr et al (2020) [227] | Brazil | Outpatients with anxiety disorder, OCD, PTSD, MDD, bipolar, schizoaffective disorders or schizophrenia (44.1) | High vs low levels of rumination (Transdiagnostic) | Diagnostic | 200 (n.a.) | – | Inbuilt variable selection (recursive feature elimination) | *Support vector machine*; Artificial neural network; Random forest | Cross-validation [N] | AUC = 0.83 | ­– | – |
| Silverstein et al (2016) [228] | USA | Youth from primary care clinic (8.7) | Current ADHD diagnosis (ADHD) | Diagnostic | 156 (64) | 9.14 | Forward selection | Logistic regression | Bootstrapping [N] | AUC = 0.84 | – | – |
| **Author (Year)** | **Country** | **Study setting (mean age or age range, years)** | **Outcome (Psychiatric domain)** | **Model type** | **Sample size**  **(*N* events)** | **EPV^1^** | **Predictor selection** | **Prediction modelling method^2^** | **Internal validation**  **[Valid: Y/N]** | **Discrimination^3^** | **Calibration** | **External validation** |
| Simon et al (2018) [229] | USA | Outpatients recording mental health diagnosis in speciality mental health clinic visit (n.a.) | Probable suicide attempt within 90 days of visit (Transdiagnostic) | Prognostic (course) | 10,275,853 (63,799) | 203.83 | Inbuilt variable selection (LASSO) | LASSO logistic regression | Random split sample [N] | *c* = 0.85 | Predicted vs observed outcome probabilities | – |
|  |  | Outpatients recording mental health diagnosis in speciality mental health clinic visit (n.a.) | Suicide death within 90 days of visit (Transdiagnostic) | Prognostic (course) | 10,275,853 (2,383) | 7.61 | “ | “ | “ | *c* = 0.86 | “ | – |
|  |  | Outpatients recording mental health diagnosis in speciality primary clinic visit (n.a.) | Probable suicide attempt within 90 days of visit (Transdiagnostic) | Prognostic (course) | 9,685,206 (24,990) | 79.84 | “ | “ | “ | *c* = 0.85 | “ | – |
|  |  | Outpatients recording mental health diagnosis in speciality primary clinic visit (n.a.) | Suicide death within 90 days of visit (Transdiagnostic) | Prognostic (course) | 9,685,206 (1,301) | 4.16 | “ | “ | “ | *c* = 0.83 | “ | – |
| Slobodin et al (2020) [230] | Israel | Child outpatients and control subjects who were administered continuous performance test (8.7) | Classification of ADHD group vs typically developing peers (ADHD) | Diagnostic | 458 (213) | 26.63 | Available data | *Random forest;* Neural network | Random split sample, repeated [N] | Acc. = 0.87  Sen. = 0.89  Spe. = 0.84 | – | – |
| Studerus et al (2020) [231] | Switzerland | CHR patients within longitudinal study (25) | Conversion to psychosis during 5-year follow-up (Psychosis) | Prognostic (onset) | 196 (42) | 4.67 | Theory-driven (previous research); Inbuilt variable selection (LASSO) | Bayesian joint modelling | Cross-validation [N] | AUC = 0.71 | Integrated prediction error | – |
| Thompson et al (2011) [232] | Australia | CHR patients within longitudinal study (19.3) | Conversion to psychosis during 1-year follow-up (Psychosis) | Prognostic (onset) | 104 (41) | 8.2 | Theory-driven (previous research); backward selection | Cox proportional hazards regression | – | Sen. = 0.37  Spe. = 0.87  PPV = 0.65  NPV = 0.68 | – | – |
| Tondo et al (2014) [233] | Italy | Adult mood-disorder patients with presenting first episode of major depression (44.7) | Bipolar disorder diagnostic criteria met during 13-year follow-up (Depression) | Prognostic (course) | 2,146 (642) | 42.8 | Univariable analyses; forward selection | Bayesian analysis | – | AUC = 0.72 | – | – |
| Tulloch et al (2016) [234] | UK | Patients discharged from secondary mental health services (39.1) | 90-day readmission following hospital discharge (Transdiagnostic) | Prognostic (course) | 7,891 (1,156) | 33 | Multivariate significance; backward selection | Logistic regression | – | AUC = 0.65 | Hosmer-Lemeshow | – |
| Usta et al (2019) [235] | Turkey | Children diagnosed with ASD within longitudinal study (6) | Improvement in ASD symptoms at 36-month follow-up (ASD) | Prognostic (course) | 185 (144) | 0.57 | Principal component analysis | Naïve Bayes; Generalized linear model; Logistic regression; *Decision tree* | Cross-validation [N] | AUC = 0.71 | – | – |
| **Author (Year)** | **Country** | **Study setting (mean age or age range, years)** | **Outcome (Psychiatric domain)** | **Model type** | **Sample size**  **(*N* events)** | **EPV^1^** | **Predictor selection** | **Prediction modelling method^2^** | **Internal validation**  **[Valid: Y/N]** | **Discrimination^3^** | **Calibration** | **External validation** |
| van Breda et al (2018) [236] | France, Germany, Netherlands, Poland, Spain, Sweden, Switzerland, UK | Patients receiving treatment-as-usual within effectiveness study (n.a.) | Therapy success (significant symptom improvement) at 3-month follow-up (Depression) | Predictive | 276 (45) | 0.15 | Feature similarity; recursive feature elimination | *Random forest*; K-nearest neighbour; General linear model | Random split sample, repeated  [N] | AUC = 0.76 | – | – |
|  |  | Patients receiving blended treatment within effectiveness study (n.a.) | Therapy success (significant symptom improvement) at 3-month follow-up (Depression) | Predictive | 227 (58) | 0.21 | “ | Random forest; K-nearest neighbour; *General linear model* | “ | AUC = 0.78 | – | – |
| van der Aa et al (2016) [237] | Australia Belgium,  Netherlands, | Visually impaired outpatients from rehabilitation centre (n.a.) | Subthreshold depressive symptoms (Depression) | Diagnostic | 873 | – | Multivariate significance | Logistic regression | Cross-validation [N] | AUC = 0.80 | Calibration plot; Hosmer-Lemeshow | Geographic validation using Australian patient sample (*n* = 124; AUC = 0.88) |
| van Heumen et al (2018) [238] | Netherlands | Adult women retrospectively reporting a traumatic birth experience (n.a.) | Postpartum PTSD diagnosis (PTSD) | Diagnostic | 1,599 (278) | 9.27 | Univariable analyses; backward selection | Logistic regression | – | AUC = 0.80 | – | – |
| van Loo et al (2015) [239] | USA | Female twins reporting major depressive episode in previous year (30.7) | Recurrence of major depression during follow-up (Depression) | Prognostic (course) | 194 (101) | 1.25 | Inbuilt variable selection (elastic net) | Elastic net logistic regression | Cross-validation [N] | AUC = 0.79 | Other (Kaplan Meier survival curve) | Geographic validation using independent sample (*n* = 133; AUC = 0.61) |
| van Loo et al (2018) [240] | USA | Twins reporting a past-year major depressive episode (35.3) | Recurrence of major depression during 5-year follow-up (Depression) | Prognostic (course) | 653 (405) | 5.79 | Inbuilt variable selection (elastic net) | Elastic net cox regression | – | AUC = 0.75 | Other (Kaplan Meier survival curve) | – |
|  |  | Male twins reporting a past-year major depressive episode (34.9) | Recurrence of major depression during 5-year follow-up (Depression) | Prognostic(course) | 427 (n.a.) | – | Inbuilt variable selection (elastic net) | “ | Random split sample [N] | AUC = 0.71 | Other (Kaplan Meier survival curve) | Validated in opposite-sex (female) test sample (*n* = 226; AUC = 0.69) |
|  |  | Female twins reporting a past-year major depressive episode (35.9) | Recurrence of major depression during 5-year follow-up (Depression) | Prognostic (course) | 226 (n.a.) | – | Inbuilt variable selection (elastic net) | “ | – | AUC = 0.78 | Other (Kaplan Meier survival curve) | Validated in opposite-sex (male) test sample (*n* = 427; AUC = 0.68) |
| van Voorhees et al (2008) [241] | USA | Adolescents within longitudinal study (15.6) | Onset of depressive episode at 1-year follow-up (Depression) | Prognostic (onset) | 4,373 (122) | 2.35 | Inbuilt variable selection (variable importance) | Classification and regression trees | Random split sample [N] | AUC = 0.78 | – | – |
| Vöhringer et al (2013) [242] | Chile | Adult primary care patients (48.5) | Current (past month) presence of major depressive or manic/hypomanic episode (Mixed Affective) | Diagnostic | 197 (72) | 2 | Theory-driven (prev. research); univariable analyses; backward selection | Logistic regression with shrinkage factor | Cross-validation [N] | AUC = 0.80 | Hosmer-Lemeshow | – |
| **Author (Year)** | **Country** | **Study setting (mean age or age range, years)** | **Outcome (Psychiatric domain)** | **Model type** | **Sample size**  **(*N* events)** | **EPV^1^** | **Predictor selection** | **Prediction modelling method^2^** | **Internal validation**  **[Valid: Y/N]** | **Discrimination^3^** | **Calibration** | **External validation** |
| Vöhringer et al (2016) [243] | Chile | Adult patients from tertiary mood disorder clinic (41) | Bipolar illness triad classification using triad model (Bipolar) | Diagnostic | 260 (118) | 7.38 | Univariable analyses; backward selection | Logistic regression | – | AUC = 0.91 | – | – |
|  |  | “ | Bipolar illness classification using full model (Bipolar) | Diagnostic | 260 (118) | 7.38 | “ | “ | – | AUC = 0.95 | – | – |
|  |  | “ | BPD classification (BPD) | Diagnostic | 260 (52) | 3.25 | “ | “ | – | AUC = 0.67 | – | – |
| Wang et al (2013) [244] | Canada | Male adults within longitudinal survey (46.3) | 4-year risk of major depressive episode (Depression) | Prognostic (onset) | 4,737 (249) | – | Univariable analyses; forward selection | Logistic regression | – | *c* = 0.80 | Calibration plot; Hosmer-Lemeshow test | Temporal validation using participants eligible for inclusion during later study waves (*n* = 3,484; *c* = 0.77) |
|  |  | Female adults within longitudinal survey (48.9) | 4-year risk of major depressive episode (Depression) | Prognostic (onset) | 5,864 (485) | – | “ | “ | – | *c =* 0.77 | “ | Temporal validation using participants eligible for inclusion during later study waves (*n* = 4,418; *c* = 0.74) |
| Wang, Patten, et al (2014) [245] | USA | Adults within longitudinal study with current or lifetime MDE (45.4) | Recurrence of major depression at ~3-year follow-up (Depression) | Prognostic (course) | 1,518 (382) | – | Forward and backward selection | Logistic regression with shrinkage factor | – | *c =* 0.75 | Hosmer-Lemeshow | Geographic validation using subjects from independent regional centres (*n* = 1,195; *c* = 0.72) |
| Wang, Sareen, et al (2014) [246] | USA | Adults within longitudinal study (45.8) | Onset of major depression at ~3-year follow-up (Depression) | Prognostic (onset) | 21,813 (1,363) | – | Univariable analyses; forward and backward selection | Logistic regression with shrinkage factor | – | *c =* 0.75 | Calibration plot; Hosmer-Lemeshow | Geographic validation using participants from an independent census region (*n* = 6,246; *c* = 0.73) |
| Wang et al (2019) [247] | USA | Clinical registry of pregnant women (34.1) | Postpartum depression diagnosis within 12 months of childbirth (Depression) | Prognostic (onset) | 9,980 (769) | 3 | Univariable analyses | *Support vector machine*; Ridge logistic regression; Random forest; Naïve Bayes; Extreme gradient boosting; Decision trees | Cross-validation [N] | AUC = 0.79 | – | – |
| Wang et al (2020) [248] | Canada | Adult patients with schizophrenia spectrum disorder (41.7) | History of physical violence (Psychosis) | Diagnostic | 275 (103) | 3.68 | Theory-driven (previous research) | LASSO logistic regression; Elastic net logistic regression; *Random forest*; Gradient boosted regression trees; Support vector machine; Radial basis function kernels | Cross-validation [N] | AUC = 0.63 | – | ­– |
| **Author (Year)** | **Country** | **Study setting (mean age or age range, years)** | **Outcome (Psychiatric domain)** | **Model type** | **Sample size**  **(*N* events)** | **EPV^1^** | **Predictor selection** | **Prediction modelling method^2^** | **Internal validation**  **[Valid: Y/N]** | **Discrimination^3^** | **Calibration** | **External validation** |
| Wolf et al (2018) [249] | Sweden | Individuals discharged from secure psychiatric hospitals (36) | Violent offending within 12 months of discharge (Transdiagnostic) | Prognostic (course) | 1,817 (159) | 7.95 | Theory-driven (previous research); backward selection | Cox proportional hazards regression | Bootstrapping [N] | c = 0.77 | Calibration plot | – |
|  |  | “ | Violent offending within 24 months of discharge (Transdiagnostic) | Prognostic (course) | 1,583 (249) | 12.45 | “ | “ | “ | c = 0.77 | “ | – |
| Wshah et al (2019) [250] | USA | Patients in Level 1 trauma centre who experienced traumatic event (35) | Likely PTSD diagnosis during 45-day follow-up (PTSD) | Prognostic (onset) | 90 | – | Inbuilt variable selection | *Ensemble classifier*; logistic regression; Naïve Bayes; Support vector machine; Random forest | Cross-validation [N] | AUC = 0.85 | – | – |
| Wu et al (2016) [251] | USA | Euthymic bipolar disorder patients and matched controls (35.6) | Current euthymic bipolar disorder diagnosis (Bipolar) | Diagnostic | 42 (21) | 0.57 | Theory-driven (previous research); inbuilt variable selection (LASSO) | LASSO logistic regression | Cross-validation [N] | AUC = 0.71 | – | – |
| Xu et al (2019) [252] | USA | Elderly adults within longitudinal household survey (n.a.) | Onset of depressive disorder during 2-year follow-up (Depression) | Prognostic (onset) | 5,686 (2,843) | – | Inbuilt variable selection | *Recurrent neural network*; Dynamic Bayesian network;  Multilayer perceptron; Support vector machine | Temporal internal validation [Y] | *c* = 0.87 | – | – |
| Yung et al (2003) [253] | Australia | CHR subjects within specialist clinic (19.1) | Conversion to psychosis during 1-year follow-up (Psychosis) | Prognostic (onset) | 49 (20) | 1.82 | Univariable analyses | Cox proportional hazards regression | – | Sen. = 0.65-0.90  Spe. = 0.84-0.93  PPV = 0.60-0.87  NPV = 0.77-0.97 | – | – |
| Yung et al (2004) [254] | Australia | CHR subjects within specialist clinic (19.4) | Conversion to psychosis during 1-year follow-up (Psychosis) | Prognostic (onset) | 104 (36) | 1.8 | Univariable analyses | Cox proportional hazards regression | – | Sen. = 0.60  Spe. = 0.93  PPV = 0.81  NPV = 0.82 | – | – |
| Zhang, Xu, Tang, et al (2019) [255] | China | CHR patients (20.5) | Conversion to psychosis during follow-up (Psychosis) | Prognostic (onset) | 349 (83) | 3.45 | Univariable analyses | Logistic regression | – | AUC = 0.74 | – | Validated in independent CHR subjects (i) within the same study (*n* = 91; AUC = 0.80) and (ii) in a separate publication (Osborne & Mittal, 2019: *n* = 62; AUC = 0.65) |
| **Author (Year)** | **Country** | **Study setting (mean age or age range, years)** | **Outcome (Psychiatric domain)** | **Model type** | **Sample size**  **(*N* events)** | **EPV^1^** | **Predictor selection** | **Prediction modelling method^2^** | **Internal validation**  **[Valid: Y/N]** | **Discrimination^3^** | **Calibration** | **External validation** |
| Zhang, Xu, Li, et al (2019) [256] | China | CHR individuals (19.1) | Conversion to psychosis during 2-year follow-up (Psychosis) | Prognostic (onset) | 196 (51) | 3.64 | Factor analysis | Cox proportional hazards regression | Bootstrapping [N] | AUC = 0.78 | Hosmer-Lemeshow | Temporal validation using independent CHR subjects from later study phase (*n* = 93; AUC = 0.80) |
| Zhang et al (2020) [257] | China | Pregnant women drawn from maternity and child care centres (28.6) | Depression at 6 weeks postpartum (Depression) | Prognostic (onset) | 508 (173) | 3.33 | Theory-driven (clinical utility or relevance); random forest feature selection | Random forest; *Support vector machine* | Random split sample [N] | AUC = 0.78 | – | – |
| Ziermans et al (2014) [258] | Netherlands | Help-seeking CHR adolescents (15.2) | Conversion to psychosis during 6-year follow-up (Psychosis) | Prognostic (onset) | 43 (10) | – | Backward selection | Logistic regression | – | AUC = 0.82 | – | – |
| Zuithoff et al (2009) [259] | Netherlands | Adult primary care patients (44.7) | Presence of major depressive disorder at baseline (Depression) | Diagnostic | 1,046 (157) | 9.81 | Theory-driven (previous research; clinical reasoning); backward selection | Logistic regression | Bootstrapping [N] | *c* = 0.80 | Calibration plot | – |
| ***External validation only*** | | | | | | | | | | | | |
| Brathwaite et al (2020) [260] | Nepal | Former child soldiers and matched civilian adolescents (11-18) | Clinically relevant depressive symptoms at follow-up when aged 18 or older (Depression) | Prognostic (onset) | 126 (25) | <100 |  |  |  | AUC = 0.73 | Calibration plot; calibration statistics (intercept, slope |  |
| Carrión et al (2016) [261] | USA | CHR youth from multi-site clinical trial (16.6) | Conversion to psychosis during 2-year follow-up (Psychosis) | Prognostic (onset) | 176 (12) | <100 | ­ |  |  | AUC *=* 0.79 | – |  |
| Fusar-Poli, Werbeloff, et al (2019) [262] | UK | Clinical registry of non-psychotic patients (40.9) | 6-year risk of developing psychosis (Psychosis) | Prognostic (onset) | 13,702 (490) | >100 |  | ­ |  | *c* = 0.73 | Calibration statistics (slope) |  |
| King et al (2013) [263] | Portugal, Slovenia, Spain, UK | Primary care patients in prospective study without major depressive episode during first 12 months (18-75) | Onset of major depression between 12- and 24-month follow-up (Depression) | Prognostic (onset) | 2,440 (78) | <100 |  |  |  | *c* = 0.73 | Calibration plot |  |
| Negatsch et al (2019) [264] | Germany | Patients in prison hospital diagnoses with schizophrenia-spectrum or bipolar disorder (32) | Violent behaviour during treatment episode (Psychosis/Bipolar) | Diagnostic | 474 (191) | >100 | ­ |  |  | AUC = 0.72 | Hosmer-Lemeshow |  |
| Nigatu, Liu, and Wang (2016) [265] | USA | Adults within general-population longitudinal study (43.8) | New onset of major depressive episode during 3-year follow-up (Depression) | Prognostic (onset) | 24,311 (1,945) | >100 |  |  |  | *c* = 0.71 | Calibration plot; Hosmer-Lemeshow |  |
| **Author (Year)** | **Country** | **Study setting (mean age or age range, years)** | **Outcome (Psychiatric domain)** | **Model type** | **Sample size**  **(*N* events)** | **EPV^1^** | **Predictor selection** | **Prediction modelling method^2^** | **Internal validation**  **[Valid: Y/N]** | **Discrimination^3^** | **Calibration** | **External validation** |
| Nigatu and Wang (2019) [266] | USA | Adults within general-population longitudinal study (18-75) | New onset of generalized anxiety or panic syndrome during 2-year follow-up (Anxiety) | Prognostic (onset) | 24,626 (1,379) | >100 |  |  |  | *c* = 0.62 | Calibration plot; Hosmer-Lemeshow |  |
| Osborne and Mittal (2019) [267] | USA | CHR youth within longitudinal study (18.6) | Conversion to psychosis during 2-year follow-up (Psychosis) | Prognostic (onset) | 62 (7) | <100 |  |  |  | AUC = 0.71 (NAPLS-2); 0.65 (SIPS-RC) | – |  |

Abbreviations: Acc., accuracy; ADHD, attention-deficit/hyperactivity disorder; ASD, autism spectrum disorder; AUC, area-under-the-[ROC]curve; BAC, balanced accuracy; BPD, borderline personality disorder; BPSD, bipolar spectrum disorder; *c*, concordance index; CHR, clinical high-risk; EPV, events per variable; ID, intellectual disability; LASSO, least absolute shrinkage and selection operator; MDD, major depressive disorder; n.a., not available; NPV, negative predictive value; OCD, obsessive-compulsive disorder; PPV, positive predictive value; PTSD, post-traumatic stress disorder; RCT, randomised control trial; Sen., sensitivity; Spe., specificity; SUDs, substance use disorders.

^1^ All values are approximate EPV estimates, calculated using the best available information for the number of events and candidate predictors provided within each study. Additionally, for external validation studies, we report whether or not there were at least 100 events in the validation sample, as this is the recommended criterion for evaluating EPV in these studies

^2^ Where several statistical methods were used to develop prediction models in a single study, the modelling method associated with the strongest performance estimates, or specified by the study authors as the preferred modelling approach, is reported in italics

^3^ Where both an apparent and internally-validated *c*-index or AUC was reported within a single study, the internally-validated estimate is recorded here (these can be identified by cross-referencing the ‘Internal validation technique’ column)

^4^ As this study sample combined both psychotic and bipolar I symptoms, the final diagnostic classification (Psychosis) was based on the symptoms with the highest prevalence in the sample.

^5^ The model first developed by Fusar-Poli et al. [107] was also subject to decision-curve analysis to estimate net benefit, as well as two feasibility implementation studies that have since been published [268,269].

**Supplementary Table S3.** Quality assessment (risk of bias) ratings for reviewed studies

| **Study** | **Domain** | | | | |
| --- | --- | --- | --- | --- | --- |
|  | Participants | Predictors | Outcome | Analysis | **Overall** |
| **MODEL DEVELOPMENT ANALYSES** | | | | | |
| Abdullah-Koolmees et al (2018) [40] | Low | High | Low | High | **High** |
| Acion et al (2017) [41] | High | Low | Low | High | **High** |
| Addington et al (2010) [42] {1} | Low | Low | Low | High | **High** |
| Addington et al (2010) [42] {2} | Low | Low | Low | High | **High** |
| Addington et al (2010) [42] {3} | Low | Low | Low | High | **High** |
| Addington et al (2017) [43] | Low | Low | Low | High | **High** |
| Agne et al (2020) [44] | Low | High | High | High | **High** |
| Ahn et al (2016) [45] | Low | Unclear | Unclear | High | **High** |
| Ahn and Vassileva (2016) [46] {1} | Low | Unclear | Unclear | High | **High** |
| Ahn and Vassileva (2016) [46] {2} | Low | Unclear | Unclear | High | **High** |
| Amminger et al (2015) [47] {1} | Low | Low | Low | High | **High** |
| Amminger et al (2015) [47] {2} | Low | Low | Low | High | **High** |
| Andrews et al (2017) [48] | Low | Low | Low | High | **High** |
| Askland et al (2015) [49] | Low | Low | Low | High | **High** |
| Bares et al (2017) [50] | Low | High | Low | High | **High** |
| Barnes et al (2019) [51] | Low | Low | Low | High | **High** |
| Barros et al (2017) [52] | Low | Unclear | Unclear | High | **High** |
| Bauer et al (2019) [53] {1} | High | Unclear | Unclear | High | **High** |
| Bauer et al (2019) [53] {2} | High | Unclear | Unclear | High | **High** |
| Bellón et al (2011) [54] | Low | Low | Low | High | **High** |
| Birmaher et al (2018) [55] | Low | Low | Low | High | **High** |
| Birmaher et al (2020) [56] | Low | Low | Low | High | **High** |
| Birnbaum et al (2019) [57] | Low | High | Low | High | **High** |
| Birnbaum et al (2020) [58] {1} | Low | Low | Low | High | **High** |
| Birnbaum et al (2020) [58] {2} | Low | Low | Low | High | **High** |
| Bishop-Fitzpatrick et al (2018) [59] | Low | Low | Low | High | **High** |
| Bledsoe et al (2016) [60] | Low | High | Low | High | **High** |
| Boer et al (2019) [61] {1} | Low | Low | Low | High | **High** |
| Boer et al (2019) [61] {2} | Low | Low | Low | High | **High** |
| Bokma et al (2020) [62] | Low | Low | Low | High | **High** |
| Boscarino et al (2011) [63] | Low | Low | Low | High | **High** |
| Breen et al (2019) [64] | High | High | High | High | **High** |
| Browning et al (2019) [65] | Low | Low | Low | High | **High** |
| Bussu et al (2018) [66] | Low | Low | Low | High | **High** |
| Cannon et al (2008) [67] | Low | High | Low | High | **High** |
| Cannon et al (2016) [68] | Low | Low | Low | Low | **Low** |
| Carrión et al (2013) [69] {1} | Low | Low | Low | High | **High** |
| Carrión et al (2013) [69] {2} | Low | Low | Low | High | **High** |
| Carter et al (2002) [70] | High | Low | Low | High | **High** |
| Cattelani et al (2019) [71] {1} | Low | Low | Low | Unclear | **Unclear** |
| Cattelani et al (2019) [71] {2} | Low | Low | Low | Unclear | **Unclear** |
| Cattelani et al (2019) [71] {3} | Low | Low | Low | Unclear | **Unclear** |
| Caye et al (2020) [72] | Low | Low | Low | Low | **Low** |
| Cearns et al (2019) [73] | Low | Low | Low | High | **High** |
| Cepeda et al (2018) [74] | Low | Low | Low | High | **High** |
| Chang et al (2006) [75] {1} | Low | Low | Low | High | **High** |
| Chang et al (2006) [75] {2} | Low | Low | Low | High | **High** |
| Chekroud et al (2016) [76] | Low | Low | Low | High | **High** |
| Chekroud et al (2018) [77] | Low | High | High | Unclear | **High** |
| Cho et al (2019) [78] {1} | Low | Unclear | High | High | **High** |
| Cho et al (2019) [78] {2} | Low | Unclear | High | High | **High** |
| Cho et al (2019) [78] {3} | Low | Unclear | High | High | **High** |
| Cho et al (2019) [78] {4} | Low | Unclear | High | High | **High** |
| Choi et al (2018) [79] | Low | Unclear | Unclear | Unclear | **Unclear** |
| Chondros et al (2018) [80] | Low | Low | Low | High | **High** |
| Christensen et al (2009) [81] | Low | Low | Low | High | **High** |
| Chua et al (2019) [82] | High | Unclear | Low | High | **High** |
| Chung et al (2019) [83] | Low | Low | Low | High | **High** |
| Ciarleglio et al (2019) [84] | Low | Low | Low | High | **High** |
| Clark et al (2016) [85] | Low | Low | Low | High | **High** |
| Connor et al (2007) [86] {1} | Low | Low | Low | High | **High** |
| Connor et al (2007) [86] {2} | Low | Low | Low | High | **High** |
| Corcoran et al (2018) [87] | Low | Unclear | Low | High | **High** |
| Cornblatt et al (2015) [88] | High | Low | Low | High | **High** |
| Crippa et al (2017) [89] | High | Unclear | Low | High | **High** |
| Davidson et al (1999) [90] | Low | Low | Low | High | **High** |
| **Study** | **Domain** | | | | |
|  | Participants | Predictors | Outcome | Analysis | **Overall** |
| de Man-van Ginkel et al (2013) [91] | Low | Low | Low | High | **High** |
| Delgadillo et al (2017) [92] {1} | Low | Low | Low | High | **High** |
| Delgadillo et al (2017) [92] {2} | Low | Low | Low | High | **High** |
| Delgadillo et al (2020) [93] {1} | Low | Low | Low | Unclear | **Unclear** |
| Delgadillo et al (2020) [93] {2} | Low | Low | Low | High | **High** |
| Demetriou et al (2020) [94] {1} | High | Unclear | Unclear | High | **High** |
| Demetriou et al (2020) [94] {2} | High | Unclear | Unclear | High | **High** |
| Demetriou et al (2020) [94] {3} | High | Unclear | Unclear | High | **High** |
| Demetriou et al (2020) [94] {4} | High | Unclear | Unclear | High | **High** |
| Dennis et al (2004) [95] | Low | Low | Low | High | **High** |
| Denys et al (2003) [96] | Low | Low | Low | High | **High** |
| Dinga et al (2018) [97] | Low | Low | Low | High | **High** |
| Dipnall et al (2017) [98] | Low | Low | Low | High | **High** |
| Edgcomb et al (2019) [99] | Low | Low | Low | High | **High** |
| Fazel et al (2017) [100] | Low | Low | Low | Low | **Low** |
| Fazel et al (2019) [101] | Low | Low | Low | Low | **Low** |
| Flygare et al (2020) [102] {1} | Low | Low | Low | High | **High** |
| Flygare et al (2020) [102] {2} | Low | Low | Low | High | **High** |
| Flygare et al (2020) [102] {3} | Low | Low | Low | High | **High** |
| Flygare et al (2020) [102] {4} | Low | Low | Low | High | **High** |
| Fond et al (2019) [103] | Low | Low | Low | High | **High** |
| Francesconi et al (2017) [104] | Low | Low | Low | High | **High** |
| Furukawa et al (2019) [105] {1} | Low | Low | Low | High | **High** |
| Furukawa et al (2019) [105] {2} | Low | Low | Low | High | **High** |
| Fusar-Poli et al (2016) [106] | Low | Low | Low | High | **High** |
| Fusar-Poli et al (2017) [107] | Low | Low | Low | Unclear | **Unclear** |
| Fusar-Poli, Davies, et al (2019) [108] | Low | Low | Low | Unclear | **Unclear** |
| Galatzer-Levy et al (2014) [109] | Low | Low | Low | High | **High** |
| Galatzer-Levy et al (2017) [110] | Low | Low | Low | High | **High** |
| Gan et al (2011) [111] {1} | Low | Low | Low | High | **High** |
| Gan et al (2011) [111] {2} | Low | Low | Low | High | **High** |
| Gan et al (2011) [111] {3} | Low | Low | Low | High | **High** |
| Ge, Jiang, et al (2020) [112] | Low | Low | Low | High | **High** |
| Ge, Li, et al (2020) [113] | Low | Low | Low | High | **High** |
| Gilman et al (2012) [114] | Low | Low | Low | High | **High** |
| Gonzalez-Gutierrez et al (2016) [115] | Low | Low | Low | High | **High** |
| Gu et al (2020) [116] | Low | High | Low | High | **High** |
| Hafeman et al (2017) [117] | Low | Low | Low | High | **High** |
| Haidl et al (2018) [118] | Low | Low | Low | High | **High** |
| Han, Fang, et al (2020) [119] | High | Unclear | Unclear | High | **High** |
| Han, Lee, et al (2020)[120] | Low | Low | Unclear | High | **High** |
| Han, Tomasik, et al (2020) [121] | High | High | Unclear | High | **High** |
| Hariman et al (2020) [122] | Low | Low | Low | High | **High** |
| Harrington et al (2019) [123] | Low | Low | Low | High | **High** |
| Hatton et al (2019) [124] | Low | Low | Low | High | **High** |
| Haynos et al (2020) [125] {1} | Low | Low | Low | High | **High** |
| Haynos et al (2020) [125] {2} | Low | Low | Low | High | **High** |
| Hengartner et al (2017) [126] | High | Low | Low | High | **High** |
| Hettige et al (2017) [127] | High | Low | Low | High | **High** |
| Higginson and Priest (1996) [128] | Low | Low | Unclear | High | **High** |
| Hilbert et al (2020) [129] | Low | High | Low | High | **High** |
| Hirschfeld et al (1998) [130] | Low | Low | Low | High | **High** |
| Hoogendoorn et al (2017) [131] | Low | High | Low | High | **High** |
| Huang et al (2010) [132] | Low | High | Low | High | **High** |
| Huang et al (2014) [133] {1} | Low | Low | Low | High | **High** |
| Huang et al (2014) [133] {2} | Low | Low | Low | High | **High** |
| Huang et al (2014) [133] {3} | Low | Low | Low | High | **High** |
| Huang et al (2014) [133] {4} | Low | Low | Low | High | **High** |
| Hughes et al (2020)[134] | Low | Low | Unclear | High | **High** |
| Iniesta et al (2016) [135] {1} | Low | Low | Low | High | **High** |
| Iniesta et al (2016) [135] {2} | Low | Low | Low | High | **High** |
| Iniesta et al (2016) [135] {3} | Low | Low | Low | High | **High** |
| Ising et al (2016) [136] | Low | Low | Low | High | **High** |
| Jimenez-Serrano et al (2015) [137] | Low | Low | Low | High | **High** |
| Jin, Wu, and Di Capua (2015) [138] | Low | Low | Unclear | High | **High** |
| Jin, Wu, Vidyanti, et al (2015) [139] {1} | Low | Low | Low | High | **High** |
| Jin, Wu, Vidyanti, et al (2015) [139] {2} | Low | Low | Low | High | **High** |
| Jin, Wu, Vidyanti, et al (2015) [139] {3} | Low | Low | Low | High | **High** |
| Jing et al (2020) [140] | Low | Low | Low | High | **High** |
| **Study** | **Domain** | | | | |
|  | Participants | Predictors | Outcome | Analysis | **Overall** |
| Kajiwara et al (2016) [141] | Low | Low | Low | High | **High** |
| Karstoft, Galatzer-Levy et al (2015) [142] | Low | Low | High | Unclear | **High** |
| Karstoft, Statnikov et al (2015) [143] {1} | Low | Low | High | High | **High** |
| Karstoft, Statnikov et al (2015) [143] {2} | Low | Unclear | High | High | **High** |
| Kautzky et al (2017) [144] {1} | Low | Low | Low | High | **High** |
| Kautzky et al (2017) [144] {2} | Low | Low | Low | High | **High** |
| Kautzky et al (2018) [145] | Low | High | Low | High | **High** |
| Kautzky et al (2019) [146] | Low | Low | Low | High | **High** |
| Kendler et al (2019) [147] | Low | Low | Low | High | **High** |
| Kessler et al (2014) [148] | Low | High | Low | Unclear | **High** |
| Kessler et al (2015) [149] | Low | Low | Low | High | **High** |
| Kessler et al (2016) [150] {1} | Low | High | High | High | **High** |
| Kessler et al (2016) [150] {2} | Low | High | High | High | **High** |
| Kessler et al (2016) [150] {3} | Low | High | High | High | **High** |
| Kessler et al (2016) [150] {4} | Low | High | High | High | **High** |
| Kessler et al (2016) [150] {5} | Low | High | High | High | **High** |
| Kim et al (2015) [151] | Low | Low | Low | High | **High** |
| Kim et al (2019) [152] | High | Low | Low | High | **High** |
| King et al (2008) [153] | Low | Low | Low | High | **High** |
| King, Bottomley, et al (2011) [154] {1} | Low | Low | Low | High | **High** |
| King, Bottomley, et al (2011) [154] {2} | Low | Low | Low | High | **High** |
| King, Bottomley, et al (2011) [154] {3} | Low | Low | Low | High | **High** |
| King, Marston, et al (2011) [155] | Low | Low | Low | High | **High** |
| Kirchebner et al (2020) [156] {1} | Low | Low | Low | High | **High** |
| Kirchebner et al (2020) [156] {2} | Low | Low | Low | High | **High** |
| Klein et al (2018) [157] | Low | Low | Low | High | **High** |
| Kotlicka-Antczak et al (2019) [158] | Low | Low | Low | Low | **Low** |
| Koutsouleris et al (2012) [159] | High | Low | Low | High | **High** |
| Koutsouleris et al (2016) [160] {1} | Low | Low | Low | High | **High** |
| Koutsouleris et al (2016) [160] {2} | Low | Low | Low | High | **High** |
| Koutsouleris et al (2018) [161] {1} | High | Low | Low | Unclear | **High** |
| Koutsouleris et al (2018) [161] {2} | High | Low | Low | Unclear | **High** |
| Koutsouleris et al (2018) [161] {3} | High | Low | Low | Unclear | **High** |
| Koutsouleris et al (2018) [161] {4} | High | Low | Low | Unclear | **High** |
| Kwakernaak et al (2020) [162] {1} | Low | Low | Low | High | **High** |
| Kwakernaak et al (2020) [162] {2} | Low | Low | Low | High | **High** |
| Leightley et al (2019) [163] | Low | Low | Low | High | **High** |
| Leighton, Krishnadas, et al (2019) [164] {1} | Low | Low | Low | High | **High** |
| Leighton, Krishnadas, et al (2019) [164] {2} | Low | Low | Low | High | **High** |
| Leighton, Krishnadas, et al (2019) [164] {3} | Low | Low | Low | High | **High** |
| Leighton, Upthegrove, et al (2019) [165] {1} | Low | Low | Low | High | **High** |
| Leighton, Upthegrove, et al (2019) [165] {2} | Low | Low | Low | High | **High** |
| Leighton, Upthegrove, et al (2019) [165] {3} | Low | Low | Low | High | **High** |
| Leighton, Upthegrove, et al (2019) [165] {4} | Low | Low | Low | High | **High** |
| Lencz et al (2006) [166] | Low | Low | Low | High | **High** |
| Lenhard et al (2018) [167] | Low | Low | Low | High | **High** |
| Lerthattasilp et al (2020) [168] | High | Low | Low | High | **High** |
| Levin et al (2005) [169] | Low | Low | Low | High | **High** |
| Lewis et al (2019) [170] | Low | Low | Low | High | **High** |
| Librenza-Garcia et al (2020) [171] {1} | Low | Low | Low | High | **High** |
| Librenza-Garcia et al (2020) [171] {2} | Low | Low | Low | High | **High** |
| Librenza-Garcia et al (2020) [171] {3} | Low | Low | Low | High | **High** |
| Lin et al (2007) [172] | Low | Low | Low | High | **High** |
| Lin et al (2011) [173] | Low | Low | Low | High | **High** |
| Lin et al (2012) [174] | Low | Low | Low | High | **High** |
| Lin et al (2018) [175] {1} | Low | Unclear | Low | High | **High** |
| Lin et al (2018) [175] {2} | Low | Unclear | Low | High | **High** |
| Liu et al (2015) [176] | Low | Low | Low | High | **High** |
| Liu et al (2017) [177] | Low | Low | Low | High | **High** |
| Lorenzo-Luaces et al (2017) [178] | Low | Low | Low | Unclear | **Unclear** |
| Lorimer et al (2021) [179] | Low | Low | Low | High | **High** |
| Maarsingh et al (2011) [180] | Low | Low | Low | High | **High** |
| Maarsingh et al (2018) [181] | Low | Low | Low | High | **High** |
| Mak et al (2011) [182] | High | High | Low | High | **High** |
| Mason et al (2004) [183] | Low | Low | Low | High | **High** |
| Mechelli et al (2017) [184] {1} | Low | Low | Low | High | **High** |
| Mechelli et al (2017) [184] {2} | Low | Low | Low | High | **High** |
| Meehan et al (2020) [185] {1} | Low | Low | Low | High | **High** |
| Meehan et al (2020) [185] {2} | Low | Low | Low | High | **High** |
| **Study** | **Domain** | | | | |
|  | Participants | Predictors | Outcome | Analysis | **Overall** |
| Meehan et al (2020) [185] {3} | Low | Low | Low | High | **High** |
| Morel et al (2020) [186] | Low | Low | Low | High | **High** |
| Morrow et al (2020) [187] | Low | Unclear | Unclear | High | **High** |
| Na, Cho, et al (2020) [188] | Low | Low | Low | High | **High** |
| Na, Geem, et al (2020) [189] | Low | Low | High | High | **High** |
| Nelson et al (2012) [190] | Low | High | Low | High | **High** |
| Nelson et al (2013) [191] | Low | Low | Low | High | **High** |
| Nichols et al (2018) [192] {1} | Low | Low | Low | High | **High** |
| Nichols et al (2018) [192] {2} | Low | Low | Low | High | **High** |
| Nichols et al (2018) [192] {3} | Low | Low | Low | High | **High** |
| Nichols et al (2018) [192] {4} | Low | Low | Low | High | **High** |
| Nie et al (2018) [193] | Low | Low | Low | High | **High** |
| Nieman et al (2014) [194] | Low | Low | Low | High | **High** |
| Niemann et al (2020) [195] | Low | Low | Low | High | **High** |
| Nyberg et al (2016) [196] | Low | Low | Low | High | **High** |
| Oh et al (2017) [197] {1} | Low | High | Unclear | High | **High** |
| Oh et al (2017) [197] {2} | Low | High | Unclear | High | **High** |
| Oh et al (2017) [197] {3} | Low | High | Unclear | High | **High** |
| Oh et al (2019) [198] {1} | Low | Unclear | Low | High | **High** |
| Oh et al (2019) [198] {2} | Low | Unclear | Low | High | **High** |
| Okamoto and Harasawa (2011) [199] | Low | Low | Low | High | **High** |
| Papini et al (2018) [200] | Low | Low | Low | High | **High** |
| Parikh et al (2019) [201] | High | Unclear | Unclear | High | **High** |
| Passos et al (2016) [202] | Low | High | High | High | **High** |
| Perez Arribas et al (2018) [203] {1} | High | Low | Low | High | **High** |
| Perez Arribas et al (2018) [203] {2} | High | Low | Low | High | **High** |
| Perlis (2013) [204] | Low | Low | Low | High | **High** |
| Pradier et al (2021) [205] | Low | Low | Low | High | **High** |
| Rabelo-da-Ponte et al (2020) [206] | Low | Low | Low | High | **High** |
| Raket et al (2020) [207] | High | Low | Low | High | **High** |
| Reps et al (2020) [208] | Low | Low | Low | High | **High** |
| Rezaii et al (2019) [209] | Low | High | Low | High | **High** |
| Riecher-Rössler et al (2009) [210] | Low | Low | Low | Unclear | **Unclear** |
| Rocha et al (2021) [211] | Low | Low | Low | High | **High** |
| Roglio et al (2020) [212] {1} | Low | Low | High | High | **High** |
| Roglio et al (2020) [212] {2} | Low | Low | High | High | **High** |
| Rosellini, Dussaillant, et al (2018) [213] | Low | Low | Low | Unclear | **Unclear** |
| Rosellini, Stein, et al (2018) [214] {1} | Low | Low | Low | High | **High** |
| Rosellini, Stein, et al (2018) [214] {2} | Low | Low | Low | High | **High** |
| Rosellini et al (2020) [215] {1} | Low | Low | Low | High | **High** |
| Rosellini et al (2020) [215] {2} | Low | Low | Low | High | **High** |
| Rosellini et al (2020) [215] {3} | Low | Low | Low | High | **High** |
| Rosellini et al (2020) [215] {4} | Low | Low | Low | High | **High** |
| Rosellini et al (2020) [215] {5} | Low | Low | Low | High | **High** |
| Ruhrmann et al (2010) [216] | Low | Low | Low | High | **High** |
| Russo et al (2013) [217] {1} | Low | Low | Low | High | **High** |
| Russo et al (2013) [217] {2} | Low | Low | Low | High | **High** |
| Russo et al (2013) [217] {3} | Low | Low | Low | High | **High** |
| Russo et al (2013) [217] {4} | Low | Low | Low | High | **High** |
| Sau and Bhakta (2017a) [218] | Low | Low | Low | High | **High** |
| Sau and Bhakta (2017b) [219] | Low | Unclear | Low | High | **High** |
| Saxe et al (2017) [220] | Low | Low | Low | High | **High** |
| Schepers et al (2009) [221] {1} | Low | Low | Low | High | **High** |
| Schepers et al (2009) [221] {2} | Low | Low | Low | High | **High** |
| Schultebraucks, Qian, et al (2020) [222] {1} | Low | High | Low | High | **High** |
| Schultebraucks, Qian, et al (2020) [222] {2} | Low | High | Low | High | **High** |
| Schultebraucks, Shalev, et al (2020) [223] {1} | Low | Low | Low | High | **High** |
| Schultebraucks, Shalev, et al (2020) [223] {2} | Low | Low | Low | High | **High** |
| Serretti et al (2007) [224] | Low | Low | Low | High | **High** |
| Setyawan et al (2015) [225] | Low | Low | Low | High | **High** |
| Shalev et al (2019) [226] | Low | Low | Low | High | **High** |
| Silveira Jr et al (2020) [227] | High | High | Unclear | High | **High** |
| Silverstein et al (2016) [228] | Low | Low | High | High | **High** |
| Simon et al (2018) [229] {1} | Low | Low | Low | High | **High** |
| Simon et al (2018) [229] {2} | Low | Low | Low | High | **High** |
| Simon et al (2018) [229] {3} | Low | Low | Low | High | **High** |
| Simon et al (2018) [229] {4} | Low | Low | Low | High | **High** |
| Slobodin et al (2020) [230] | Low | Unclear | Low | High | **High** |
| Studerus et al (2020) [231] | Low | Low | Low | High | **High** |
| **Study** | **Domain** | | | | |
|  | Participants | Predictors | Outcome | Analysis | **Overall** |
| Thompson et al (2011) [232] | Low | Low | Low | High | **High** |
| Tondo et al (2014) [233] | Low | Low | Low | High | **High** |
| Tulloch et al (2016) [234] | Low | Low | Low | High | **High** |
| Usta et al (2019) [235] | Low | Low | Low | High | **High** |
| van Breda et al (2018) [236] {1} | Low | Low | Low | High | **High** |
| van Breda et al (2018) [236] {2} | Low | Low | Low | High | **High** |
| van der Aa et al (2016) [237] | Low | Unclear | Low | Unclear | **Unclear** |
| van Heumen et al (2018) [238] | Low | Low | Low | High | **High** |
| van Loo et al (2015) [239] | Low | Low | Low | High | **High** |
| van Loo et al (2018) [240] {1} | Low | Low | Low | High | **High** |
| van Loo et al (2018) [240] {2} | Low | Low | Low | High | **High** |
| van Loo et al (2018) [240] {3} | Low | Low | Low | High | **High** |
| van Voorhees et al (2008) [241] | Low | Low | Low | High | **High** |
| Vöhringer et al (2013) [242] | Low | Low | High | High | **High** |
| Vöhringer et al (2016) [243] {1} | Low | Low | Low | High | **High** |
| Vöhringer et al (2016) [243] {2} | Low | Low | Low | High | **High** |
| Vöhringer et al (2016) [243] {3} | Low | Low | Low | High | **High** |
| Wang et al (2013) [244] {1} | Low | Low | Low | High | **High** |
| Wang et al (2013) [244] {2} | Low | Low | Low | High | **High** |
| Wang, Patten, et al (2014) [245] | Low | Low | Low | High | **High** |
| Wang, Sareen, et al (2014) [246] | Low | Low | Low | High | **High** |
| Wang et al (2019) [247] | Low | Low | Low | High | **High** |
| Wang et al (2020) [248] | Low | Low | High | High | **High** |
| Wolf et al (2018) [249] {1} | Low | Low | Low | High | **High** |
| Wolf et al (2018) [249] {2} | Low | Low | Low | High | **High** |
| Wshah et al (2019) [250] | Low | Low | Low | Unclear | **Unclear** |
| Wu et al (2016) [251] | High | Unclear | Low | High | **High** |
| Xu et al (2019) [252] | High | Low | Low | Unclear | **High** |
| Yung et al (2003) [253] | Low | Low | Low | High | **High** |
| Yung et al (2004) [254] | Low | Low | Low | High | **High** |
| Zhang, Xu, Tang, et al (2019) [255] | High | Low | Low | High | **High** |
| Zhang, Xu, Li, et al (2019) [256] | High | Low | Low | High | **High** |
| Zhang et al (2020) [257] | Low | Low | Low | High | **High** |
| Ziermans et al (2014) [258] | Low | Low | Low | High | **High** |
| Zuithoff et al (2009) [259] | Low | Low | Low | High | **High** |
| **MODEL VALIDATION ANALYSES** | | | | | |
| Addington et al (2010) [42] {1} | Low | Low | Low | High | **High** |
| Addington et al (2010) [42] {2} | Low | Low | Low | High | **High** |
| Addington et al (2010) [42] {3} | Low | Low | Low | High | **High** |
| Bares et al (2017) [50] | Unclear | Unclear | Unclear | High | **High** |
| Bellón et al (2011) [54] | Low | Low | Low | High | **High** |
| Birmaher et al (2018) [55] | Low | Low | Low | High | **High** |
| Boscarino et al (2011) [63] | Low | Unclear | Unclear | High | **High** |
| Brathwaite et al (2020) [260]  (note: validation of Rocha et al, 2021 [211]) | High | High | Low | High | **High** |
| Browning et al (2019) [65] | Low | Low | Low | High | **High** |
| Carrión et al (2016) [261]  (note: validation of Cannon et al, 2008 [68]) | Low | Low | Low | High | **High** |
| Cattelani et al (2018) [71]  (note: validation of Okamato & Harasawa, 2011 [199]) | Low | Low | Low | Unclear | **Unclear** |
| Caye et al (2020) [72] | Low | Low | Low | Low | **Low** |
| Cepeda et al (2018) [74] | Low | Low | Low | Unclear | **Unclear** |
| Chekroud et al (2016) [76] | Low | Low | Low | High | **High** |
| Chekroud et al (2018) [77] | Low | High | High | Unclear | **High** |
| Corcoran et al (2018) [87] | Low | Unclear | Low | High | **High** |
| Fazel et al (2017) [100] | Low | Low | Low | Low | **Low** |
| Fazel et al (2019) [101] | Low | Low | Low | Unclear | **Unclear** |
| Furukawa et al (2019) [105] {1} | Low | Low | Low | Low | **Low** |
| Furukawa et al (2019) [105] {2} | Low | Low | Low | Low | **Low** |
| Fusar-Poli et al (2016) [106] | Low | Low | Low | High | **High** |
| Fusar-Poli et al (2017) [107] | Low | Low | Low | Unclear | **Unclear** |
| Fusar-Poli, Davies, et al (2019) [108] | Low | Low | Low | Unclear | **Unclear** |
| Fusar-Poli, Werbeloff, et al (2019) [262]  (note: validation of Fusar-Poli et al, 2017 [107]) | Low | Low | Low | Unclear | **Unclear** |
| Haidl et al (2018) [118]  (note: validation of Ruhrmann et al, 2010 [216]) | Low | Low | Low | High | **High** |
| Hughes et al (2020) [134] | Low | Low | Unclear | High | **High** |
| Kautzky et al (2019) [146] | Low | Low | Low | High | **High** |
| King et al (2008) [153] | Low | Low | Low | High | **High** |
| King, Bottomley, et al (2011) [154] {1} | Low | Low | Low | High | **High** |
| **Study** | **Domain** | | | | |
|  | Participants | Predictors | Outcome | Analysis | **Overall** |
| King, Marston, et al (2011) [155]) | Low | Low | Low | High | **High** |
| King et al (2013) [263]  (note: validation of King et al, 2008 [153]) | Low | Low | Low | High | **High** |
| Klein et al (2018) [157] | Low | Low | Low | Unclear | **Unclear** |
| Koutsouleris et al (2016) [160] {1} | Low | Low | Low | High | **High** |
| Leighton, Krishnadas, et al (2019) [164] {1} | Low | Low | Low | High | **High** |
| Leighton, Krishnadas, et al (2019) [164] {2} | Low | Low | Low | High | **High** |
| Leighton, Krishnadas, et al (2019) [164] {3} | Low | Low | Low | High | **High** |
| Leighton, Upthegrove, et al (2019) [165] {1} | Low | Low | Low | High | **High** |
| Leighton, Upthegrove, et al (2019) [165] {2} | Low | Low | Low | High | **High** |
| Leighton, Upthegrove, et al (2019) [165] {3} | Low | Low | Low | High | **High** |
| Leighton, Upthegrove, et al (2019) [165] {4} | Low | Low | Low | High | **High** |
| Liu et al (2015) [176] | Low | Low | Low | Unclear | **Unclear** |
| Maarsingh et al (2018) [181] | Low | Low | Low | High | **High** |
| Morrow et al (2020) [187] | Low | Low | Low | Unclear | **Unclear** |
| Negatsch et al (2019) [264]  (note: validation of Fazel et al, 2017 [100]) | Low | High | Unclear | High | **High** |
| Nie et al (2018) [193] | Low | Low | Low | High | **High** |
| Nigatu, Liu, and Wang (2016) [265]  (note: validation of King et al, 2008 [153]) | Low | Low | Low | High | **High** |
| Nigatu and Wang (2019) [266]  (note: validation of King et al, 2011 [154] {1}) | Low | Low | Low | High | **High** |
| Oh et al (2019) [198] {1} | Low | Unclear | Low | High | **High** |
| Osborne and Mittal (2019) [267]  (note: validation of Cannon et al, 2008 [68]) | High | Low | Low | High | **High** |
| Osborne and Mittal (2019) [267]  (note: validation of Zhang, Xu, Tiang et al, 2019 [255]) | High | Low | Low | High | **High** |
| Pradier et al (2021) [205] | Low | Low | Low | Unclear | **Unclear** |
| Raket et al (2020) [207] | Low | Low | Low | Unclear | **Unclear** |
| Reps et al (2020) [208] | Low | Low | Low | High | **High** |
| Rezaii et al (2019) [209] | Low | High | Low | High | **High** |
| Rocha et al (2021) [211] | Low | Low | Low | Low | **Low** |
| Russo et al (2013) [217] | Low | Low | Low | High | **High** |
| Sau and Bhakta (2017b) [219] | Low | Unclear | Unclear | High | **High** |
| Schultebraucks, Shalev, et al (2020) [223] {1} | Low | Low | Low | High | **High** |
| Schultebraucks, Shalev, et al (2020) [223] {2} | Low | Low | Low | High | **High** |
| Thompson et al (2011) [232]  (note: validation of Cannon et al, 2008 [68]) | Low | Low | Low | High | **High** |
| van der Aa et al (2016) [237] | Low | Low | Low | High | **High** |
| van Loo et al (2015) [239] | Low | Low | Low | High | **High** |
| van Loo et al (2018) [240] {2} | Low | Low | Low | Unclear | **Unclear** |
| van Loo et al (2018) [240] {3} | Low | Low | Low | Unclear | **Unclear** |
| Wang et al (2013) [244] {1} | Low | Low | Low | Unclear | **Unclear** |
| Wang et al (2013) [244] {2} | Low | Low | Low | Unclear | **Unclear** |
| Wang, Patten, et al (2014) [245] | Low | Low | Low | Unclear | **Unclear** |
| Wang, Sareen, et al (2014) [246] | Low | Low | Low | Unclear | **Unclear** |
| Zhang, Xu, Tang, et al (2019) [255] | High | Low | Low | High | **High** |
| Zhang, Xu, Li, et al (2019) [256] | High | Low | Low | High | **High** |

Low = low risk of bias; Unclear = unclear risk of bias; High = high risk of bias.

PROBAST items presented in **Appendix 4**. In brief, a domain was only considered ‘low risk’ when all constituent items were rated accordingly, while the presence of at least one item indicating bias risk resulted in a high risk of bias rating for the entire domain. Similarly, a low risk of bias rating for the study as a whole was only ascribed where all four domain (participants, predictors, outcomes, analysis) were rated low risk, with a high rating on any one domain dictating a high overall risk of bias rating. Full item descriptions and rating guidelines are available elsewhere [36,37].

**Supplementary References**

1. Cook IA. Biomarkers in psychiatry: potentials, pitfalls, and pragmatics. Prim Psychiatry*.* 2008;15:54-59. <https://doi.org/10.1186/1755-7682-3-1>.

2. Arbabshirani MR, Plis S, Sui J, Calhoun VD. Single subject prediction of brain disorders in neuroimaging: promises and pitfalls. NeuroImage*.* 2017;145:137-165. <https://doi.org/10.1016/j.neuroimage.2016.02.079>.

3. Schnack HG, Kahn RS. Detecting neuroimaging biomarkers for psychiatric disorders: sample size matters. Front Psychiatry*.* 2016;7:50. <https://doi.org/10.3389/fpsyt.2016.00050>.

4. Flint C, Cearns M, Opel N, Redlich R, Mehler DMA, Emden D, et al. Systematic overestimation of machine learning performance in neuroimaging studies of depression. In. arXiv2019:arXiv:1912.06686.

5. Botvinik-Nezer R, Holzmeister F, Camerer CF, Dreber A, Huber J, Johannesson M, et al. Variability in the analysis of a single neuroimaging dataset by many teams. Nature*.* 2020;582:84-88. <https://doi.org/10.1038/s41586-020-2314-9>.

6. Rashid B, Calhoun V. Towards a brain-based predictome of mental illness. Hum Brain Mapp*.* 2020;41:3468-3535. <https://doi.org/10.1002/hbm.25013>.

7. Bracher-Smith M, Crawford K, Escott-Price V. Machine learning for genetic prediction of psychiatric disorders: a systematic review. Mol Psychiatry*.* 2021;26:70-79. <https://doi.org/10.1038/s41380-020-0825-2>.

8. Cohen ZD, DeRubeis RJ. Treatment selection in depression. Annu Rev Clin Psychol*.* 2018;14:209-236. <https://doi.org/10.1146/annurev-clinpsy-050817-084746>.

9. DeRubeis RJ, Cohen ZD, Forand NR, Fournier JC, Gelfand LA, Lorenzo-Luaces L. The personalized advantage index: translating research on prediction into individualized treatment recommendations. a demonstration. PLoS One*.* 2014;9:e83875. <https://doi.org/10.1371/journal.pone.0083875>.

10. Fusar-Poli P, Hijazi Z, Stahl D, Steyerberg EW. The science of prognosis in psychiatry: a review. JAMA Psychiatry*.* 2018;75:1289-1297. <https://doi.org/10.1001/jamapsychiatry.2018.2530>.

11. Ogundimu EO, Altman DG, Collins GS. Adequate sample size for developing prediction models is not simply related to events per variable. J Clin Epidemiol*.* 2016;76:175-182. <https://doi.org/10.1016/j.jclinepi.2016.02.031>.

12. Steyerberg EW. Clinical prediction models: a practical approach to development, validation, and updating*.* 2nd ed. New York: Springer; 2019.

13. Fusar-Poli P, Stringer D, M. S. Durieux A, Rutigliano G, Bonoldi I, De Micheli A, et al. Clinical-learning versus machine-learning for transdiagnostic prediction of psychosis onset in individuals at-risk. Transl Psychiatry*.* 2019;9:259. <https://doi.org/10.1038/s41398-019-0600-9>.

14. Riley RD, Snell KIE, Martin GP, Whittle R, Archer L, Sperrin M, et al. Penalization and shrinkage methods produced unreliable clinical prediction models especially when sample size was small. J Clin Epidemiol*.* 2021;132:88-96. <https://doi.org/10.1016/j.jclinepi.2020.12.005>.

15. Austin PC, Steyerberg EW. Events per variable (EPV) and the relative performance of different strategies for estimating the out-of-sample validity of logistic regression models. Stat Methods Med Res*.* 2017;26:796-808. <https://doi.org/10.1177/0962280214558972>.

16. Collins GS, Ogundimu EO, Altman DG. Sample size considerations for the external validation of a multivariable prognostic model: a resampling study. Stat Med*.* 2016;35:214-226. <https://doi.org/10.1002/sim.6787>.

17. Debray TPA, Vergouwe Y, Koffijberg H, Nieboer D, Steyerberg EW, Moons KGM. A new framework to enhance the interpretation of external validation studies of clinical prediction models. J Clin Epidemiol*.* 2015;68:279-289. <https://doi.org/10.1016/j.jclinepi.2014.06.018>.

18. Steyerberg EW, Harrell FE, Jr. Prediction models need appropriate internal, internal-external, and external validation. J Clin Epidemiol*.* 2016;69:245-247. <https://doi.org/10.1016/j.jclinepi.2015.04.005>.

19. Kappen TH, van Klei WA, van Wolfswinkel L, Kalkman CJ, Vergouwe Y, Moons KGM. Evaluating the impact of prediction models: lessons learned, challenges, and recommendations. Diagn Progn Res*.* 2018;2:11. <https://doi.org/10.1186/s41512-018-0033-6>.

20. Moons KGM, de Groot JA, Bouwmeester W, Vergouwe Y, Mallett S, Altman DG, et al. Critical appraisal and data extraction for systematic reviews of prediction modelling studies: the CHARMS checklist. PLoS Med*.* 2014;11:e1001744. <https://doi.org/10.1371/journal.pmed.1001744>.

21. Collins GS, Reitsma JB, Altman DG, Moons KG. Transparent reporting of a multivariable prediction model for individual prognosis or diagnosis (TRIPOD): the TRIPOD statement. BMJ*.* 2015;350:g7594. <https://doi.org/10.1136/bmj.g7594>.

22. Adolfsson J, Steineck G. Prognostic and treatment-predictive factors – is there a difference? Prostate Cancer Prostatic Dis*.* 2000;3:265-268. <https://doi.org/10.1038/sj.pcan.4500490>.

23. Clark GM. Prognostic factors versus predictive factors: examples from a clinical trial of erlotinib. Mol Oncol*.* 2008;1:406-412. <https://doi.org/10.1016/j.molonc.2007.12.001>.

24. Ballman KV. Biomarker: predictive or prognostic? Journal of Clinical Oncology*.* 2015;33:3968-3971. <https://doi.org/10.1200/jco.2015.63.3651>.

25. Riley RD, Snell KI, Ensor J, Burke DL, Harrell Jr FE, Moons KG, et al. Minimum sample size for developing a multivariable prediction model: PART II - binary and time-to-event outcomes. Stat Med*.* 2019;38:1276-1296. <https://doi.org/10.1002/sim.7992>.

26. Salazar de Pablo G, Studerus E, Vaquerizo-Serrano J, Irving J, Catalan A, Oliver D, et al. Implementing precision psychiatry: a systematic review of individualized prediction models for clinical practice. Schizophr Bull*.* 2021;47:284-297. <https://doi.org/10.1093/schbul/sbaa120>.

27. Kassraian-Fard P, Matthis C, Balsters JH, Maathuis MH, Wenderoth N. Promises, pitfalls, and basic guidelines for applying machine learning classifiers to psychiatric imaging data, with autism as an example. Front Psychiatry*.* 2016;7:177. <https://doi.org/10.3389/fpsyt.2016.00177>.

28. Ambroise C, McLachlan GJ. Selection bias in gene extraction on the basis of microarray gene-expression data. Proc Natl Acad Sci USA*.* 2002;99:6562-6566.

29. Dupuy A, Simon RM. Critical review of published microarray studies for cancer outcome and guidelines on statistical analysis and reporting. J Natl Cancer Inst*.* 2007;99:147-157. <https://doi.org/10.1093/jnci/djk018>.

30. Ball TM, Squeglia LM, Tapert SF, Paulus MP. Double dipping in machine learning: problems and solutions. Biol Psychiatry Cogn Neurosci Neuroimaging*.* 2020;5:261-263. <https://doi.org/10.1016/j.bpsc.2019.09.003>.

31. Poldrack RA, Huckins G, Varoquaux G. Establishment of best practices for evidence for prediction: a review. JAMA Psychiatry*.* 2020;77:534-540. <https://doi.org/10.1001/jamapsychiatry.2019.3671>.

32. Harrell Jr FE. Regression modeling strategies: with applications to linear models, logistic and ordinal regression, and survival analysis*.* 2nd ed. New York, NY: Springer; 2015.

33. Harrell Jr FE, Califf RM, Pryor DB, Lee KL, Rosati RA. Evaluating the yield of medical tests. JAMA*.* 1982;247:2543-2546.

34. Steyerberg EW, Vickers AJ, Cook NR, Gerds T, Gonen M, Obuchowski N, et al. Assessing the performance of prediction models: a framework for traditional and novel measures. Epidemiology*.* 2010;21:128-138. <https://doi.org/10.1097/EDE.0b013e3181c30fb2>.

35. Hosmer DW, Lemeshow S, Sturdivant RX. Applied logistic regression*.* 3rd ed. New York, NY: John Wiley & Sons; 2013.

36. Moons KGM, Wolff RF, Riley RD, Whiting PF, Westwood M, Collins GS, et al. PROBAST: a tool to assess risk of bias and applicability of prediction model studies: explanation and elaboration. Ann Intern Med*.* 2019;170:W1-W33. <https://doi.org/10.7326/M18-1377>.

37. Wolff RF, Moons KGM, Riley RD, Whiting PF, Westwood M, Collins GS, et al. PROBAST: a tool to assess the risk of bias and applicability of prediction model studies. Ann Intern Med*.* 2019;170:51-58. <https://doi.org/10.7326/M18-1376>.

38. Cohen J. A coefficient of agreement for nominal scales. Educ Psychol Meas*.* 1960;20:37-46.

39. Page MJ, McKenzie JE, Bossuyt PM, Boutron I, Hoffmann TC, Mulrow CD, et al. The PRISMA 2020 statement: an updated guideline for reporting systematic reviews. BMJ*.* 2021;372:n71. <https://doi.org/10.1136/bmj.n71>.

40. Abdullah-Koolmees H, Gardarsdottir H, Minnema LA, Elmi K, Stoker LJ, Vuyk J, et al. Predicting rehospitalization in patients treated with antipsychotics: a prospective observational study. Ther Adv Psychopharmacol*.* 2018;8:213-229. <https://doi.org/10.1177/2045125318762373>.

41. Acion L, Kelmansky D, Laan MDV, Sahker E, Jones D, Arndt S. Use of a machine learning framework to predict substance use disorder treatment success. PLoS ONE*.* 2017;12:e0175383. <https://doi.org/10.1371/journal.pone.0175383>.

42. Addington DE, Beck C, Wang J, Adams B, Pryce C, Zhu H, et al. Predictors of admission in first-episode psychosis: developing a risk adjustment model for service comparisons. Psychiatr Serv*.* 2010;61:483-488. <https://doi.org/10.1176/ps.2010.61.5.483>.

43. Addington J, Liu L, Perkins DO, Carrion RE, Keefe RS, Woods SW. The role of cognition and social functioning as predictors in the transition to psychosis for youth with attenuated psychotic symptoms. Schizophr Bull*.* 2017;43:57-63. <https://doi.org/10.1093/schbul/sbw152>.

44. Agne NA, Tisott CG, Ballester P, Passos IC, Ferrão YA. Predictors of suicide attempt in patients with obsessive-compulsive disorder: an exploratory study with machine learning analysis. Psychol Med*.* 2020. <https://doi.org/10.1017/s0033291720002329>.

45. Ahn WY, Ramesh D, Moeller FG, Vassileva J. Utility of machine-learning approaches to identify behavioral markers for substance use disorders: impulsivity dimensions as predictors of current cocaine dependence. Front Psychiatry*.* 2016;7:34. <https://doi.org/10.3389/fpsyt.2016.00034>.

46. Ahn WY, Vassileva J. Machine-learning identifies substance-specific behavioral markers for opiate and stimulant dependence. Drug Alcohol Depend*.* 2016;161:247-257. <https://doi.org/10.1016/j.drugalcdep.2016.02.008>.

47. Amminger GP, Mechelli A, Rice S, Kim SW, Klier CM, McNamara RK, et al. Predictors of treatment response in young people at ultra-high risk for psychosis who received long-chain omega-3 fatty acids. Transl Psychiatry*.* 2015;5:e495. <https://doi.org/10.1038/tp.2014.134>.

48. Andrews JA, Harrison RF, Brown LJE, MacLean LM, Hwang F, Smith T, et al. Using the NANA toolkit at home to predict older adults' future depression. J Affect Disord*.* 2017;213:187-190. <https://doi.org/10.1016/j.jad.2017.02.019>.

49. Askland KD, Garnaat S, Sibrava NJ, Boisseau CL, Strong D, Mancebo M, et al. Prediction of remission in obsessive compulsive disorder using a novel machine learning strategy. Int J Methods Psychiatr*.* 2015;24:156-169. <https://doi.org/10.1002/mpr.1463>.

50. Bares M, Novak T, Brunovsky M, Kopecek M, Hoschl C. The comparison of effectiveness of various potential predictors of response to treatment with SSRIs in patients with depressive disorder. J Nerv Ment Dis*.* 2017;205:618-626. <https://doi.org/10.1097/NMD.0000000000000574>.

51. Barnes SM, Monteith LL, Forster JE, Nazem S, Borges LM, Stearns-Yoder KA, et al. Developing predictive models to enhance clinician prediction of suicide attempts among veterans with and without PTSD. Suicide Life Threat Behav*.* 2019;49:1094-1104. <https://doi.org/10.1111/sltb.12511>.

52. Barros J, Morales S, Echavarri O, Garcia A, Ortega J, Asahi T, et al. Suicide detection in Chile: proposing a predictive model for suicide risk in a clinical sample of patients with mood disorders. Braz J Psychiatry*.* 2017;39:1-11. <https://doi.org/10.1590/1516-4446-2015-1877>.

53. Bauer IE, Suchting R, Van Rheenen TE, Wu M-J, Mwangi B, Spiker D, et al. The use of component-wise gradient boosting to assess the possible role of cognitive measures as markers of vulnerability to pediatric bipolar disorder. Cogn Neuropsychol*.* 2019;24:93-107. <https://doi.org/10.1080/13546805.2019.1580190>.

54. Bellón JA, de Dios Luna J, King M, Moreno-Kustner B, Nazareth I, Monton-Franco C, et al. Predicting the onset of major depression in primary care: international validation of a risk prediction algorithm from Spain. Psychol Med*.* 2011;41:2075-2088. <https://doi.org/10.1017/s0033291711000468>.

55. Birmaher B, Merranko JA, Goldstein TR, Gill MK, Goldstein BI, Hower H, et al. A risk calculator to predict the individual risk of conversion from subthreshold bipolar symptoms to bipolar disorder I or II in youth. J Am Acad Child Adolesc Psychiatry*.* 2018;57:755-763. <https://doi.org/10.1016/j.jaac.2018.05.023>.

56. Birmaher B, Merranko JA, Gill MK, Hafeman D, Goldstein T, Goldstein B, et al. Predicting personalized risk of mood recurrences in youths and young adults with bipolar spectrum disorder. J Am Acad Child Adolesc Psychiatry*.* 2020;59:1156-1164. <https://doi.org/10.1016/j.jaac.2019.12.005>.

57. Birnbaum ML, Ernala SK, Rizvi AF, Arenare E, A RVM, De Choudhury M, et al. Detecting relapse in youth with psychotic disorders utilizing patient-generated and patient-contributed digital data from Facebook. NPJ Schizophr*.* 2019;5:17. <https://doi.org/10.1038/s41537-019-0085-9>.

58. Birnbaum ML, Kulkarni PP, Van Meter A, Chen V, Rizvi AF, Arenare E, et al. Utilizing machine learning on internet search activity to support the diagnostic process and relapse detection in young individuals with early psychosis: feasibility study. JMIR Ment Health*.* 2020;7:e19348. <https://doi.org/10.2196/19348>.

59. Bishop-Fitzpatrick L, Movaghar A, Greenberg JS, Page D, DaWalt LS, Brilliant MH, et al. Using machine learning to identify patterns of lifetime health problems in decedents with autism spectrum disorder. Autism Res*.* 2018;11:1120-1128. <https://doi.org/10.1002/aur.1960>.

60. Bledsoe JC, Xiao D, Chaovalitwongse A, Mehta S, Grabowski TJ, Semrud-Clikeman M, et al. Diagnostic classification of ADHD versus control: support vector machine classification using brief neuropsychological assessment. J Atten Disord*.* 2016;24:1547-1556. <https://doi.org/10.1177/1087054716649666>.

61. Boer S, Dekkers OM, Cessie SL, Carlier IV, van Hemert AM. Prediction of prolonged treatment course for depressive and anxiety disorders in an outpatient setting: the Leiden routine outcome monitoring study. J Affect Disord*.* 2019;247:81-87. <https://doi.org/10.1016/j.jad.2018.12.035>.

62. Bokma WA, Zhutovsky P, Giltay EJ, Schoevers RA, Penninx B, van Balkom A, et al. Predicting the naturalistic course in anxiety disorders using clinical and biological markers: a machine learning approach. Psychol Med*.* 2020. <https://doi.org/10.1017/s0033291720001658>.

63. Boscarino JA, Kirchner HL, Hoffman SN, Sartorius J, Adams RE, Figley CR. A brief screening tool for assessing psychological trauma in clinical practice: development and validation of the New York PTSD Risk Score. Gen Hosp Psychiatry*.* 2011;33:489-500. <https://doi.org/10.1016/j.genhosppsych.2011.06.001>.

64. Breen MS, Thomas KGF, Baldwin DS, Lipinska G. Modelling PTSD diagnosis using sleep, memory, and adrenergic metabolites: an exploratory machine-learning study. Hum Psychopharmacol*.* 2019;34:e2691. <https://doi.org/10.1002/hup.2691>.

65. Browning M, Kingslake J, Dourish CT, Goodwin GM, Harmer CJ, Dawson GR. Predicting treatment response to antidepressant medication using early changes in emotional processing. Eur Neuropsychopharmacol*.* 2019;29:66-75. <https://doi.org/10.1016/j.euroneuro.2018.11.1102>.

66. Bussu G, Jones EJH, Charman T, Johnson MH, Buitelaar JK, Team B. Prediction of autism at 3 years from behavioural and developmental measures in high-risk infants: a longitudinal cross-domain classifier analysis. J Autism Dev Disord*.* 2018;48:2418-2433. <https://doi.org/10.1007/s10803-018-3509-x>.

67. Cannon TD, Cadenhead K, Cornblatt B, Woods SW, Addington J, Walker E, et al. Prediction of psychosis in youth at high clinical risk: a multisite longitudinal study in North America. Arch Gen Psychiatry*.* 2008;65:28-37. <https://doi.org/10.1001/archgenpsychiatry.2007.3>.

68. Cannon TD, Yu C, Addington J, Bearden CE, Cadenhead KS, Cornblatt BA, et al. An individualized risk calculator for research in prodromal psychosis. Am J Psychiatry*.* 2016;173:980-988. <https://doi.org/10.1176/appi.ajp.2016.15070890>.

69. Carrión RE, McLaughlin D, Goldberg TE, Auther AM, Olsen RH, Olvet DM, et al. Prediction of functional outcome in individuals at clinical high risk for psychosis. JAMA Psychiatry*.* 2013;70:1133-1142. <https://doi.org/10.1001/jamapsychiatry.2013.1909>.

70. Carter JW, Schulsinger F, Parnas J, Cannon T, Mednick SA. A multivariate prediction model of schizophrenia. Schizophr Bull*.* 2002;28:649-682. <https://doi.org/10.1093/oxfordjournals.schbul.a006971>.

71. Cattelani L, Murri MB, Chesani F, Chiari L, Bandinelli S, Palumbo P. Risk prediction model for late life depression: development and validation on three large european datasets. IEEE J Biomed Health Inform*.* 2019;23:2196-2204. <https://doi.org/10.1109/jbhi.2018.2884079>.

72. Caye A, Agnew-Blais J, Arseneault L, Gonçalves H, Kieling C, Langley K, et al. A risk calculator to predict adult attention-deficit/hyperactivity disorder: generation and external validation in three birth cohorts and one clinical sample. Epidemiol Psychiatr Sci*.* 2020;29:e37. <https://doi.org/10.1017/S2045796019000283>.

73. Cearns M, Opel N, Clark S, Kaehler C, Thalamuthu A, Heindel W, et al. Predicting rehospitalization within 2 years of initial patient admission for a major depressive episode: a multimodal machine learning approach. Transl Psychiatry*.* 2019;9:285. <https://doi.org/10.1038/s41398-019-0615-2>.

74. Cepeda MS, Reps J, Fife D, Blacketer C, Stang P, Ryan P. Finding treatment-resistant depression in real-world data: how a data-driven approach compares with expert-based heuristics. Depress Anxiety*.* 2018;35:220-228. <https://doi.org/10.1002/da.22705>.

75. Chang YC, Lane HY, Yang KH, Huang CL. Optimizing early prediction for antipsychotic response in schizophrenia. J Clin Psychopharmacol*.* 2006;26:554-559. <https://doi.org/10.1097/01.jcp.0000246211.95905.8c>.

76. Chekroud AM, Zotti RJ, Shehzad Z, Gueorguieva R, Johnson MK, Trivedi MH, et al. Cross-trial prediction of treatment outcome in depression: a machine learning approach. Lancet Psychiatry*.* 2016;3:243-250. <https://doi.org/10.1016/s2215-0366(15)00471-x>.

77. Chekroud AM, Foster D, Zheutlin AB, Gerhard DM, Roy B, Koutsouleris N, et al. Predicting barriers to treatment for depression in a U.S. national sample: a cross-sectional, proof-of-concept study. Psychiatr Serv*.* 2018;69:927-934. <https://doi.org/10.1176/appi.ps.201800094>.

78. Cho CH, Lee T, Kim MG, In HP, Kim L, Lee HJ. Mood prediction of patients with mood disorders by machine learning using passive digital phenotypes based on the circadian rhythm: prospective observational cohort study. J Med Internet Res*.* 2019;21:e11029. <https://doi.org/10.2196/11029>.

79. Choi J, Choi J, Jung HT. Applying machine-learning techniques to build self-reported depression prediction models. Comput Inform Nurs*.* 2018;36:317-321. <https://doi.org/10.1097/cin.0000000000000463>.

80. Chondros P, Davidson S, Wolfe R, Gilchrist G, Dowrick C, Griffiths F, et al. Development of a prognostic model for predicting depression severity in adult primary patients with depressive symptoms using the diamond longitudinal study. J Affect Disord*.* 2018;227:854-860. <https://doi.org/10.1016/j.jad.2017.11.042>.

81. Christensen MC, Mayer SA, Ferran JM, Kissela B. Depressed mood after intracerebral hemorrhage: the FAST trial. Cerebrovasc Dis*.* 2009;27:353-360. <https://doi.org/10.1159/000202012>.

82. Chua YC, Abdin E, Tang C, Subramaniam M, Verma S. First-episode psychosis and vocational outcomes: A predictive model. Schizophr Res*.* 2019;211:63-68. <https://doi.org/10.1016/j.schres.2019.07.009>.

83. Chung Y, Addington J, Bearden CE, Cadenhead K, Cornblatt B, Mathalon DH, et al. Adding a neuroanatomical biomarker to an individualized risk calculator for psychosis: A proof-of-concept study. Schizophr Res*.* 2019;208:41-43. <https://doi.org/10.1016/j.schres.2019.01.026>.

84. Ciarleglio AJ, Brucato G, Masucci MD, Altschuler R, Colibazzi T, Corcoran CM, et al. A predictive model for conversion to psychosis in clinical high-risk patients. Psychol Med*.* 2019;49:1128-1137. <https://doi.org/10.1017/s003329171800171x>.

85. Clark SR, Baune BT, Schubert KO, Lavoie S, Smesny S, Rice SM, et al. Prediction of transition from ultra-high risk to first-episode psychosis using a probabilistic model combining history, clinical assessment and fatty-acid biomarkers. Transl Psychiatry*.* 2016;6:e897. <https://doi.org/10.1038/tp.2016.170>.

86. Connor JP, Symons M, Feeney GFX, Young RM, Wiles J. The application of machine learning techniques as an adjunct to clinical decision making in alcohol dependence treatment. Subst Use Misuse*.* 2007;42:2193-2206. <https://doi.org/10.1080/10826080701658125>.

87. Corcoran CM, Carrillo F, Fernandez-Slezak D, Bedi G, Klim C, Javitt DC, et al. Prediction of psychosis across protocols and risk cohorts using automated language analysis. World Psychiatry*.* 2018;17:67-75. <https://doi.org/10.1002/wps.20491>.

88. Cornblatt BA, Carrión RE, Auther A, McLaughlin D, Olsen RH, John M, et al. Psychosis prevention: a modified clinical high risk perspective from the Recognition And Prevention (RAP) program. Am J Psychiatry*.* 2015;172:986-994. <https://doi.org/10.1176/appi.ajp.2015.13121686>.

89. Crippa A, Salvatore C, Molteni E, Mauri M, Salandi A, Trabattoni S, et al. The utility of a computerized algorithm based on a multi-domain profile of measures for the diagnosis of attention deficit/hyperactivity disorder. Front Psychiatry*.* 2017;8:189. <https://doi.org/10.3389/fpsyt.2017.00189>.

90. Davidson M, Reichenberg A, Rabinowitz J, Weiser M, Kaplan Z, Mark M. Behavioral and intellectual markers for schizophrenia in apparently healthy male adolescents. Am J Psychiatry*.* 1999;156:1328-1335. <https://doi.org/10.1176/ajp.156.9.1328>.

91. de Man-van Ginkel JM, Hafsteinsdottir TB, Lindeman E, Ettema RG, Grobbee DE, Schuurmans MJ. In-hospital risk prediction for post-stroke depression: development and validation of the Post-stroke Depression Prediction Scale. Stroke*.* 2013;44:2441-2445. <https://doi.org/10.1161/strokeaha.111.000304>.

92. Delgadillo J, Huey D, Bennett H, McMillan D. Case complexity as a guide for psychological treatment selection. J Consult Clin Psychol*.* 2017;85:835-853. <https://doi.org/10.1037/ccp0000231>.

93. Delgadillo J, Gonzalez Salas Duhne P. Targeted prescription of cognitive-behavioral therapy versus person-centered counseling for depression using a machine learning approach. J Consult Clin Psychol*.* 2020;88:14-24. <https://doi.org/10.1037/ccp0000476>.

94. Demetriou EA, Park SH, Ho N, Pepper KL, Song YJC, Naismith SL, et al. Machine learning for differential diagnosis between clinical conditions with social difficulty: autism spectrum disorder, early psychosis, and social anxiety disorder. Front Psychiatry*.* 2020;11:545. <https://doi.org/10.3389/fpsyt.2020.00545>.

95. Dennis CL, Janssen PA, Singer J. Identifying women at-risk for postpartum depression in the immediate postpartum period. Acta Psychiatr Scand*.* 2004;110:338-346. <https://doi.org/10.1111/j.1600-0447.2004.00337.x>.

96. Denys D, Burger H, van Megen H, de Geus F, Westenberg H. A score for predicting response to pharmacotherapy in obsessive-compulsive disorder. Int Clin Psychopharmacol*.* 2003;18:315-322. <https://doi.org/10.1097/00004850-200311000-00002>.

97. Dinga R, Marquand AF, Veltman DJ, Beekman AT, Schoevers RA, van Hemert AM, et al. Predicting the naturalistic course of depression from a wide range of clinical, psychological, and biological data: a machine learning approach. Transl Psychiatry*.* 2018;8:1-11. <https://doi.org/10.1038/s41398-018-0289-1>.

98. Dipnall JF, Pasco JA, Berk M, Williams LJ, Dodd S, Jacka FN, et al. Getting RID of the blues: formulating a Risk Index for Depression (RID) using structural equation modeling. Aust N Z J Psychiatry*.* 2017;51:1121-1133. <https://doi.org/10.1177/0004867417726860>.

99. Edgcomb J, Shaddox T, Hellemann G, Brooks JO, 3rd. High-risk phenotypes of early psychiatric readmission in bipolar disorder with comorbid medical illness. Psychosomatics*.* 2019;60:563-573. <https://doi.org/10.1016/j.psym.2019.05.002>.

100. Fazel S, Wolf A, Larsson H, Lichtenstein P, Mallett S, Fanshawe TR. Identification of low risk of violent crime in severe mental illness with a clinical prediction tool (Oxford Mental Illness and Violence tool [OxMIV]): a derivation and validation study. Lancet Psychiatry*.* 2017;4:461-468. <https://doi.org/10.1016/S2215-0366(17)30109-8>.

101. Fazel S, Wolf A, Larsson H, Mallett S, Fanshawe TR. The prediction of suicide in severe mental illness: development and validation of a clinical prediction rule (OxMIS). Transl Psychiatry*.* 2019;9:98. <https://doi.org/10.1038/s41398-019-0428-3>.

102. Flygare O, Enander J, Andersson E, Ljótsson B, Ivanov VZ, Mataix-Cols D, et al. Predictors of remission from body dysmorphic disorder after internet-delivered cognitive behavior therapy: a machine learning approach. BMC Psychiatry*.* 2020;20:247. <https://doi.org/10.1186/s12888-020-02655-4>.

103. Fond G, Bulzacka E, Boucekine M, Schurhoff F, Berna F, Godin O, et al. Machine learning for predicting psychotic relapse at 2 years in schizophrenia in the national FACE-SZ cohort. Prog Neuropsychopharmacol Biol Psychiatry*.* 2019;92:8-18. <https://doi.org/10.1016/j.pnpbp.2018.12.005>.

104. Francesconi M, Minichino A, Carrión RE, Delle Chiaie R, Bevilacqua A, Parisi M, et al. Psychosis prediction in secondary mental health services. a broad, comprehensive approach to the “at risk mental state” syndrome. Eur Psychiatry*.* 2017;40:96-104. <https://doi.org/10.1016/j.eurpsy.2016.09.002>.

105. Furukawa TA, Kato T, Shinagawa Y, Miki K, Fujita H, Tsujino N, et al. Prediction of remission in pharmacotherapy of untreated major depression: development and validation of multivariable prediction models. Psychol Med*.* 2019;49:2405-2413. <https://doi.org/10.1017/s0033291718003331>.

106. Fusar-Poli P, Rutigliano G, Stahl D, Schmidt A, Ramella-Cravaro V, Hitesh S, et al. Deconstructing pretest risk enrichment to optimize prediction of psychosis in individuals at clinical high risk. JAMA Psychiatry*.* 2016;73:1260-1267. <https://doi.org/10.1001/jamapsychiatry.2016.2707>.

107. Fusar-Poli P, Rutigliano G, Stahl D, Davies C, Bonoldi I, Reilly T, et al. Development and validation of a clinically based risk calculator for the transdiagnostic prediction of psychosis. JAMA Psychiatry*.* 2017;74:493-500. <https://doi.org/10.1001/jamapsychiatry.2017.0284>.

108. Fusar-Poli P, Davies C, Rutigliano G, Stahl D, Bonoldi I, McGuire P. Transdiagnostic individualized clinically based risk calculator for the detection of individuals at risk and the prediction of psychosis: model refinement including nonlinear effects of age. Front Psychiatry*.* 2019;10:313. <https://doi.org/10.3389/fpsyt.2019.00313>.

109. Galatzer-Levy IR, Karstoft KI, Statnikov A, Shalev AY. Quantitative forecasting of PTSD from early trauma responses: a machine learning application. J Psychiatr Res*.* 2014;59:68-76. <https://doi.org/10.1016/j.jpsychires.2014.08.017>.

110. Galatzer-Levy IR, Ma S, Statnikov A, Yehuda R, Shalev AY. Utilization of machine learning for prediction of post-traumatic stress: a re-examination of cortisol in the prediction and pathways to non-remitting PTSD. Transl Psychiatry*.* 2017;7:e1070. <https://doi.org/10.1038/tp.2017.38>.

111. Gan Z, Diao F, Wei Q, Wu X, Cheng M, Guan N, et al. A predictive model for diagnosing bipolar disorder based on the clinical characteristics of major depressive episodes in Chinese population. J Affect Disord*.* 2011;134:119-125. <https://doi.org/10.1016/j.jad.2011.05.054>.

112. Ge F, Jiang J, Wang Y, Yuan C, Zhang W. Identifying suicidal ideation among Chinese patients with major depressive disorder: evidence from a real-world hospital-based study in China. Neuropsychiatr Dis Treat*.* 2020;16:665-672. <https://doi.org/10.2147/ndt.S238286>.

113. Ge F, Li Y, Yuan M, Zhang J, Zhang W. Identifying predictors of probable posttraumatic stress disorder in children and adolescents with earthquake exposure: a longitudinal study using a machine learning approach. J Affect Disord*.* 2020;264:483-493. <https://doi.org/10.1016/j.jad.2019.11.079>.

114. Gilman SE, Dupuy JM, Perlis RH. Prospective, population-based study of the transition from major depressive disorder to bipolar disorder. J Clin Psychiatry*.* 2012;73:829-836. <https://doi.org/10.4088/JCP.11m06912>.

115. Gonzalez-Gutierrez MV, Guerrero Velazquez J, Morales Garcia C, Casas Maldonado F, Gomez Jimenez FJ, Gonzalez Vargas F. Predictive model for anxiety and depression in Spanish patients with stable chronic cbstructive pulmonary disease. Arch Bronconeumol*.* 2016;52:151-157. <https://doi.org/10.1016/j.arbres.2015.09.003>.

116. Gu S-C, Zhou J, Yuan C-X, Ye Q. Personalized prediction of depression in patients with newly diagnosed Parkinson's disease: a prospective cohort study. J Affect Disord*.* 2020;268:118-126. <https://doi.org/10.1016/j.jad.2020.02.046>.

117. Hafeman DM, Merranko J, Goldstein TR, Axelson D, Goldstein BI, Monk K, et al. Assessment of a person-level risk calculator to predict new-onset bipolar spectrum disorder in youth at familial risk. JAMA Psychiatry*.* 2017;74:841-847. <https://doi.org/10.1001/jamapsychiatry.2017.1763>.

118. Haidl T, Rosen M, Schultze-Lutter F, Nieman D, Eggers S, Heinimaa M, et al. Expressed emotion as a predictor of the first psychotic episode – results of the European prediction of psychosis study. Schizophr Res*.* 2018;199:346-352. <https://doi.org/10.1016/j.schres.2018.03.019>.

119. Han D, Fang Y, Luo H. A predictive model offor attention deficit hyperactivity disorder based on clinical assessment tools. Neuropsychiatr Dis Treat*.* 2020;16:1331-1337. <https://doi.org/10.2147/ndt.S245636>.

120. Han DH, Lee S, Seo DC. Using machine learning to predict opioid misuse among U.S. adolescents. Prev Med*.* 2020;130:105886. <https://doi.org/10.1016/j.ypmed.2019.105886>.

121. Han SYS, Tomasik J, Rustogi N, Lago SG, Barton-Owen G, Eljasz P, et al. Diagnostic prediction model development using data from dried blood spot proteomics and a digital mental health assessment to identify major depressive disorder among individuals presenting with low mood. Brain Behav Immun*.* 2020;90:184-195. <https://doi.org/10.1016/j.bbi.2020.08.011>.

122. Hariman K, Cheng KM, Lam J, Leung SK, Lui SSY. Clinical risk model to predict 28-day unplanned readmission via the accident and emergency department after discharge from acute psychiatric units for patients with psychotic spectrum disorders. BJPsych Open*.* 2020;6:e13. <https://doi.org/10.1192/bjo.2019.97>.

123. Harrington KM, Quaden R, Stein MB, Honerlaw JP, Cissell S, Pietrzak RH, et al. Validation of an electronic medical record–based algorithm for identifying posttraumatic stress disorder in US veterans. J Trauma Stress*.* 2019;32:226-237. <https://doi.org/10.1002/jts.22399>.

124. Hatton CM, Paton LW, McMillan D, Cussens J, Gilbody S, Tiffin PA. Predicting persistent depressive symptoms in older adults: a machine learning approach to personalised mental healthcare. J Affect Disord*.* 2019;246:857-860. <https://doi.org/10.1016/j.jad.2018.12.095>.

125. Haynos AF, Wang SB, Lipson S, Peterson CB, Mitchell JE, Halmi KA, et al. Machine learning enhances prediction of illness course: a longitudinal study in eating disorders. Psychol Med*.* 2020. <https://doi.org/10.1017/s0033291720000227>.

126. Hengartner MP, Heekeren K, Dvorsky D, Walitza S, Rossler W, Theodoridou A. Checking the predictive accuracy of basic symptoms against ultra high-risk criteria and testing of a multivariable prediction model: evidence from a prospective three-year observational study of persons at clinical high-risk for psychosis. Eur Psychiatry*.* 2017;45:27-35. <https://doi.org/10.1016/j.eurpsy.2017.05.026>.

127. Hettige NC, Nguyen TB, Yuan C, Rajakulendran T, Baddour J, Bhagwat N, et al. Classification of suicide attempters in schizophrenia using sociocultural and clinical features: a machine learning approach. Gen Hosp Psychiatry*.* 2017;47:20-28. <https://doi.org/10.1016/j.genhosppsych.2017.03.001>.

128. Higginson I, Priest P. Predictors of family anxiety in the weeks before bereavement. Soc Sci Med*.* 1996;43:1621-1625. <https://doi.org/10.1016/s0277-9536(96)00062-7>.

129. Hilbert K, Kunas SL, Lueken U, Kathmann N, Fydrich T, Fehm L. Predicting cognitive behavioral therapy outcome in the outpatient sector based on clinical routine data: a machine learning approach. Behav Res Ther*.* 2020;124:103530. <https://doi.org/10.1016/j.brat.2019.103530>.

130. Hirschfeld RM, Russell JM, Delgado PL, Fawcett J, Friedman RA, Harrison WM, et al. Predictors of response to acute treatment of chronic and double depression with sertraline or imipramine. J Clin Psychiatry*.* 1998;59:669-675. <https://doi.org/10.4088/jcp.v59n1205>.

131. Hoogendoorn M, Berger T, Schulz A, Stolz T, Szolovits P. Predicting social anxiety treatment outcome based on therapeutic email conversations. IEEE J Biomed Health Inform*.* 2017;21:1449-1459. <https://doi.org/10.1109/jbhi.2016.2601123>.

132. Huang P, Tan H, Liu A, Feng S, Chen M. Prediction of posttraumatic stress disorder among adults in flood district. BMC Public Health*.* 2010;10:207. <https://doi.org/10.1186/1471-2458-10-207>.

133. Huang SH, LePendu P, Iyer SV, Tai-Seale M, Carrell D, Shah NH. Toward personalizing treatment for depression: predicting diagnosis and severity. J Am Med Inform Assoc*.* 2014;21:1069-1075. <https://doi.org/10.1136/amiajnl-2014-002733>.

134. Hughes MC, Pradier MF, Ross AS, McCoy TH, Jr., Perlis RH, Doshi-Velez F. Assessment of a prediction model for antidepressant treatment stability using supervised topic models. JAMA Netw Open*.* 2020;3:e205308. <https://doi.org/10.1001/jamanetworkopen.2020.5308>.

135. Iniesta R, Malki K, Maier W, Rietschel M, Mors O, Hauser J, et al. Combining clinical variables to optimize prediction of antidepressant treatment outcomes. J Psychiatr Res*.* 2016;78:94-102. <https://doi.org/10.1016/j.jpsychires.2016.03.016>.

136. Ising HK, Ruhrmann S, Burger NA, Rietdijk J, Dragt S, Klaassen RM, et al. Development of a stage-dependent prognostic model to predict psychosis in ultra-high-risk patients seeking treatment for co-morbid psychiatric disorders. Psychol Med*.* 2016;46:1839-1851. <https://doi.org/10.1017/s0033291716000325>.

137. Jimenez-Serrano S, Tortajada S, Garcia-Gomez JM. A mobile health application to predict postpartum depression based on machine learning. Telemed J E Health*.* 2015;21:567-574. <https://doi.org/10.1089/tmj.2014.0113>.

138. Jin H, Wu S, Di Capua P. Development of a clinical forecasting model to predict comorbid depression among diabetes patients and an application in depression screening policy making. Prev Chronic Dis*.* 2015;12:150047. <https://doi.org/10.5888/pcd12.150047>.

139. Jin H, Wu S, Vidyanti I, Di Capua P, Wu B. Predicting depression among patients with diabetes using longitudinal data. a multilevel regression model. Methods Inf Med*.* 2015;54:553-559. <https://doi.org/10.3414/me14-02-0009>.

140. Jing Y, Hu Z, Fan P, Xue Y, Wang L, Tarter RE, et al. Analysis of substance use and its outcomes by machine learning I. childhood evaluation of liability to substance use disorder. Drug Alcohol Depend*.* 2020;206:107605. <https://doi.org/10.1016/j.drugalcdep.2019.107605>.

141. Kajiwara H, Ohira Y, Ikegami A, Hanazawa N, Masuyama T, Yamashita T, et al. Utilizing a medical questionnaire to predict anxiety and depression in outpatients. Int J Gen Med*.* 2016;9:199-204. <https://doi.org/10.2147/ijgm.S101556>.

142. Karstoft KI, Galatzer-Levy IR, Statnikov A, Li Z, Shalev AY. Bridging a translational gap: using machine learning to improve the prediction of PTSD. BMC Psychiatry*.* 2015;15:30. <https://doi.org/10.1186/s12888-015-0399-8>.

143. Karstoft KI, Statnikov A, Andersen SB, Madsen T, Galatzer-Levy IR. Early identification of posttraumatic stress following military deployment: application of machine learning methods to a prospective study of Danish soldiers. J Affect Disord*.* 2015;184:170-175. <https://doi.org/10.1016/j.jad.2015.05.057>.

144. Kautzky A, Baldinger-Melich P, Kranz GS, Vanicek T, Souery D, Montgomery S, et al. A new prediction model for evaluating treatment-resistant depression. J Clin Psychiatry*.* 2017;78:215-222. <https://doi.org/10.4088/JCP.15m10381>.

145. Kautzky A, Dold M, Bartova L, Spies M, Vanicek T, Souery D, et al. Refining prediction in treatment-resistant depression: results of machine learning analyses in the TRD III sample. J Clin Psychiatry*.* 2018;79:16m11385. <https://doi.org/10.4088/JCP.16m11385>.

146. Kautzky A, Dold M, Bartova L, Spies M, Kranz GS, Souery D, et al. Clinical factors predicting treatment resistant depression: affirmative results from the European multicenter study. Acta Psychiatrica Scandinavica*.* 2019;139:78-88. <https://doi.org/10.1111/acps.12959>.

147. Kendler KS, Ohlsson H, Sundquist J, Sundquist K. Prediction of onset of substance-induced psychotic disorder and its progression to schizophrenia in a Swedish national sample. Am J Psychiatry*.* 2019;176:711-719. <https://doi.org/10.1176/appi.ajp.2019.18101217>.

148. Kessler RC, Rose S, Koenen KC, Karam EG, Stang PE, Stein DJ, et al. How well can post-traumatic stress disorder be predicted from pre-trauma risk factors? sn exploratory study in the WHO World Mental Health Surveys. World Psychiatry*.* 2014;13:265-274. <https://doi.org/10.1002/wps.20150>.

149. Kessler RC, Warner LCH, Ivany LC, Petukhova MV, Rose S, Bromet EJ, et al. Predicting US Army suicides after hospitalizations with psychiatric diagnoses in the Army Study to Assess Risk and Resilience in Servicemembers (Army STARRS). JAMA Psychiatry*.* 2015;72:49-57.

150. Kessler RC, van Loo HM, Wardenaar KJ, Bossarte RM, Brenner LA, Cai T, et al. Testing a machine-learning algorithm to predict the persistence and severity of major depressive disorder from baseline self-reports. Mol Psychiatry*.* 2016;21:1366-1371. <https://doi.org/10.1038/mp.2015.198>.

151. Kim JW, Sharma V, Ryan ND. Predicting methylphenidate response in ADHD using machine learning approaches. Int J Neuropsychopharmacol*.* 2015;18:pyv052. <https://doi.org/10.1093/ijnp/pyv052>.

152. Kim H, Lee S, Lee S, Hong S, Kang H, Kim N. Depression prediction by using ecological momentary assessment, actiwatch data, and machine learning: observational study on older adults living alone. JMIR Mhealth Uhealth*.* 2019;7:e14149. <https://doi.org/10.2196/14149>.

153. King M, Walker C, Levy G, Bottomley C, Royston P, Weich S, et al. Development and validation of an international risk prediction algorithm for episodes of major depression in general practice attendees. Arch Gen Psychiatry*.* 2008;65:1368-1376. <https://doi.org/10.1001/archpsyc.65.12.1368>.

154. King M, Bottomley C, Bellon-Saameno JA, Torres-Gonzalez F, Svab I, Rifel J, et al. An international risk prediction algorithm for the onset of generalized anxiety and panic syndromes in general practice attendees: predictA. Psychol Med*.* 2011;41:1625-1639. <https://doi.org/10.1017/s0033291710002400>.

155. King M, Marston L, Švab I, Maaroos HI, Geerlings MI, Xavier M, et al. Development and validation of a risk model for prediction of hazardous alcohol consumption in general practice attendees: the predictAL study. PLoS One*.* 2011;6:e22175. <https://doi.org/10.1371/journal.pone.0022175>.

156. Kirchebner J, Gunther MP, Sonnweber M, King A, Lau S. Factors and predictors of length of stay in offenders diagnosed with schizophrenia – A machine-learning-based approach. BMC Psychiatry*.* 2020;20:201. <https://doi.org/10.1186/s12888-020-02612-1>.

157. Klein NS, Holtman GA, Bockting CL, Heymans MW, Burger H. Development and validation of a clinical prediction tool to estimate the individual risk of depressive relapse or recurrence in individuals with recurrent depression. J Psychiatr Res*.* 2018;104:1-7. <https://doi.org/10.1016/j.jpsychires.2018.06.006>.

158. Kotlicka-Antczak M, Karbownik MS, Stawiski K, Pawelczyk A, Zurner N, Pawelczyk T, et al. Short clinically-based prediction model to forecast transition to psychosis in individuals at clinical high risk state. Eur Psychiatry*.* 2019;58:72-79. <https://doi.org/10.1016/j.eurpsy.2019.02.007>.

159. Koutsouleris N, Davatzikos C, Bottlender R, Patschurek-Kliche K, Scheuerecker J, Decker P, et al. Early recognition and disease prediction in the at-risk mental states for psychosis using neurocognitive pattern classification. Schizophr Bull*.* 2012;38:1200-1215. <https://doi.org/10.1093/schbul/sbr037>.

160. Koutsouleris N, Kahn RS, Chekroud AM, Leucht S, Falkai P, Wobrock T, et al. Multisite prediction of 4-week and 52-week treatment outcomes in patients with first-episode psychosis: a machine learning approach. Lancet Psychiatry*.* 2016;3:935-946. <https://doi.org/10.1016/s2215-0366(16)30171-7>.

161. Koutsouleris N, Kambeitz-Ilankovic L, Ruhrmann S, Rosen M, Ruef A, Dwyer DB, et al. Prediction models of functional outcomes for individuals in the clinical high-risk state for psychosis or with recent-onset depression: a multimodal, multisite machine learning analysis. JAMA Psychiatry*.* 2018;75:1156-1172. <https://doi.org/10.1001/jamapsychiatry.2018.2165>.

162. Kwakernaak S, van Mens K, Cahn W, Janssen R. Using machine learning to predict mental healthcare consumption in non-affective psychosis. Schizophr Res*.* 2020;218:166-172. <https://doi.org/10.1016/j.schres.2020.01.008>.

163. Leightley D, Williamson V, Darby J, Fear NT. Identifying probable post-traumatic stress disorder: applying supervised machine learning to data from a UK military cohort. J Ment Health*.* 2019;28:34-41. <https://doi.org/10.1080/09638237.2018.1521946>.

164. Leighton SP, Krishnadas R, Chung K, Blair A, Brown S, Clark S, et al. Predicting one-year outcome in first episode psychosis using machine learning. PLoS One*.* 2019;14:e0212846. <https://doi.org/10.1371/journal.pone.0212846>.

165. Leighton SP, Upthegrove R, Krishnadas R, Benros ME, Broome MR, Gkoutos GV, et al. Development and validation of multivariable prediction models of remission, recovery, and quality of life outcomes in people with first episode psychosis: a machine learning approach. Lancet Digit Health*.* 2019;1:e261-e270. <https://doi.org/10.1016/S2589-7500(19)30121-9>.

166. Lencz T, Smith CW, McLaughlin D, Auther A, Nakayama E, Hovey L, et al. Generalized and specific neurocognitive deficits in prodromal schizophrenia. Biol Psychiatry*.* 2006;59:863-871. <https://doi.org/10.1016/j.biopsych.2005.09.005>.

167. Lenhard F, Sauer S, Andersson E, Månsson KN, Mataix‐Cols D, Rück C, et al. Prediction of outcome in internet‐delivered cognitive behaviour therapy for paediatric obsessive‐compulsive disorder: a machine learning approach. Int J Methods Psychiatr*.* 2018;27:e1576. <https://doi.org/10.1002/mpr.1576>.

168. Lerthattasilp T, Tanprasertkul C, Chunsuwan I. Development of clinical prediction rule for diagnosis of autistic spectrum disorder in children. Ment Illn*.* 2020;12:7-16. <https://doi.org/10.1108/mij-01-2020-0001>.

169. Levin HS, McCauley SR, Josic CP, Boake C, Brown SA, Goodman HS, et al. Predicting depression following mild traumatic brain injury. Arch Gen Psychiatry*.* 2005;62:523-528. <https://doi.org/10.1001/archpsyc.62.5.523>.

170. Lewis SJ, Arseneault L, Caspi A, Fisher HL, Matthews T, Moffitt TE, et al. The epidemiology of trauma and post-traumatic stress disorder in a representative cohort of young people in England and Wales. Lancet Psychiatry*.* 2019;6:247-256. <https://doi.org/10.1016/s2215-0366(19)30031-8>.

171. Librenza-Garcia D, Passos IC, Feiten JG, Lotufo PA, Goulart AC, de Souza Santos I, et al. Prediction of depression cases, incidence, and chronicity in a large occupational cohort using machine learning techniques: an analysis of the ELSA-Brasil study. Psychol Med*.* 2020. <https://doi.org/10.1017/s0033291720001579>.

172. Lin CH, Chou LS, Lin CH, Hsu CY, Chen YS, Lane HY. Early prediction of clinical response in schizophrenia patients receiving the atypical antipsychotic zotepine. J Clin Psychiatry*.* 2007;68:1522-1527. <https://doi.org/10.4088/jcp.v68n1008>.

173. Lin CH, Lane HY, Chen CC, Juo SHH, Yen CF. Early prediction of fluoxetine response for Han Chinese inpatients with major depressive disorder. J Clin Psychopharmacol*.* 2011;31:187-193. <https://doi.org/10.1097/JCP.0b013e318210856f>.

174. Lin CH, Chou LS, Lin CH, Hsu CY, Chen CC, Lane HY. Optimizing the early prediction model for symptomatic remission with short-term treatment for schizophrenia. J Clin Psychopharmacol*.* 2012;32:773-777. <https://doi.org/10.1097/JCP.0b013e318270dfca>.

175. Lin E, Kuo PH, Liu YL, Yu YWY, Yang AC, Tsai SJ. A deep learning approach for predicting antidepressant response in major depression using clinical and genetic biomarkers. Front Psychiatry*.* 2018;9:290. <https://doi.org/10.3389/fpsyt.2018.00290>.

176. Liu Y, Sareen J, Bolton J, Wang J. Development and validation of a risk-prediction algorithm for the recurrence of panic disorder. Depress Anxiety*.* 2015;32:341-348. <https://doi.org/10.1002/da.22359>.

177. Liu R, Yue Y, Jiang H, Lu J, Wu A, Geng D, et al. A risk prediction model for post-stroke depression in Chinese stroke survivors based on clinical and socio-psychological features. Oncotarget*.* 2017;8:62891-62899. <https://doi.org/10.18632/oncotarget.16907>.

178. Lorenzo-Luaces L, DeRubeis RJ, van Straten A, Tiemens B. A prognostic index (PI) as a moderator of outcomes in the treatment of depression: a proof of concept combining multiple variables to inform risk-stratified stepped care models. J Affect Disord*.* 2017;213:78-85. <https://doi.org/10.1016/j.jad.2017.02.010>.

179. Lorimer B, Delgadillo J, Kellett S, Lawrence J. Dynamic prediction and identification of cases at risk of relapse following completion of low-intensity cognitive behavioural therapy. Psychother Res*.* 2021;31:19-32. <https://doi.org/10.1080/10503307.2020.1733127>.

180. Maarsingh OR, Dros J, van der Windt DA, ter Riet G, Schellevis FG, van Weert HC, et al. Diagnostic indicators of anxiety and depression in older dizzy patients in primary care. J Geriatr Psychiatry Neurol*.* 2011;24:98-107. <https://doi.org/10.1177/0891988711405332>.

181. Maarsingh OR, Heymans M, Verhaak P, Penninx B, Comijs H. Development and external validation of a prediction rule for an unfavorable course of late-life depression: a multicenter cohort study. J Affect Disord*.* 2018;235:105-113. <https://doi.org/> 10.1016/j.jad.2018.04.026.

182. Mak A, Tang CS, Chan MF, Cheak AA, Ho RC. Damage accrual, cumulative glucocorticoid dose and depression predict anxiety in patients with systemic lupus erythematosus. Clin Rheumatol*.* 2011;30:795-803. <https://doi.org/10.1007/s10067-010-1651-8>.

183. Mason O, Startup M, Halpin S, Schall U, Conrad A, Carr V. Risk factors for transition to first episode psychosis among individuals with 'at-risk mental states'. Schizophr Res*.* 2004;71:227-237. <https://doi.org/10.1016/j.schres.2004.04.006>.

184. Mechelli A, Lin A, Wood S, McGorry P, Amminger P, Tognin S, et al. Using clinical information to make individualized prognostic predictions in people at ultra high risk for psychosis. Schizophr Res*.* 2017;184:32-38. <https://doi.org/10.1016/j.schres.2016.11.047>.

185. Meehan AJ, Latham RM, Arseneault L, Stahl D, Fisher HL, Danese A. Developing an individualized risk calculator for psychopathology among young people victimized during childhood: a population-representative cohort study. J Affect Disord*.* 2020;262:90-98. <https://doi.org/10.1016/j.jad.2019.10.034>.

186. Morel D, Yu KC, Liu-Ferrara A, Caceres-Suriel AJ, Kurtz SG, Tabak YP. Predicting hospital readmission in patients with mental or substance use disorders: a machine learning approach. Int J Med Inform*.* 2020;139:104136. <https://doi.org/10.1016/j.ijmedinf.2020.104136>.

187. Morrow AS, Campos Vega AD, Zhao X, Liriano MM. Leveraging machine learning to identify predictors of receiving psychosocial treatment for attention deficit/hyperactivity disorder. Adm Policy Ment Health*.* 2020;47:680-692. <https://doi.org/10.1007/s10488-020-01045-y>.

188. Na KS, Cho SE, Geem ZW, Kim YK. Predicting future onset of depression among community dwelling adults in the Republic of Korea using a machine learning algorithm. Neurosci Lett*.* 2020;721:134804. <https://doi.org/10.1016/j.neulet.2020.134804>.

189. Na KS, Geem ZW, Cho SE. Machine learning-based prediction of persistent oppositional defiant behavior for 5 years. Nord J Psychiatry*.* 2020;74:505-510. <https://doi.org/10.1080/08039488.2020.1748711>.

190. Nelson JC, Zhang Q, Deberdt W, Marangell LB, Karamustafalioglu O, Lipkovich IA. Predictors of remission with placebo using an integrated study database from patients with major depressive disorder. Curr Med Res Opin*.* 2012;28:325-334. <https://doi.org/10.1185/03007995.2011.654010>.

191. Nelson B, Yuen HP, Wood SJ, Lin A, Spiliotacopoulos D, Bruxner A, et al. Long-term follow-up of a group at ultra high risk (“prodromal”) for psychosis: the PACE 400 study. JAMA Psychiatry*.* 2013;70:793-802. <https://doi.org/10.1001/jamapsychiatry.2013.1270>.

192. Nichols L, Ryan R, Connor C, Birchwood M, Marshall T. Derivation of a prediction model for a diagnosis of depression in young adults: a matched case-control study using electronic primary care records. Early Interv Psychiatry*.* 2018;12:444-455. <https://doi.org/10.1111/eip.12332>.

193. Nie Z, Vairavan S, Narayan VA, Ye J, Li QS. Predictive modeling of treatment resistant depression using data from STAR*D and an independent clinical study. PLoS One*.* 2018;13:e0197268. <https://doi.org/10.1371/journal.pone.0197268>.

194. Nieman DH, Ruhrmann S, Dragt S, Soen F, van Tricht MJ, Koelman JH, et al. Psychosis prediction: stratification of risk estimation with information-processing and premorbid functioning variables. Schizophr Bull*.* 2014;40:1482-1490. <https://doi.org/10.1093/schbul/sbt145>.

195. Niemann U, Brueggemann P, Boecking B, Mazurek B, Spiliopoulou M. Development and internal validation of a depression severity prediction model for tinnitus patients based on questionnaire responses and socio-demographics. Sci Rep*.* 2020;10:4664. <https://doi.org/10.1038/s41598-020-61593-z>.

196. Nyberg T, Hed Myrberg I, Omerov P, Steineck G, Nyberg U. Depression among parents two to six years following the loss of a child by suicide: a novel prediction model. PLoS One*.* 2016;11:e0164091. <https://doi.org/10.1371/journal.pone.0164091>.

197. Oh J, Yun K, Hwang J-H, Chae J-H. Classification of suicide attempts through a machine learning algorithm based on multiple systemic psychiatric scales. Front Psychiatry*.* 2017;8:192. <https://doi.org/10.3389/fpsyt.2017.00192>.

198. Oh J, Yun K, Maoz U, Kim TS, Chae JH. Identifying depression in the National Health and Nutrition Examination Survey data using a deep learning algorithm. J Affect Disord*.* 2019;257:623-631. <https://doi.org/10.1016/j.jad.2019.06.034>.

199. Okamoto K, Harasawa Y. Prediction of symptomatic depression by discriminant analysis in Japanese community-dwelling elderly. Arch Gerontol Geriatr*.* 2011;52:177-180. <https://doi.org/10.1016/j.archger.2010.03.012>.

200. Papini S, Pisner D, Shumake J, Powers MB, Beevers CG, Rainey EE, et al. Ensemble machine learning prediction of posttraumatic stress disorder screening status after emergency room hospitalization. J Anxiety Disord*.* 2018;60:35-42. <https://doi.org/10.1016/j.janxdis.2018.10.004>.

201. Parikh MN, Li H, He L. Enhancing diagnosis of autism with optimized machine learning models and personal characteristic data. Front Comput Neurosci*.* 2019;13:9. <https://doi.org/10.3389/fncom.2019.00009>.

202. Passos IC, Mwangi B, Cao B, Hamilton JE, Wu M-J, Zhang XY, et al. Identifying a clinical signature of suicidality among patients with mood disorders: a pilot study using a machine learning approach. J Affect Disord*.* 2016;193:109-116. <https://doi.org/10.1016/j.jad.2015.12.066>.

203. Perez Arribas I, Goodwin GM, Geddes JR, Lyons T, Saunders KE. A signature-based machine learning model for distinguishing bipolar disorder and borderline personality disorder. Transl Psychiatry*.* 2018;8:274. <https://doi.org/10.1038/s41398-018-0334-0>.

204. Perlis RH. A clinical risk stratification tool for predicting treatment resistance in major depressive disorder. Biol Psychiatry*.* 2013;74:7-14. <https://doi.org/10.1016/j.biopsych.2012.12.007>.

205. Pradier MF, Hughes MC, McCoy TH, Barroilhet SA, Doshi-Velez F, Perlis RH. Predicting change in diagnosis from major depression to bipolar disorder after antidepressant initiation. Neuropsychopharmacology*.* 2021;46:455-461. <https://doi.org/10.1038/s41386-020-00838-x>.

206. Rabelo-da-Ponte FD, Feiten JG, Mwangi B, Barros FC, Wehrmeister FC, Menezes AM, et al. Early identification of bipolar disorder among young adults – a 22-year community birth cohort. Acta Psychiatr Scand*.* 2020. <https://doi.org/10.1111/acps.13233>.

207. Raket LL, Jaskolowski J, Kinon BJ, Brasen JC, Jönsson L, Wehnert A, et al. Dynamic ElecTronic hEalth reCord deTection (DETECT) of individuals at risk of a first episode of psychosis: a case-control development and validation study. Lancet Digit Health*.* 2020;2:e229-e239. <https://doi.org/10.1016/S2589-7500(20)30024-8>.

208. Reps JM, Cepeda MS, Ryan PB. Wisdom of the CROUD: development and validation of a patient-level prediction model for opioid use disorder using population-level claims data. PloS One*.* 2020;15:e0228632. <https://doi.org/10.1371/journal.pone.0228632>.

209. Rezaii N, Walker E, Wolff P. A machine learning approach to predicting psychosis using semantic density and latent content analysis. NPJ Schizophr*.* 2019;5:9. <https://doi.org/10.1038/s41537-019-0077-9>.

210. Riecher-Rössler A, Pflueger MO, Aston J, Borgwardt SJ, Brewer WJ, Gschwandtner U, et al. Efficacy of using cognitive status in predicting psychosis: a 7-year follow-up. Biol Psychiatry*.* 2009;66:1023-1030. <https://doi.org/10.1016/j.biopsych.2009.07.020>.

211. Rocha TB, Fisher HL, Caye A, Anselmi L, Arseneault L, Barros FC, et al. Identifying adolescents at risk for depression: a prediction score performance in cohorts based in three different continents. J Am Acad Child Adolesc Psychiatry*.* 2021;60:262-273. <https://doi.org/10.1016/j.jaac.2019.12.004>.

212. Roglio VS, Borges EN, Rabelo-da-Ponte FD, Ornell F, Scherer JN, Schuch JB, et al. Prediction of attempted suicide in men and women with crack-cocaine use disorder in Brazil. PloS One*.* 2020;15:e0232242. <https://doi.org/10.1371/journal.pone.0232242>.

213. Rosellini AJ, Dussaillant F, Zubizarreta JR, Kessler RC, Rose S. Predicting posttraumatic stress disorder following a natural disaster. J Psychiatr Res*.* 2018;96:15-22. <https://doi.org/10.1016/j.jpsychires.2017.09.010>.

214. Rosellini AJ, Stein MB, Benedek DM, Bliese PD, Chiu WT, Hwang I, et al. Predeployment predictors of psychiatric disorder-symptoms and interpersonal violence during combat deployment. Depress Anxiety*.* 2018;35:1073-1080. <https://doi.org/10.1002/da.22807>.

215. Rosellini AJ, Liu S, Anderson GN, Sbi S, Tung ES, Knyazhanskaya E. Developing algorithms to predict adult onset internalizing disorders: an ensemble learning approach. J Psychiatr Res*.* 2020;121:189-196. <https://doi.org/10.1016/j.jpsychires.2019.12.006>.

216. Ruhrmann S, Schultze-Lutter F, Salokangas RK, Heinimaa M, Linszen D, Dingemans P, et al. Prediction of psychosis in adolescents and young adults at high risk: results from the prospective European prediction of psychosis study. Arch Gen Psychiatry*.* 2010;67:241-251. <https://doi.org/10.1001/archgenpsychiatry.2009.206>.

217. Russo J, Katon W, Zatzick D. The development of a population-based automated screening procedure for PTSD in acutely injured hospitalized trauma survivors. Gen Hosp Psychiatry*.* 2013;35:485-491. <https://doi.org/10.1016/j.genhosppsych.2013.04.016>.

218. Sau A, Bhakta I. Artificial neural network (ANN) model to predict depression among geriatric population at a slum in Kolkata, India. J Clin Diagn Res*.* 2017;11:VC01-VC04. <https://doi.org/10.7860/jcdr/2017/23656.9762>.

219. Sau A, Bhakta I. Predicting anxiety and depression in elderly patients using machine learning technology. Healthc Technol Lett*.* 2017;4:238-243. <https://doi.org/10.1049/HTL.2016.0096>.

220. Saxe GN, Ma S, Ren J, Aliferis C. Machine learning methods to predict child posttraumatic stress: a proof of concept study. BMC Psychiatry*.* 2017;17:223. <https://doi.org/10.1186/s12888-017-1384-1>.

221. Schepers V, Post M, Visser-Meily A, van de Port I, Akhmouch M, Lindeman E. Prediction of depressive symptoms up to three years post-stroke. J Rehabil Med*.* 2009;41:930-935. <https://doi.org/10.2340/16501977-0446>.

222. Schultebraucks K, Qian M, Abu-Amara D, Dean K, Laska E, Siegel C, et al. Pre-deployment risk factors for PTSD in active-duty personnel deployed to Afghanistan: a machine-learning approach for analyzing multivariate predictors. Mol Psychiatry*.* 2020. <https://doi.org/10.1038/s41380-020-0789-2>.

223. Schultebraucks K, Shalev AY, Michopoulos V, Grudzen CR, Shin SM, Stevens JS, et al. A validated predictive algorithm of post-traumatic stress course following emergency department admission after a traumatic stressor. Nat Med*.* 2020;26:1084-1088. <https://doi.org/10.1038/s41591-020-0951-z>.

224. Serretti A, Olgiati P, Liebman MN, Hu H, Zhang Y, Zanardi R, et al. Clinical prediction of antidepressant response in mood disorders: linear multivariate vs. neural network models. Psychiatry Res*.* 2007;152:223-231. <https://doi.org/10.1016/j.psychres.2006.07.009>.

225. Setyawan J, Yang H, Cheng D, Cai X, Signorovitch J, Xie J, et al. Developing a risk score to guide individualized treatment selection in attention deficit/hyperactivity disorder. Value Health*.* 2015;18:824-831. <https://doi.org/10.1016/j.jval.2015.06.005>.

226. Shalev AY, Gevonden M, Ratanatharathorn A, Laska E, Van Der Mei WF, Qi W, et al. Estimating the risk of PTSD in recent trauma survivors: results of the International Consortium to Predict PTSD (ICPP). World Psychiatry*.* 2019;18:77-87. <https://doi.org/10.1002/wps.20608>.

227. Silveira Jr ÉdM, Passos IC, Scott J, Bristot G, Scotton E, Mendes LST, et al. Decoding rumination: a machine learning approach to a transdiagnostic sample of outpatients with anxiety, mood and psychotic disorders. J Psychiatr Res*.* 2020;121:207-213. <https://doi.org/10.1016/j.jpsychires.2019.12.005>.

228. Silverstein M, Hironaka LK, Feinberg E, Sandler J, Pellicer M, Chen N, et al. Using clinical data to predict accurate ADHD diagnoses among urban children. Clin Pediatr*.* 2016;55:326-332. <https://doi.org/10.1177/0009922815591882>.

229. Simon GE, Johnson E, Lawrence JM, Rossom RC, Ahmedani B, Lynch FL, et al. Predicting suicide attempts and suicide deaths following outpatient visits using electronic health records. Am J Psychiatry*.* 2018;175:951-960. <https://doi.org/10.1176/appi.ajp.2018.17101167>.

230. Slobodin O, Yahav I, Berger I. A machine-based prediction model of ADHD using CPT data. Front Hum Neurosci*.* 2020;14:560021. <https://doi.org/10.3389/fnhum.2020.560021>.

231. Studerus E, Beck K, Fusar-Poli P, Riecher-Rössler A. Development and validation of a dynamic risk prediction model to forecast psychosis onset in patients at clinical high risk. Schizophr Bull*.* 2020;46:252-260. <https://doi.org/10.1093/schbul/sbz059>.

232. Thompson A, Nelson B, Yung A. Predictive validity of clinical variables in the "at risk" for psychosis population: international comparison with results from the North American Prodrome Longitudinal Study. Schizophr Res*.* 2011;126:51-57. <https://doi.org/10.1016/j.schres.2010.09.024>.

233. Tondo L, Visioli C, Preti A, Baldessarini RJ. Bipolar disorders following initial depression: modeling predictive clinical factors. J Affect Disord*.* 2014;167:44-49. <https://doi.org/10.1016/j.jad.2014.05.043>.

234. Tulloch A, David A, Thornicroft G. Exploring the predictors of early readmission to psychiatric hospital. Epidemiol Psychiatr Sci*.* 2016;25:181-193. <https://doi.org/10.1017/S2045796015000128>.

235. Usta MB, Karabekiroglu K, Sahin B, Aydin M, Bozkurt A, Karaosman T, et al. Use of machine learning methods in prediction of short-term outcome in autism spectrum disorders. Psychiatr Clin Psychopharmacol*.* 2019;29:320-325. <https://doi.org/10.1080/24750573.2018.1545334>.

236. van Breda W, Bremer V, Becker D, Hoogendoorn M, Funk B, Ruwaard J, et al. Predicting therapy success for treatment as usual and blended treatment in the domain of depression. Internet Interv*.* 2018;12:100-104. <https://doi.org/10.1016/j.invent.2017.08.003>.

237. van der Aa HP, Xie J, Rees G, Fenwick E, Holloway EE, van Rens GH, et al. Validated prediction model of depression in visually impaired older adults. Ophthalmology*.* 2016;123:1164-1166. <https://doi.org/10.1016/j.ophtha.2015.11.028>.

238. van Heumen MA, Hollander MH, van Pampus MG, van Dillen J, Stramrood CAI. Psychosocial predictors of postpartum posttraumatic stress disorder in women with a traumatic childbirth experience. Front Psychiatry*.* 2018;9:348. <https://doi.org/10.3389/fpsyt.2018.00348>.

239. van Loo HM, Aggen SH, Gardner CO, Kendler KS. Multiple risk factors predict recurrence of major depressive disorder in women. J Affect Disord*.* 2015;180:52-61. <https://doi.org/10.1016/j.jad.2015.03.045>.

240. van Loo HM, Aggen SH, Gardner CO, Kendler KS. Sex similarities and differences in risk factors for recurrence of major depression. Psychol Med*.* 2018;48:1685-1693. <https://doi.org/10.1017/S0033291717003178>.

241. van Voorhees BW, Paunesku D, Gollan J, Kuwabara S, Reinecke M, Basu A. Predicting future risk of depressive episode in adolescents: the Chicago Adolescent Depression Risk Assessment (CADRA). Ann Fam Med*.* 2008;6:503-511. <https://doi.org/10.1370/afm.887>.

242. Vöhringer PA, Jimenez MI, Igor MA, Fores GA, Correa MO, Sullivan MC, et al. A clinical predictive score for mood disorder risk in low-income primary care settings. J Affect Disord*.* 2013;151:1125-1131. <https://doi.org/10.1016/j.jad.2013.06.056>.

243. Vöhringer PA, Barroilhet SA, Alvear K, Medina S, Espinosa C, Alexandrovich K, et al. The International Mood Network (IMN) Nosology Project: differentiating borderline personality from bipolar illness. Acta Psychiatr Scand*.* 2016;134:504-510. <https://doi.org/10.1111/acps.12643>.

244. Wang JL, Manuel D, Williams J, Schmitz N, Gilmour H, Patten S, et al. Development and validation of prediction algorithms for major depressive episode in the general population. J Affect Disord*.* 2013;151:39-45. <https://doi.org/10.1016/j.jad.2013.05.045>.

245. Wang JL, Patten S, Sareen J, Bolton J, Schmitz N, MacQueen G. Development and validation of a prediction algorithm for use by health professionals in prediction of recurrence of major depression. Depress Anxiety*.* 2014;31:451-457. <https://doi.org/10.1002/da.22215>.

246. Wang JL, Sareen J, Patten S, Bolton J, Schmitz N, Birney A. A prediction algorithm for first onset of major depression in the general population: development and validation. J Epidemiol Community Health*.* 2014;68:418-424. <https://doi.org/10.1136/jech-2013-202845>.

247. Wang S, Pathak J, Zhang Y. Using electronic health records and machine learning to predict postpartum depression. Stud Health Technol Inform*.* 2019;264:888-892. <https://doi.org/10.3233/shti190351>.

248. Wang KZ, Fatemi AB, Adanty C, Harripaul R, Griffiths J, Kolla N, et al. Prediction of physical violence in schizophrenia with machine learning algorithms. Psychiatry Res*.* 2020;289:112960. <https://doi.org/10.1016/j.psychres.2020.112960>.

249. Wolf A, Fanshawe TR, Sariaslan A, Cornish R, Larsson H, Fazel S. Prediction of violent crime on discharge from secure psychiatric hospitals: a clinical prediction rule (FoVOx). Eur Psychiatry*.* 2018;47:88-93. <https://doi.org/10.1016/j.eurpsy.2017.07.011>.

250. Wshah S, Skalka C, Price M. Predicting posttraumatic stress disorder risk: a machine learning approach. JMIR Ment Health*.* 2019;6:e13946. <https://doi.org/10.2196/13946>.

251. Wu M-J, Passos IC, Bauer IE, Lavagnino L, Cao B, Zunta-Soares GB, et al. Individualized identification of euthymic bipolar disorder using the Cambridge Neuropsychological Test Automated Battery (CANTAB) and machine learning. J Affect Disord*.* 2016;192:219-225. <https://doi.org/10.1016/j.jad.2015.12.053>.

252. Xu Z, Zhang Q, Li W, Li M, Yip PSF. Individualized prediction of depressive disorder in the elderly: a multitask deep learning approach. Int J Med Inform*.* 2019;132:103973. <https://doi.org/10.1016/j.ijmedinf.2019.103973>.

253. Yung AR, Phillips LJ, Yuen HP, Francey SM, McFarlane CA, Hallgren M, et al. Psychosis prediction: 12-month follow up of a high-risk ("prodromal") group. Schizophr Res*.* 2003;60:21-32. <https://doi.org/10.1016/s0920-9964(02)00167-6>.

254. Yung AR, Phillips LJ, Yuen HP, McGorry PD. Risk factors for psychosis in an ultra high-risk group: psychopathology and clinical features. Schizophr Res*.* 2004;67:131-142. <https://doi.org/10.1016/s0920-9964(03)00192-0>.

255. Zhang T, Xu L, Tang Y, Li H, Tang X, Cui H, et al. Prediction of psychosis in prodrome: development and validation of a simple, personalized risk calculator. Psychol Med*.* 2019;49:1990-1998. <https://doi.org/10.1017/s0033291718002738>.

256. Zhang T, Xu L, Li H, Woodberry KA, Kline ER, Jiang J, et al. Calculating individualized risk components using a mobile app-based risk calculator for clinical high risk of psychosis: findings from ShangHai At Risk for Psychosis (SHARP) program. Psychol Med*.* 2019. <https://doi.org/10.1017/s003329171900360x>.

257. Zhang W, Liu H, Silenzio VMB, Qiu P, Gong W. Machine learning models for the prediction of postpartum depression: application and comparison based on a cohort study. JMIR Med Inform*.* 2020;8:e15516. <https://doi.org/10.2196/15516>.

258. Ziermans T, de Wit S, Schothorst P, Sprong M, van Engeland H, Kahn R, et al. Neurocognitive and clinical predictors of long-term outcome in adolescents at ultra-high risk for psychosis: a 6-year follow-up. PLoS One*.* 2014;9:e93994. <https://doi.org/10.1371/journal.pone.0093994>.

259. Zuithoff NP, Vergouwe Y, King M, Nazareth I, Hak E, Moons KG, et al. A clinical prediction rule for detecting major depressive disorder in primary care: the PREDICT-NL study. Fam Pract*.* 2009;26:241-250. <https://doi.org/10.1093/fampra/cmp036>.

260. Brathwaite R, Rocha TB, Kieling C, Gautam K, Koirala S, Mondelli V, et al. Predicting the risk of depression among adolescents in Nepal using a model developed in Brazil: the IDEA Project. Eur Child Adolesc Psychiatry*.* 2020. <https://doi.org/10.1007/s00787-020-01505-8>.

261. Carrión RE, Cornblatt BA, Burton CZ, Tso IF, Auther AM, Adelsheim S, et al. Personalized prediction of psychosis: external validation of the NAPLS-2 psychosis risk calculator with the EDIPPP project. Am J Psychiatry*.* 2016;173:989-996. <https://doi.org/10.1176/appi.ajp.2016.15121565>.

262. Fusar-Poli P, Werbeloff N, Rutigliano G, Oliver D, Davies C, Stahl D, et al. Transdiagnostic risk calculator for the automatic detection of individuals at risk and the prediction of psychosis: second replication in an independent national health service trust. Schizophr Bull*.* 2019;45:562-570. <https://doi.org/10.1093/schbul/sby070>.

263. King M, Bottomley C, Bellón-Saameño J, Torres-Gonzalez F, Svab I, Rotar D, et al. Predicting onset of major depression in general practice attendees in Europe: extending the application of the predictD risk algorithm from 12 to 24 months. Psychol Med*.* 2013;43:1929-1939. <https://doi.org/10.1017/s0033291712002693>.

264. Negatsch V, Voulgaris A, Seidel P, Roehle R, Opitz-Welke A. Identifying violent behavior using the Oxford Mental Illness and Violence Tool in a psychiatric ward of a German prison hospital. Front Psychiatry*.* 2019;10:264. <https://doi.org/10.3389/fpsyt.2019.00264>.

265. Nigatu YT, Liu Y, Wang J. External validation of the international risk prediction algorithm for major depressive episode in the US general population: the PredictD-US study. BMC Psychiatry*.* 2016;16:256. <https://doi.org/10.1186/s12888-016-0971-x>.

266. Nigatu YT, Wang J. External validation of the international risk prediction algorithm for the onset of generalized anxiety and/or panic syndromes (the Predict A) in the US general population. J Anxiety Disord*.* 2019;64:40-44. <https://doi.org/10.1016/j.janxdis.2019.03.004>.

267. Osborne KJ, Mittal VA. External validation and extension of the NAPLS-2 and SIPS-RC personalized risk calculators in an independent clinical high-risk sample. Psychiatry Res*.* 2019;279:9-14. <https://doi.org/10.1016/j.psychres.2019.06.034>.

268. Oliver D, Spada G, Colling C, Broadbent M, Baldwin H, Patel R, et al. Real-world implementation of precision psychiatry: transdiagnostic risk calculator for the automatic detection of individuals at-risk of psychosis. Schizophr Res*.* 2021;227:52-60. <https://doi.org/10.1016/j.schres.2020.05.007>.

269. Wang T, Oliver D, Msosa Y, Colling C, Spada G, Roguski Ł, et al. Implementation of a real-time psychosis risk detection and alerting system based on electronic health records using CogStack. J Vis Exp*.* 2020;159:e60794. <https://doi.org/10.3791/60794>.
